# Synthesis and cytotoxic activity of madecassic acid–silybin conjugate compounds in liver cancer cells†

**DOI:** 10.1039/d4md00170b

**Published:** 2024-08-02

**Authors:** Chien Van Tran, Thao Thi Phuong Tran, Anh The Nguyen, Loc Van Tran, Ninh Thi Pham, Luu Thi Nguyen, Dung Thi Nguyen, Michelle D. Garrett, Nga Thi Nguyen, Thao Thi Do, Christopher J. Serpell, Sung Van Tran

**Affiliations:** a Institute of Chemistry, Vietnam Academy of Science and Technology 18 Hoang Quoc Viet Road, Cau Giay Hanoi Vietnam tranvansungvhh@gmail.com; b Graduate University of Science and Technology, Vietnam Academy of Science and Technology 18 Hoang Quoc Viet Road, Cau Giay Hanoi Vietnam; c School of Biosciences, University of Kent Stacey Building, Canterbury Kent CT2 7NJ UK; d Institute of Biotechnology, Vietnam Academy of Science and Technology 18 Hoang Quoc Viet Road, Cau Giay Hanoi Vietnam; e Department of Pharmaceutical and Biological Chemistry, School of Pharmacy, University College London 29-39 Brunswick Square London WC1N 1AX UK chris.serpell@ucl.ac.uk

## Abstract

A series of 14 conjugates of 2α,3β,23-triacetyl-madecassic acid and silybin were designed and synthesized. The madecassic acid unit was linked to silybin either directly at position C-7 or C-3; or through an amino acid linker (glycine, β-alanine, or 11-aminoundecanoic acid) at position C-3. The conjugates were tested *in vitro* for their cytotoxic effect on HepG2 cells using the MTT assay. The results confirmed that the conjugated compounds demonstrated a stronger cytotoxic effect compared to the parent compounds. Of these compounds, the most promising conjugate, compound 8, was evaluated for cytotoxic activity in the additional Hep3B, Huh7, and Huh7R human hepatocellular carcinoma cell lines and also for cell cycle changes and induction of apoptosis in HepG2 cells. This compound caused a rapid and significant induction of caspase 3 activity and induced cell cycle arrest in the S phase – effects distinct from the activity of madecassic acid. This is the first study on the synthesis and cytotoxicity of madecassic acid–silybin conjugates, and of their testing against liver cancer cell lines and provides evidence for a distinct biological profile *versus* madecassic acid alone.

## Abstract 2

Madecassic acid and silybin have been conjoined to produce hybrid compounds with improved and different activity against liver cancer cells.

## Graphical abstract

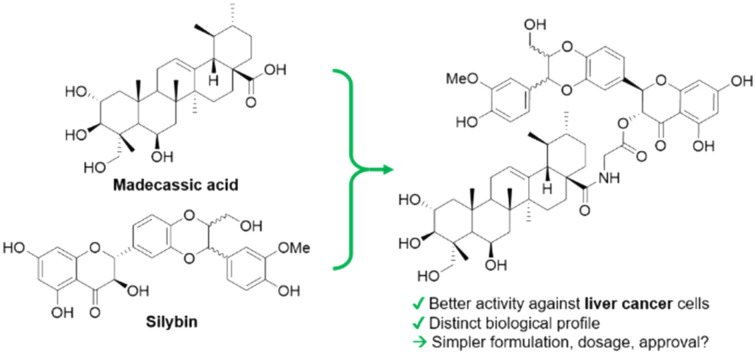

## 1. Introduction

Liver cancer was one of the top five causes of cancer death in 185 countries in 2020, with an estimated 906 000 new cases and 830 000 deaths globally. The number of liver cancer deaths is predicted to reach approximately 1.3 million people by 2040, representing a 56.4% increase upon 2020.^1^ Liver cancer is caused by viral infections including hepatitis B virus (HBV), hepatitis C virus (HCV), and hepatitis D virus (HDV), and is becoming one of the most challenging and urgent problems particularly in Viet Nam today. An estimated 25 000 new cases of liver cancer were reported in 2020, making Viet Nam the fifth highest country in the world for incidence of this disease.^1–3^ Since 2018, liver cancer has risen above lung cancer to become the leading cause of cancer death in Viet Nam.^2,3^

The herb *Centella asiatica* (L.) Urb. (Apiaceae) is extensively grown throughout Viet Nam. This medicinal plant has a wide range of biological properties, including antioxidant, anticancer, and wound healing activity.^4–6^ Madecassic acid is one of the three main pentacyclic triterpenoid acids produced by the plant, together with asiatic acid and terminolic acid.^7^ Madecassic acid has been reported to have valuable pharmacological activities, such as wound healing,^8^ antioxidant,^9^ anti-inflammatory,^10^ and antidiabetic effects.^11^ Recently, madecassic acid has been shown to induce apoptosis in the CT26 mouse colon cancer cell line and its anticancer activity can be enhanced through chemical modification. Indeed, new madecassic acid derivatives have been reported to have significantly increased cytotoxicity towards cancer cell lines, including hepatocellular carcinoma models.^12,13^

Silybins (also known as silibinins) A and B are flavonolignan framework compounds isolated from the seeds of *Silybum marianum* (L.) Gaertn. (Asteraceae), and are well-known for their hepatoprotective activity,^14^ strong antioxidant potential and anti-lipid peroxidation action.^15,16^ Recent studies have found that silybins possess cytotoxic activity in a mouse model of prostate cancer,^17^ in the human non-small-cell lung carcinoma H1299, H322 and H460 cell lines,^18^ and the SW480 human colorectal cancer cell line.^19^ In addition, they have proven cytotoxic potential in bladder, skin, prostate, colon and lung cancers^20,21^ in which they regulate the cancer cell cycle, apoptosis, and autophagy, as well as inhibiting tumor-inducing factors, potentially through the HGF/c-Met, Wnt/β-catenin, and PI3K/Akt/mTOR signaling pathways.^22,23^ Structural modifications of silybin could provide further interesting information on the mechanism of action of this molecule and hence further potential applications.

Synthesis of hybrid molecules composed of multiple bioactive compounds into a single molecule could be an effective strategy to produce novel and better active substances for the treatment of cancer.^24^ Bio-conjugated molecules could have superior efficacy compared to a single drug due to the minimization of unwanted side-effects and synergism of two or more active moieties in one molecule, which enables both to be delivered at the same time and to the same place.^25–27^ Although this will necessarily result in larger molecules, which are classically expected to have poorer pharmacokinetics, there is an increasing awareness that molecules beyond the usual rules for drug design are worth exploring.^28^

**Fig. 1:**
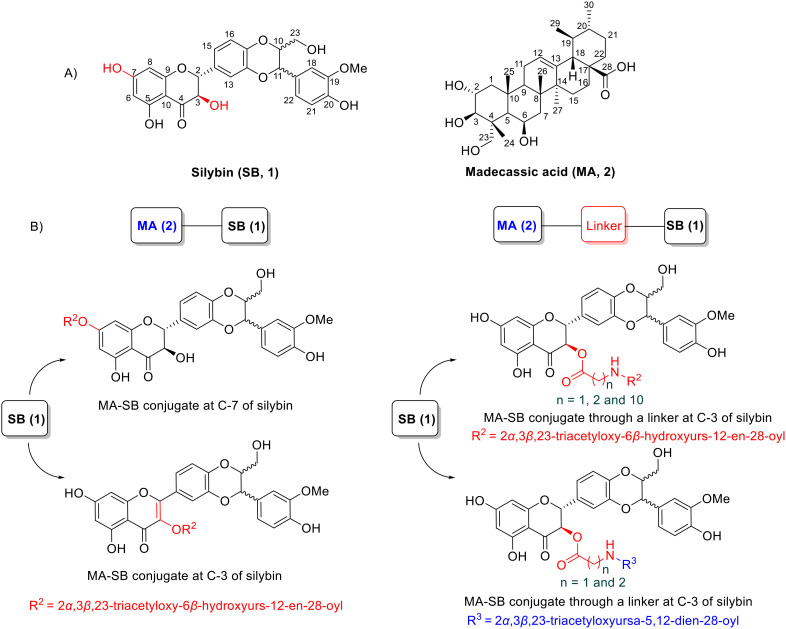
A) Chemical structures of silybin (1, A/B designation depends on variable stereochemistry) and madecassic acid (2); B) rational design of MA–SB conjugates.

Inspired by the potential of hybrid natural product compounds, we here report the design and synthesis of a new set of madecassic acid-silybin conjugates which were anticipated to be able to both kill liver cancer cells and provide protection to healthy hepatocytes, potentially reducing side effects. These hybrids are linked through an ester bond between a hydroxy group at C-7 or C-3 of silybin and the carboxyl group of madecassic acid, or *via* an amino acid as a spacer (Fig. 1). The synthesized compounds were evaluated for their antiproliferative potency *in vitro* against the HepG2 human liver cancer cell line. The most potent compound, 8, was further evaluated for cytotoxic activity in Hep3B, Huh7, and Huh7R liver cancer cell lines together with cell cycle analysis.

## 2. Results and discussion

### 2.1. Chemistry

**Scheme 1:**
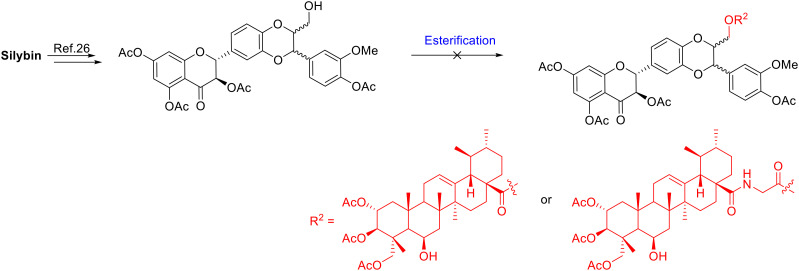
Selective esterification of 2α,3β,23-triacetyl-madecassic acid with silybin at position 23.

Silybin (1) is a flavonoligan containing five functional hydroxyl groups, therefore regioselective esterification between the carboxylic acid group of madecassic acid (MA, 2) and a single alcohol on the silybin moiety may be somewhat problematic (Fig. 1). Initially, our aim was to conjugate madecassic acid with the hydroxy group at C-23 of silybin through an ester. Accordingly, silybin was converted into 3,5,7,20-*O*-tetraacetyl silybin as described by Armando,^29^ followed by reaction with the carboxyl of 2α,3β,23-triacetyl-madecassic acid (3) or its glycine derivatives (Scheme 1). Various esterification conditions were applied, such as Staab's reagent (1,1′-carbonyldiimidazole/4-dimethylaminopyridine), Steglich esterification (DCC/DMAP/THF),^30^ or the Mitsunobu reaction (DEAD/Ph_3_P),^31,32^ but these attempts failed to obtain the desired ester.

Due to the failure of 3,5,7,20-*O*-tetraacetylsilybin to participate in selective esterification, free silybin, without any protection, was used as the starting material. As reported by Li^33^ and Decker^34^ regioselective 23-OH esterification of the silybin moiety is possible under Mitsunobu conditions. However, in our case the esterification did not occur as planned with MA or its derivatives. Further alternative routes were therefore pursued.

**Scheme 2:**
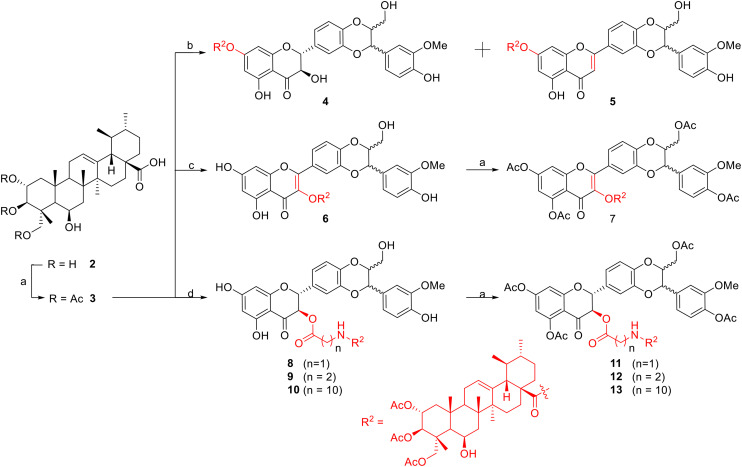
Synthesis of madecassic acid conjugated with silybin. Reagents and conditions: a) Ac_2_O, pyridine, rt, 12 h 84%; b) i) oxalyl chloride, DCM, 18 h, rt, ii) TEA, DCM, silybin, rt, 4 (40%), 5 (20%); c) Silybin, Dicyclohexylcarbodiimide (DCC), DMAP, 0 °C, rt, 34 h, 42%; d) i) oxalyl chloride, DCM, rt, 14 h, ii) amino acid (glycine; or β-alanine; or 11-aminoundecanoic acid (11-AUDA)), TEA, DCM, rt, 18 h; iii) Silybin, DCC, DMAP, THF, 0 °C, rt, 18 h.

As illustrated in Scheme 2, treatment of acid 3 with either thionyl chloride or oxalyl chloride to produce an intermediate MA–chloride acid followed by exposure to silybin (1) in the presence of triethylamine (TEA) led to the formation of esters 4 and 5, in which NMR analysis of compound 5 showed a dehydration of the hydroxyl group at C-3 on silybin by the appearance of a signal of a singlet proton resonance for H-3 at *δ*_H_ 6.56, and carbon resonance at C-3 at *δ*_C_ 104.38 (3-CH).^31^ The selective attachment to the 7-OH group of silybin was confirmed based on NMR analysis, in agreement with the report by Křen and Decker.^34,35^ On the other hand, under Steglich conditions using DCC/DMAP, esterification of acid 3 with silybin 1 provided ester 6 with a selective attachment to the 3-OH group of silybin. The NMR analysis of ester 6 confirmed an elimination of protons H-2 and H-3 on the silybin moiety, which took part in an oxidation under mild basic conditions to create 2,3-dehydrosilybin derivatives. The conclusion was based on the disappearance of proton signals in the NMR at *δ*_H_ 5.0 (d, *J* = 11.5 Hz, H-2) and 4.54 (dd, *J* = 3.5; 11.5 Hz, H-3), together with carbon signals at *δ*_C_ 84.7 (C-2) and at *δ*_C_ 73.7 (C-3),^35^ accompanied by the appearance of two new carbon signals upfield at *δ*_C_ 156.7 (C-2) and 132.5 (C-3).^36,37^

The conjugation of 2α,3β,23-triacetyl-madecassic acid with silybin *via* an amino acid as a spacer (glycine, β-alanine, or 11-aminoundecanoic acid (11-AUDA)), was carried out in a two-step reaction sequence. Treatment of triacetyl madecassic acid (3) with oxalyl chloride to furnish the intermediate acyl chloride acid was followed by reaction with one of the three amino acids and then subsequent coupling with silybin 1 under Steglich conditions, using DCC/DMAP in dry tetrahydrofuran (THF) at room temperature. The corresponding conjugates 8–10 were obtained in overall yields of 20–26%. Analysis of NMR spectroscopy of these conjugates 8–10 confirmed regioselective esterification to the 3-OH group of the silybin moiety. A comparison of the ^1^H NMR spectra of the silybin (1) with its conjugated ester revealed that the chemical shift of H-3 proton multiplet in the silybin moiety had shifted downfield from a range of 4.65–4.64 ppm^38^ up to 5.82–5.84 ppm. To date, reported silybin ester derivatives at position C-3 are rare: Antoszczak *et al.* reported the synthesis of conjugates of silybin with the antibiotics salinomucin and monensin. The authors obtained the conjugates through an ester linkage in the 23-OH group with yields of 43% and 35%, respectively, and no conjugates at the 3-OH group of silybin.^39^ However, our results are in good agreement with the results by Křen^30^ who reported a selective attachment to the 3-OH group of the silybin moiety under Steglich esterification conditions to form 3-*O*-galloylsilybin. Acetylation of compounds 6, 8–10 with acetic anhydride in pyridine at room temperature provided the corresponding acetylated products (7, 11–13). Their structures were confirmed by the analysis of the NMR and MS spectroscopic data.

**Scheme 3:**
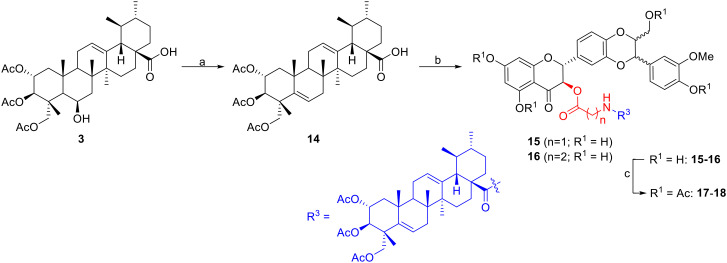
Synthesis of dehydrated madecassic acid conjugated with silybin. Reagents and conditions: a) pyridine, SOCl_2_, pyridine, 2 h, rt, 47%. b) i) Oxalyl chloride, DCM, rt, 14 h, ii) amino acid (glycine; or β-alanine), TEA, DCM, rt, 18 h; iii) silybin, dicyclohexylcarbodiimide (DCC), DMAP, THF, 0 °C, rt, 18 h; c) Ac_2_O, pyridine, rt, 12 h.

In order to evaluate the role of the 6-OH group in madecassic acid in cytotoxicity to HepG2 cells, a series of conjugates 15–18 were synthesized (Scheme 3). Triacetyl madecassic acid (3) was first treated with thionyl chloride in the presence of pyridine to give the dehydrated compound 14 (ref. 12) in 47% yield after a silica gel column chromatography. In a similar two-step sequence reaction, conversion of acid 14 into the intermediate chloride acid followed by coupling with amino acids (glycine; or β-alanine), and subsequently esterified with silybin (1) under Steglich conditions provided esters 15–16 (21–23%). Treatment of these esters with acetic anhydride provided products 17 and 18 in 68% and 58% yields, respectively, after silica gel column chromatography (Scheme 3).

### 2.2. Biological activities

#### 2.2.1. Cytotoxic activity

All compounds synthesized from silybin and madecassic acid were first screened for their anti-proliferative activity in the HepG2 human hepatocellular (liver) carcinoma cell line using the MTT assay. As shown in Table 1, the GI_50_ values for the 18 compounds in HepG2 cells were diverse. Several compounds showed significantly higher antiproliferative activity in HepG2 *versus* the natural compounds silybin (1) and madecassic acid (2), whilst others exhibited weaker activity. To illustrate, compounds 3, 8, 11 and 14 showed lower GI_50_ values and therefore improved antiproliferative activity compared to those of both silybin (1) and madecassic acid (2), and notably neither 3 nor 14 contain a silybin unit. In contrast, compounds 4, 5, 7, 10, 12, 16 and 18 showed reduced antiproliferative activity (higher GI_50_ values), *versus* the two starting compounds and 4 and 5 (conjugated to silybin at position 7) had GI_50_ values greater than 500 μM. The other group including compounds 6, 9, 13, 15, 17 displayed improved antiproliferative activity *versus* silybin, but not madecassic acid.

**Table 1:** Cytotoxic activity of madecassic acid (2), silybin (1) and their conjugated madecassic acid-silybin derivatives on the HepG2 human hepatocellular carcinoma cell line

Compound	Conjugation position^a^	Linkage	Modification^b^	GI_50_ (μM)
Silybin (1)	—	—	—	285.40 ± 1.62
Madecassic acid (2)	—	—	—	161.0 ± 1.28
3	—	—	+Ac	44.12 ± 2.08
4	7	Direct ester	—	>500
5	7	Direct ester	−H_2_O(S)	>500
6	3	Direct ester	—	217.37 ± 10.76
7	3	Direct ester	+Ac	471.13 ± 17.82
8	3	Glycine	—	32.5 ± 3.59
9	3	β-Alanine	—	141.37 ± 39.22
10	3	11-AUDA	—	383.12 ± 40.69
11	3	Glycine	+Ac	122.48 ± 4.66
12	3	β-Alanine	+Ac	359.62 ± 12.35
13	3	11-AUDA	+Ac	198.93 ± 48.55
14	—	—	−H_2_O(M)	38.47 ± 5.78
15	3	Glycine	−H_2_O(M)	173.50 ± 96.94
16	3	β-Alanine	−H_2_O(M)	343.62 ± 19.12
17	3	Glycine	−H_2_O(M) +Ac	243.41 ± 21.96
18	3	β-Alanine	−H_2_O(M) +Ac	352.08 ± 33.11
Ellipticine	—	—	—	0.42 ± 0.01

a Alcohol on the silybin unit through which conjugation is achieved.

b +Ac = acetylation, −H_2_O = dehydration on 2,3 positions on silybin (S) or 5,6 positions on madecassic acid (M). Ellipticine was used as a positive control and all GI_50_ values were generated using a 96 hour MTT assay (*n* ≥ 3 assay repeats).

Patterns in the data were identified: acetylation of the hydroxyl groups at positions 2α,3β,23 of both madecassic acid and its dehydrated product (3 and 14) significantly increased antiproliferative activity on the HepG2 cell line. The conjugates between silybin or tetra-acetyl silybin with triacetyl madecassic acid (3) using glycine as a spacer also enhanced this activity (compounds 8 and 11), and the compound with silybin displaying free hydroxyls (8) had greater activity *versus* the equivalent acetylated version (11).

#### 2.2.2. Cytotoxicity to Hep3B, Huh7 and Huh7R

Of all the compounds evaluated on HepG2 cells, compound 8 exhibited the highest cytotoxic activity and was chosen for further evaluation on three additional hepatocellular carcinoma cell lines: Hep3B, Huh7, and Huh7R, with the results compared to those of madecassic acid (2), using the MTT assay (Table 2). The antiproliferative activity of compound 8 was much higher than that of madecassic acid across all cell lines, with its GI_50_ decreasing by multiples of 18.5, 14.3, and 15.1 respectively, for Hep3B, Huh7 and Huh7R *versus* the parent compound. Compound 8 suppressed proliferation of the Hep3B cell line the most effectively *versus* the Huh7 and Huh7R cell lines. The effectiveness of compound 8 on these three cell lines was found to be in the same order of ranking by GI_50_ value as for madecassic acid (2) *i.e.* Hep3B lowest and Huh7R highest GI_50_ value.

**Table 2:** Cytotoxic activity of madecassic acid (2) and compound 8 on Hep3B, Huh7, and Huh7R hepatocellular carcinoma cell lines (96 hour MTT assay, *n* ≥ 3 assay repeats)

Compound	GI_50_ (μM)		
	Hep3B	Huh7	Huh7R
Madecassic acid (2)	177.80 ± 7.36	197.90 ± 9.80	215.43 ± 4.14
8	9.61 ± 0.81	13.78 ± 0.45	14.23 ± 1.74

#### 2.2.3. Apoptotic induction

**Fig. 2:**
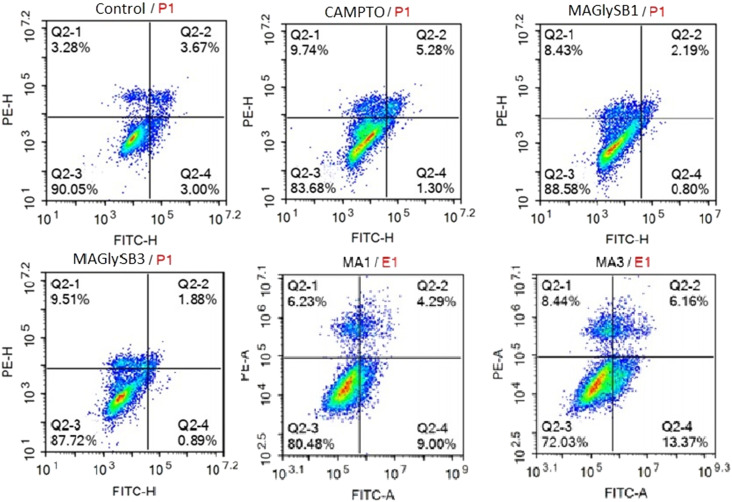
The effect of MA (2) and compound 8 on early and late apoptosis and necrosis. HepG2 cells were incubated with compounds at 1 × GI_50_ and 3 × GI_50_ for each compound for 24 hours followed by annexin V-FITC and PI staining and analysis by flow cytometry. Camptothecin treated cells were used a positive control with untreated cells as the negative control.

Based on the strong anti-proliferative activity of compound 8 on the four hepatocellular carcinoma cell lines, its ability to induce apoptosis was investigated. This was evaluated through measurement of annexin V-FITC and PI staining of HepG2 cells, 24 hours after incubation with compound followed by flow cytometry for detection of these markers. The results (Table 3, Fig. 2) showed that HepG2 cells treated with madecassic acid (2) at 1 × GI_50_ are found to be more in early and late apoptosis when compared to the untreated control (9.00 and 4.29%, respectively) and that increasing the MA concentration to 3 × GI_50_ increased the proportion of cells in both early and late apoptosis further (13.37 and 6.16%, respectively). This result is consistent with the reported induction of apoptosis by MA when colon cancer cells are treated with this compound. Compound 8 at 1 × GI_50_ resulted in induction of early and late apoptosis of 10.6% and 4.92%, respectively, along with a strong necrotic signal of 13.32%, whilst at 3 × GI_50_ early and late apoptosis values were lower than the untreated control, and necrosis was still increased (13.81%).

**Table 3:** The effect of madecassic acid (2) and compound 8 on apoptosis

Compound	Live	Cells (%)		
		Early apoptosis	Late apoptosis	Necrosis
Untreated	88.62	2.98	3.06	5.34
Camptothecin	79.08	14.78	2.30	3.85
2 (1 × IC_50_)	80.48	9.00	4.29	6.23
2 (3 × IC_50_)	72.03	13.37	6.16	8.44
8 (1 × IC_50_)	71.11	10.65	4.92	13.32
8 (3 × IC_50_)	84.00	1.28	0.91	13.81

Because cysteinyl aspartate specific proteinase-3 (caspase-3) plays an important role in the apoptotic pathway, the capacity of madecassic acid (2) and compound 8 to induce caspase 3 activity was measured at 1, 6, and 24 hours post treatment. The results in Table 4 show that compound 2 caused a significant increase in caspase 3 activity in HepG2 cells at 6 h post treatment, and at 3 × GI_50_, with a fold change *vs.* the control of 1.71. Interestingly, compound 8 significantly enhanced caspase 3 activity at 1, 6, and 24 h post treatment with 3 × GI_50_, and at 24 hours post treatment with both 0.3 × and 1 × GI_50_ concentrations.

**Table 4:** The effect of madecassic acid (2) and compound 8 on caspase 3 activity

Compound	Conc.	Fold change of activities					
		1 h		6 h		24 h	
		Mean	SE	Mean	SE	Mean	SE
2	0.3 × GI_50_	0.74	0.02	0.81	0.02	0.99	0.10
	1.0 × GI_50_	0.94	0.10	1.16	0.05	0.68	0.04
	3.0 × GI_50_	0.97	0.02	**1.71** b	0.09	0.54	0.01
8	0.3 × GI_50_	0.59	0.03	0.76	0.04	**1.62** c	0.01
	1.0 × GI_50_	0.76	0.09	1.09	0.06	**1.86** c	0.05
	3.0 × GI_50_	**1.69** a	0.14	**1.27** a	0.13	**1.37** a	0.01
Camptothecin	0.5 μM	1.94	0.05	2.21	0.05	**2.19** c	0.02
Untreated cells		1.00	0.05	1.00	0.03	1.00	0.01

a *P* < 0.05.

b *P* < 0.01.

c *P* < 0.001.

#### 2.2.4. Cell cycle analysis

**Fig. 3:**
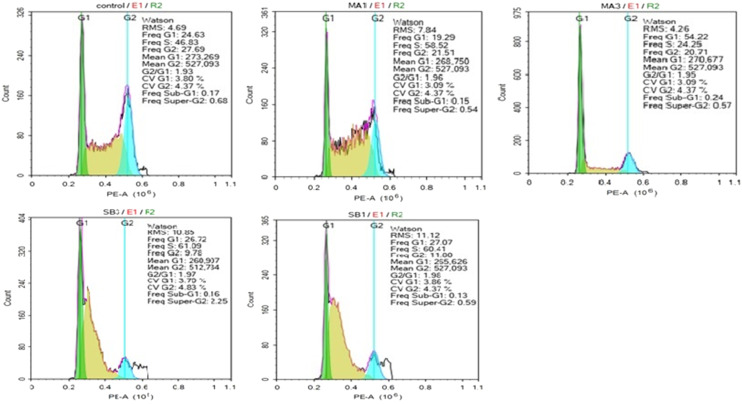
The cell cycle effects of madecassic acid (2) and compound 8. HepG2 cells were incubated with compounds at concentrations of 1 and 3 × GI_50_ for 24 hours followed by PI staining and analysis by flow cytometry. Untreated cells served as a negative control.

According to Pucci and colleagues,^40^ there is an important link between apoptosis and the cell cycle because mitosis and apoptosis express close morphological characteristics, and therefore the effects of madecassic acid (2) and compound 8 on the cell cycle profile of HepG2 cells was investigated using PI staining, 24 hours post compound treatment. With 1 × GI_50_ of madecassic acid (2), there was a reduction in the proportion of cells in G0/G1 and G2/M with a concomitant increase in cells in S phase (Table 5 and Fig. 3). The effects at 3 × GI_50_ of compound 2 were different, with the cell number at both S phase and G2/M reduced and a high number of cells in G0/G1, when compared the vehicle control (0.5% DMSO). In contrast to madecassic acid (2), compound 8 induced cell cycle arrest in S phase with a concomitant decrease in the number of cells in G2/M whilst the number of cells in G0/G1 phase was unchanged compared to the vehicle control at both 1 and 3 × GI_50_ of compound 8.

**Table 5:** Effects of compounds (2 and 8) on the cell cycle

Compound	% G0/G1	% S	% G2/M
Control (DMSO 0.5%)	24.63	46.83	27.69
2 (1 × IC_50_)	19.29	58.52	21.51
2 (3 × IC_50_)	54.22	24.25	20.71
8 (1 × IC_50_)	26.72	61.09	9.78
8 (3 × IC50)	27.07	60.41	11.80

## 3. Conclusion

Fourteen novel conjugates of madecassic acid and silybin have been synthesized through esterification or using amino acids as linkers. The formation of esters at silybin positions 3 or 7 could be controlled depending on the reaction conditions. The new conjugates were evaluated for their cytotoxicity on the HepG2 hepatocellular carcinoma line. Nine compounds showed higher cytotoxic activity than silybin and five compounds exhibited greater cytotoxic activity than madecassic acid itself. Alongside its hepatoprotective activity, silybin has been reported to have cytotoxic activity on HepG2 cells,^41^ a finding supported by our work. Among them, three compounds were very strongly active, and of these, compound 8 was taken forward for further evaluation. Compound 8 demonstrated strong cytotoxic activity against an additional three liver cancer cell lines (HepG3, Huh7 and Huh7R) along with S phase cell cycle arrest and rapid induction of caspase 3 activity in HepG2 cells, which is distinct from madecassic acid (2). These results provide the first evidence of a distinct biological profile of a madecassic acid–silybin conjugate *versus* madecassic acid (2) alone, and could enable the development of treatments that combine cytotoxic effects on liver cancer cells with broader hepatoprotection. The activity of the synthesized hybrid compounds on healthy hepatocytes and other cell types is currently unknown and should be further investigated ideally through *in vivo* testing.

## 4. Experimental section

### 4.1. Chemistry

Chemicals were purchased from Merck and Sigma-Aldrich and used without further purification. Madecassic acid was isolated and purified from *Centella asiatica* cultivated from Hue province by our laboratory. Solvents were redistilled before being used. NMR spectra (^1^H, ^13^C, HMBC, HSQC) were recorded on a Bruker AVANCE 500 MHz with tetramethylsilane (TMS) as the internal standard for 1H and solvent signals for ^13^C NMR. Chemical shifts are reported in parts per million (*δ* ppm). *J* coupling constants in Hz. Proton spectra multiplicities are abbreviated as: MA madecassic acid, SB silybin, s singlet, brs broad single, d doublet, t triplet, q quartet, quin quintet, m multiplet, dd doublet of doublets, dt doublet of triplets, td triplet of doublets. Electrospray ionization (ESI) mass spectra were measured on a 1100 Agilent LC/MS ion trap. Reactions were monitored by thin-layer chromatography using silica gel G60 F254 (Merck). Silica gel 300–400 mesh (Merk) was used for column chromatography.

Hepatocellular carcinoma cell lines (HepG2, Hep3B, Huh and Huh7R) were the kindly gift of Prof. Chi-Ying Huang, Taiwan. DMEM (Gibco, USA) consisted of 10% fetal bovine serum (FBS, Gibco), 2 mM l-glutamine (Gibco, USA) and 1% *anti*–*anti* was used for growth cancer cells. 3-(4,5-Dimethylthiazol-2-yl)-2,5-diphenyltetrazolium bromide (MTT), dimethyl sulfoxide (DMSO), ribonuclease (RNase), and propidium iodide (PI) were original from Sigma Chemical (St. Louis, MO, USA). The eBioessence™ Annexin V Apoptosis Detection Kit was purchased from Invitrogen (Carlsbad, CA, USA). Caspase-3 assay kit (colorimetric) sourced from Abcam (Cambridge, UK).

#### 4.1.1. Synthesis of madecassic acid derivatives

##### 4.1.1.1. 2α,3β,23-Triacetyloxy-6β-hydroxyurs-12-en-28-oic acid (3)

A stirred solution of madecassica cid 2 (1 g, 2.0 mmol) in pyridine (10 mL) was treated with acetic anhydride (600 mg, 6.0 mmol) and stirred for 12 h at room temperature. Reaction mixture was concentrated under reduced pressure and ethyl acetate (200 mL) was added. The solution was washed with 1 N HCl, brine solution, and dried over Na_2_SO_4_. Removal of solvent and purification over SiO_2_ column (*n*-hexane/EtOAc; 2/1) gave 3 (1.60 g, 84%) as a white powder. *R*_f_ = 0.25 (*n*-hexane/EtOAc; 2/1). ESI-MS *m*/*z*: 629.4 [M − H]^−^. ^1^H NMR (500 MHz, CDCl_3_) *δ* 5.28 (t, 1H, *J* = 3.5 Hz, H-12), 5.23 (td, 1H, *J* = 11.0, 4.5 Hz, H-2), 5.01 (d, 1H, *J* = 10.5 Hz, H-3), 4.34 (brs, 1H, H-6), 3.94 (d, 1H, *J* = 12.0 Hz, H-23), 2.12 (d, 1H, *J* = 11.0 Hz, H-18), 2.09 (overlap, 1H, H-11), 2.09 (s, 3H, Ac), 2.06–2.04 (m, 1H, H-1), 2.05 (s, 3H, Ac), 1.98 (s, 3H, Ac), 1.85–1.83 (m, 1H, H-11), 1.75–1.65 (m, 5H), 1.55–1.51 (m, 1H, H-9), 1.49 (s, 3H, H-25), 1.36–1.29 (m, 3H), 1.28 (s, 3H, H-24), 1.14–1.11 (m, 1H, H-1), 1.05 (s, 3H, H-27), 1.05 (s, 3H, H-26), 0.94 (d, 3H, *J* = 6.5 Hz, H-30), 0.86 (d, 3H, *J* = 6.5 Hz, H-29); ^13^C NMR (125 MHz, CDCl_3_) *δ* 183.6 (C-28), 170.8 (C23-OAc), 170.4 (2× OAc), 137.2 (C-13), 125.6 (C-12), 74.9 (C-3), 69.9 (C-2), 67.9 (C-6), 65.4 (C-23), 52.4 (C-18), 48.2 (C-17), 47.9 (C-9), 47.8, 45.8 (C-1), 42.4 (C-4; C-14), 40.7, 39.1, 38.8, 38.6, 37.3, 36.6, 30.6, 27.9, 24.1, 23.5, 23.3 (C-27), 21.1 (C-30), 21.0 (AcO), 21.0 (AcO), 20.8 (AcO), 18.6 (C-25), 18.4 (C-26), 16.9 (C-29), 15.3 (C-24).

##### 4.1.1.2. Synthesis of esters 4 and 5

A stirred solution of acid 3 (50 mg, 0.079 mmol) in dry DCM (5 mL) was added oxalyl chloride (0.2 mL). After being stirred at room temperature for 18 h, the reaction mixture was concentrated under reduced pressure to dryness, which was then treated with silybin (57 mg, 0.119 mmol) and triethyl amine (0.03 mL, 0.237 mmol) in dry DCM (5 mL). Reaction mixture was stirred further overnight and purified over a silica gel column chromatography (DCM/MeOH, 25/1) to obtain esters 4 (30 mg, 40%) and 5 (16 mg, 20%).

##### 7-*O*-(2α,3β,23-Triacetyloxy-6β-hydroxyurs-12-en-28-oyl)silybin (4)

White amorphous powder, *R*_f_ = 0.15 (DCM/MeOH; 25/1), ESI-MS *m*/*z*: 1095.4 [M + H]^+^. ^1^H NMR (500 MHz, CDCl_3_) *δ* 11.05 (s, 1H, OH), 7.18 (dd, 1H, *J* = 1.0 Hz, 9.0 Hz), 7.05–7.01 (m, 2H), 6.95 (s, 1H), 6.93 (overlap, 2H), 6.26–6.23 (m, 1H), 6.21–6.18 (m, 1H), 5.76 (brs, 1H, OH), 5.58–5.55 (m, 1H), 5.48–5.45 (m, 1H), 5.29 (td, 1H, *J* = 11.0, 4.0 Hz, H-2-SB), 5.16–5.12 (m, 1H), 5.04 (d, 1H, *J* = 12.0 Hz, H-3-SB), 4.96 (d, 1H, *J* = 8.5 Hz), 4.58 (d, 1H, *J* = 5.0 Hz), 4.26 (s, 1H, H-6-MA), 4.07–4.05 (m, 1H), 3.92 (overlap, 1H, H-23-SB), 3.92 (s, 3H, OMe-SB), 3.79 (d, 1H, *J* = 12.6 Hz), 3.69 (d, 1H, *J* = 12.0 Hz, H-23-SB), 3.62 (d, 1H, *J* = 12.6 Hz), 3.55 (dd, 1H, *J* = 3.5 Hz, 12.5 Hz), 3.47 (brs, 1H, OH), 2.21 (d, 1H, *J* = 12.0 Hz), 2.11 (overlap, 2H, H-18, H-11-MA), 2.07 (s, 3H, Ac), 2.04 (s, 3H, Ac), 1.98 (s, 3H, Ac), 1.27 (s, 3H, H-25-MA), 1.21 (overlap, 3H, H-24-MA), 1.12 (s, 3H, H-27-MA), 1.04 (s, 3H, H-26-MA), 0.90 (d, 3H, *J* = 6.0 Hz, H-30-MA), 0.80 (d, 3H, *J* = 6.0 Hz, H-29-MA). ^13^C NMR (125 MHz, CDCl_3_) *δ* 197.3 (CO-4-SB), 174.7 (C-28), 171.0 (OAc), 170.8/170.5/170.4 (OAc), 170.3 (OAc), 162.8, 159.9, 146.9, 146.9, 144.3, 143.9, 135.1, 129.1, 127.8, 127.1, 123.8, 122.6, 121.2, 120.8, 117.4/117.4, 116.5/116.2, 114.7, 109.6, 103.9, 103.6, 102.2, 83.1 (C-2-SB), 78.3 (C-10-SB), 76.4 (C-11-SB), 74.8 (C-3-MA), 72.6 (C-3-SB), 69.1 (C-2-MA), 68.2 (C-6-MA), 64.9 (C-23-MA), 61.6 (C-23-SB), 56.1 (OMe-SB), 54.0, 53.4 (C-18-MA), 49.6 (C-17-MA), 49.2 (C-9-MA), 47.6, 46.5/46.3 45.0 (C-1-MA), 44.2, 43.7, 43.3, 42.3 (C-4; C-14), 41.4, 40.9, 38.8, 38.3, 36.6/36.1, 32.2, 30.5, 29.7, 24.0, 23.4 (C-27), 21.1 (C-30), 21.0 (Ac), 20.8 (Ac), 19.1 (C-25-MA), 18.6 (C-26-MA), 17.1/17.0 (C-29-MA), 14.1 (C-24-MA).

##### 7-*O*-(2α,3β,23-Triacetyloxy-6β-hydroxyurs-12-en-28-oyl)hydnocarpin D (5)

White amorphous powder, *R*_f_ = 0.20 (DCM/MeOH; 25/1), ESI-MS *m*/*z*: 1077.2 [M + H]^+^. ^1^H NMR (500 MHz, CDCl_3_) *δ* 11.75 (s, 1H, OH), 7.86–7.82 (2H, overlap), 7.09 (s, 1H), 6.98–6.96 (m, 3H), 6.72 (s, 2H), 6.45 (s, 1H), 5.92 (brs, 1H), 5.59–5.55 (m, 1H), 5.47 (d, 1H, *J* = 12.5 Hz, H-12-MA), 5.30–5.26 (m, 1H), 5.14 (dd, 1H, *J* = 11.0, 4.5 Hz, H-2-SB), 4.99 (brs, 1H), 4.36 (s, 1H, H-6-MA), 4.26 (d, 1H, *J* = 12.0 Hz), 4.13 (brs, 1H), 3.93 (s, 3H, overlap, OMe-SB), 3.86–3.81 (m, 1H), 3.70 (d, 1H, *J* = 12.0 Hz, H-23-SB), 3.60 (d, 1H, *J* = 10.5 Hz), 2.38 (d, 1H, *J* = 11.0 Hz), 2.19–2.13 (m, 2H, overlap, H-18, H-11-MA), 2.08 (s, 3H, Ac), 2.04 (s, 3H, Ac), 1.98 (s, 3H, Ac), 1.47 (s, 3H, H-25-MA), 1.26 (3H, overlap, H-24-MA), 1.25 (s, 3H, H-27-MA), 1.11 (3H, H-26-MA), 0.99 (3H, H-30-MA), 0.90 (d, 3H, *J* = 6.5 Hz, H-29-MA). ^13^C NMR (125 MHz, CDCl_3_) *δ* 175.6 (C-28), 172.9 (CO), 170.9 (OAc), 170.4 (2× OAc), 160.5, 156.6, 155.6, 147.1, 146.6, 145.5, 143.9, 136.9, 127.6, 126.2, 123.9, 121.9, 120.9, 117.3, 117.0, 114.8, 109.6, 104.3, 101.1, 78.7, 76.4, 74.9, 69.9, 67.8, 65.3 (C-23-SB), 61.6, 56.1 (MeO-SB), 52.9, 48.9 (C-17-MA), 48.2 (C-9), 47.8, 45.9 (C-1-MA), 42.8, 42.4 (C-4; C-14-MA), 41.1, 39.2, 38.9, 38.8, 37.3, 36.5, 30.6, 28.0, 24.2, 23.4 (C-27-MA), 21.1 (C-30-MA), 20.9 (Ac), 20.8 (2× Ac), 19.1 (C-25-MA), 18.6 (C-26-MA), 16.9 (C-29-MA), 15.3 (C-24-MA).

##### 4.1.1.3. 3-*O*-(2α,3β,23-Triacetyloxy-6β-hydroxyurs-12-en-28-oyl)-2,3-dehydrosilybin (6)

Acid 3 (50 mg, 0.079 mmol) was treated with DCC (49 mg, 0.237 mmol), silybin (76 mg, 0.158 mmol) and DMAP (10 mg, 0.079 mmol) in dry THF (5 mL) at room temperature for 34 h. Afterward, an amount of oxalic acid (15 mg, 0.12 mmol) was added to destroy the excess of DCC. The reaction mixture was cooled to 0 °C for 30 min, then precipitate was removed by filtration. The filtrate was concentrated under reduced pressure to dryness which was subjected over a silica gel column chromatography eluting with DCM/acetone (5/1) to obtain ester 6 (36 mg, 42%). White amorphous powder, *R*_f_ = 0.2 (DCM/acetone; 5/1), ESI-MS *m*/*z*: 1094.1 [M + H]^+^. ^1^H NMR (600 MHz, CDCl_3_) *δ* 11.75 (brs, 1H, OH), 7.87 (s, 1H), 7.81 (d, 1H, *J* = 8.4 Hz), 7.10 (d, 1H, *J* = 8.4 Hz), 6.98 (s, 2H), 6.96 (1H, overlap), 6.75 (1H, s), 6.48 (s, 1H), 5.40 (brs, 1H, H-12-MA), 5.23 (t, 1H, *J* = 4.5 Hz, H-2-MA), 5.02 (1H, overlap, H-3-MA), 5.06–5.00 (m, 2H), 4.36 (brs, 1H, H-6-MA), 4.16–4.12 (m, 1H), 3.95 (1H, overlap, H-23-SB), 3.94 (s, 3H, OMe-SB), 3.85 (d, 1H, *J* = 12.6 Hz), 3.72 (d, 1H, *J* = 12.0 Hz, H-23-SB), 3.61 (d, 1H, *J* = 12.6 Hz), 2.12 (1H, overlap, H-18-MA), 2.13–2.09 (m, 1H, H-11-MA), 2.07 (s, 3H, Ac), 2.06–2.02 (m, 1H, H-1-MA), 2.05 (s, 3H, Ac), 1.99 (s, 3H, Ac), 1.94–1.86 (m, 10H), 1.47/1.46 (s, 3H, H-25-MA), 1.28 (3H, overlap, H-24-MA), 1.15/1.14 (s, 3H, H-27-MA), 1.11 (s, 3H, H-26), 0.99 (d, 3H, *J* = 6.0 Hz, H-30-MA), 0.90 (d, 3H, *J* = 6.0 Hz, H-29-MA). ^13^C NMR (150 MHz, CDCl_3_) *δ* 175.6 (C-28), 170.9 (OAc), 170.5 (2× OAc), 160.5, 156.7 (C-2-SB), 147.1/146.1, 146.6/145.5, 143.9, 136.9/136.4, 132.5 (C-3-SB), 130.9, 128.8, 127.6, 126.2, 123.9, 121.9, 120.8, 117.3, 117.0, 114.8, 109.6, 106.9, 104.3, 101.1, 78.8 (C-10-SB), 76.4 (C-11-SB), 74.9 (C-3-MA), 69.9 (C-2-MA), 67.9 (C-6-MA), 65.3 (C-23-SB), 56.1 (OMe-SB), 52.9 (C-18-MA), 48.9 (C-17-MA), 48.1 (C-9-MA), 47.8, 45.9 (C-1-MA), 42.9, 42.4 (C-4; C-14-MA), 41.1, 39.2, 38.9, 38.8, 37.3, 36.5, 30.6, 28.0, 24.2, 23.8, 23.4 (C-27-MA), 21.1 (C-30), 20.9 (Ac), 20.8 (Ac), 19.1 (C-25-MA), 18.6 (C-26-MA), 16.9 (C-29-MA), 15.3 (C-24-MA).

##### 4.1.1.4. 3-*O*-(2α,3β,23-Triacetyloxy-6β-hydroxyurs-12-en-28-oyl)-5,7,20,23-*O*-tetraacetyl-2,3-dehydrosilybin (7)

A stirred solution of 6 (15 mg, 0.023 mmol) in pyridine (1 mL) was treated with acetic anhydride (0.1 mL) and stirred further 12 h at room temperature. The reaction mixture was concentrated under reduced pressure and ethyl acetate (20 mL) was added. The organic phase was washed brine solution (2 × 10 mL) and dried over Na_2_SO_4_. Removal of solvent and purification over SiO_2_ column (*n*-hexane–EtOAc; 2/1) gave 7 (14 g, 78%) as a grey foam. *R*_f_ = 0.15 (*n*-hexane/EtOAc; 3/1), ESI-MS *m*/*z*: 1262.1 [M + H]^+^. ^1^H NMR (600 MHz, CDCl_3_) *δ* 7.50 (s, 1H), 7.47–7.44 (m, 1H), 7.25 (1H, overlap), 7.12–7.09 (m, 2H), 6.99 (2H, overlap), 6.77 (s, 1H), 6.48 (s, 1H), 5.41 (brs, 1H, H-12-MA), 5.22 (td, 1H, *J* = 11.4, 5.4 Hz, H-2-MA), 5.01 (2H, overlap, H-3-MA, H-11-SB), 4.41 (d, 1H, *J* = 12.4 Hz), 4.37–4.32 (m, 1H, H-6-MA), 4.35–4.30 (m, 1H), 4.05–4.02 (m, 1H, H-23-SB), 3.95 (1H, overlap, H-23-SB), 3.93 (s, 3H, OMe-SB), 3.72 (d, 1H, *J* = 12.0 Hz, H-23-MA), 2.42 (s, 3H, OAc), 2.35 (1H, overlap), 2.33 (s, 3H, AcO-SB), 2.33 (s, 3H, AcO-SB) 2.32 (s, 3H, AcO-SB), 2.12 (2H, overlap, H-18, H-11-MA), 2.07 (s, 3H, Ac), 2.05 (s, 3H, Ac), 2.03 (s, 3H, Ac), 1.98 (s, 3H, AcO-SB), 1.99 (s, 3H, Ac), 1.94–1.82 (m, 10H), 1.47/1.46 (s, 3H, H-25-MA), 1.28 (3H, overlap, H-24-MA), 1.11 (s, 3H, H-27-MA), 1.10 (s, 3H, H-26-MA), 0.99 (d, 3H, *J* = 6.0 Hz, H-30), 0.88 (d, 3H, *J* = 6.0 Hz, H-29-MA). ^13^C NMR (150 MHz, CDCl_3_) *δ* 174.5 (C-28), 170.9 (OAc), 170.5 (2× OAc), 170.4, 170.2, 169.4, 168.8, 167.9, 156.7 (C-2-SB), 154.8, 154.7, 151.8, 150.3, 145.7, 143.5, 143.5, 140.7, 136.9, 134.1, 133.5 (C-3-SB), 130.9, 128.8, 126.2, 123.4, 119.8, 117.6, 117.4, 114.5, 113.6, 111.1, 108.7, 76.4 (C-10-SB), 75.9 (C-11-SB), 74.9 (C-3-MA), 69.9 (C-2-MA), 68.2, 67.8 (C-6-MA), 65.3 (C-23-SB), 56.1 (OMe-SB), 52.9 (C-18-MA), 49.3 (C-17-MA), 48.0 (C-9), 47.8, 45.9 (C-1), 42.7, 42.4 (C-4; C-14-MA), 41.0, 39.1, 38.9, 38.8, 37.3, 36.4, 30.6, 28.9, 24.2, 23.8, 23.4 (C-27), 21.1 (2× OAc), 20.9 (AcO), 20.8 (AcO), 20.7 (2× OAc), 18.9 (C-25-MA), 18.6 (C-26-MA), 16.9 (C-29-MA), 15.3 (C-24-MA).

#### 4.1.2. General synthesis of madecassic acid conjugated with silybin over linkers

A solution of 2α,3β,23-triacetyloxy-6β-hydroxyurs-12-en-28-oic acid 3 (100 mg, 0.16 mmol) in dry DCM (5 mL) was treated with oxalyl chloride (0.07 mL, 0.8 mmol). After stirring for 14 h at room temperature, the solvent was removed under reduced pressure to dryness and the resulting residue was redissolved in dry DCM (5 mL) followed by addition of amino acid (glycine; or glycine methyl ester or; or β-alanine; or 11-aminoundecanoic acid; or methyl 11-aminoundecanoic acid methyl ester; 1.1 equiv.) in the presence of TEA (0.07 mL, 0.5 mmol). The reaction mixture was stirred for 18 h, and concentrated to dryness which was subsequently exposed to silybin (1.2 equiv.), DCC (1.5 equiv.) and DMAP (1 equiv.) in dry THF at 0 °C. The reaction mixture was stirred at room temperature for 48 h, then an amount of oxalic acid (0.5 equiv.) was added slowly to quenched the excess amount of DCC. The reaction was stirred for 15 min and cooled to −5–0 °C for 1 h. The precipitated solid was removed by filtration. Removal of solvent and purification over a silica gel chromatography column, using DCM/acetone (4/1) provide product 8, 9 and 10.

##### 4.1.2.1. Silybin-3-yl(*N*-(2α,3β,23-triacetyloxy-6β-hydroxyurs-12-en-28-oyl))aminoacetate (8)

Yield 25% (46 mg), white amorphous powder, *R*_f_ = 0.18 (DCM/acetone (4/1), ESI-MS *m*/*z*: 1175.4 [M + Na]^+^. ^1^H NMR (600 MHz, CD_3_OD) *δ* 7.11–7.08 (m, 1H), 7.04 (d, 1H, *J* = 4.8 Hz), 7.05–7.02 (m, 2H), 6.95–6.92 (m, 1H), 6.88–6.85 (m, 1H), 5.98 (s, 1H, H-6-SB), 5.96 (s, 1H, H-8-SB), 5.84 (dd, 1H, *J* = 11.4, 14.4. Hz, H-3-SB), 5.39 (2H, overlap, H-12, H-2-SB), 5.28–5.23 (m, 1H, H-2-MA), 4.97 (2H, overlap, H-10-SB, H-3-MA), 4.29 (brs, 1H, H-6-MA), 4.13–4.09 (m, 1H, H-11-SB), 3.97 and 3.92 (each 1H, overlap, CH_2_-Gly), 3.92 and 3.77 (d, each 1H, *J* = 12.6 Hz, H-23-MA), 3.90 (s, 3H, OMe-SB), 3.73 (d, 1H, *J* = 10.8 Hz, H-23-SB), 3.51 (dd, 1H, *J* = 12.6, 4.8 Hz, H-23-SB), 2.13–2.09 (m, 2H, overlap, H-11, H-18), 2.05 (s, 3H, Ac), 2.04 (s, 3H, Ac), 1.99 (s, 3H, Ac), 1.89–1.85 (m, 1H), 1.76–1.71 (m, 1H, H-11), 1.68–1.63 (m, 4H), 1.48 (s, 3H, H-25), 1.36–1.29 (m, 3H), 1.30 (s, 3H, H-24), 1.12 (s, 3H, H-27), 1.05 (s, 3H, H-26), 0.98 (d, 3H, *J* = 7.2 Hz, H-30), 0.91 (d, 3H, *J* = 7.2 Hz, H-29). ^13^C NMR (150 MHz, CD_3_OD) *δ* 192.2 (C4-SB), 180.3 (C-28-MA), 172.6 (C23-OAc), 172.3 (OAc), 172.2 (OAc), 170.5, 165.5, 164.0, 149.3, 148.4, 145.9, 145.4, 139.1 (C-13-MA), 129.9, 129.4, 127.3 (C-12-MA), 121.8, 118.3, 117.4, 116.3, 112.2, 102.1, 97.8 (C-6-SB), 96.7 (C-8-SB), 82.0 (C-2-SB), 80.1 (C-11-SB), 77.8 (C-3-SB), 76.7 (C-10-SB), 74.5 (C-3-MA), 71.2 (C-2-MA), 67.9 (C-6-MA), 66.3 (C-23-MA), 62.1 (C-23-SB), 56.6 (OMe-SB), 54.3 (C-18), 48.1 (C-17), 47.0 (C-9), 43.7, 41.9 (CH_2_-Gly), 41.2, 40.8, 40.2, 40.1, 38.6, 38.4, 31.9, 28.9, 25.3, 24.4, 24.1, 21.5 (C-30), 20.9 (Ac), 20.7 (2× Ac), 19.1 (C-25), 18.7 (C-26), 17.4 (C-29), 15.7 (C-24).

##### 4.1.2.2. Silybin-3-yl(*N*-(2α,3β,23-triacetyloxy-6β-hydroxyurs-12-en-28-oyl))-3-aminopropanoate (9)

Yield 20% (36 mg), white amorphous powder, *R*_f_ = 0.15 (DCM/acetone (3/1), ESI-MS *m*/*z*: 1167.2 [M + H]^+^. ^1^H NMR (600 MHz, CD_3_OD) *δ* 7.16–7.08 (m, 2H), 7.05–7.01 (m, 2H), 6.93–6.90 (m, 1H), 6.87–6.84 (m, 1H), 5.97/5.95 (s, 1H, H-6-SB), 5.92/5.90 (s, 1H, H-8-SB), 5.86 (dd, 1H, *J* = 12.6 Hz, H-3-SB), 5.36 (2H, overlap, H-12-MA, H-2-SB), 5.27–5.21 (m, 1H, H-2-MA), 4.97 (d, 1H, *J* = 10.2 Hz, H-10-SB), 4.94–4.90 (m, 1H, H-3), 4.29 (brs, 1H, H-6-MA), 4.15–4.11 (m, 1H, H-11-SB), 3.90 and 3.75 (each 1H, overlap, H-23), 3.89/3.87 (s, 3H, OMe-SB), 3.76–3.72 (m, 1H, H-23-SB), 3.58–3.53 (m, 1H, β-CH_2_-Ala), 3.51 (1H, overlap, H-23-SB), 3.47–3.42 (m, 1H, β-CH_2_-Ala), 2.78 (t, 1H, *J* = 6.0 Hz, α-CH_2_-Ala), 2.49–2.44 (m, 1H, α-CH_2_-Ala), 2.15–2.10 (m, 2H, overlap, H-11, H-18), 2.09 (s, 3H, Ac), 2.07 (s, 3H, Ac), 1.99 (s, 3H, Ac), 1.85–1.80 (m, 2H), 1.72–1.65 (m, 4H), 1.44 (s, 3H, H-25), 1.29 (s, 3H, H-24), 1.11 (s, 3H, H-27), 1.07 (s, 3H, H-26), 0.97 (d, 3H, *J* = 7.2 Hz, H-30), 0.91 (d, 3H, *J* = 7.2 Hz, H-29).

##### 4.1.2.3. Silybin-3-yl(*N*-(2α,3β,23-triacetyloxy-6β-hydroxyurs-12-en-28-oyl))-11-amino-undecanoate (10)

Yield 26% (50 mg), white amorphous powder, *R*_f_ = 0.2 (DCM/acetone (4/1), ESI-MS *m*/*z*: 1279.4 [M + H]^+^. ^1^H NMR (500 MHz, CDCl_3_) *δ* 7.07–7.03 (m, 3H), 6.95–6.89 (m, 2H), 5.98 (brs, 1H, H-6-SB), 5.95 (brs, 1H, H-8-SB), 5.72 (dd, 1H, *J* = 12.5 Hz, H-3-SB), 5.32 (brs, 1H, H-12-MA), 5.21 (td, 1H, *J* = 11.0, 4.5 Hz, H-2-MA), 5.05–5.01 (m, 1H, overlap, H-3-MA), 4.98 (1H, overlap, H-2-SB), 4.50–4.46 (m, 1H), 4.26 (brs, 1H, H-6-MA), 4.18 (t, 1H, *J* = 7.0 Hz, H-10), 4.01 (brs, 1H, H-23-MA), 3.88/3.81 (s, 3H, OMe-SB), 3.73 (1H, overlap, H-23-MA), 3.72 (d, 1H, *J* = 12.0 Hz, H-23-SB), 3.58–3.54 (m, 1H, H-23-SB), 3.23–3.19 (m, 2H, overlap, CH_2_-11-AUDA), 2.56 (t, 2H, *J* = 7.0 Hz, CH_2_-11-AUDA), 2.03 (s, 3H, AcO), 2.00 (s, 3H, AcO), 1.95 (s, 3H, OAc), 1.96–1.92 (m, 1H), 1.85–1.81 (m, 2H), 1.74–1.57 (m, 7H), 1.48–1.37 (m, 10H), 1.26–1.12 (m, 26H, CH_2_-11-AUDA), 1.03 (s, 3H, H-27), 1.02 (s, 3H, H-26), 0.92 (s, 3H, H-30), 0.85 (d, 3H, *J* = 6.5 Hz, H-29). ^13^C NMR (125 MHz, CDCl_3_) *δ* 185.6 (C-4-SB), 176.7 (C-28), 172.2 (COO-3-SB), 171.4, 170.9 (OAc), 139.3, 135.2, 134.3 (C-13-MA), 132.5, 131.1, 128.9, 125.6 (C-12-MA), 120.8, 119.9, 116.5, 110.0, 97.2 (C-6-SB), 96.1 (C-8-SB), 83.0 (C-2-SB), 78.5 (C-10-MA), 75.1 (C-11-SB), 73.9 (C-3-MA), 73.2 (C-3-SB), 70.2 (C-2-MA), 67.2 (C-6-MA), 65.3 (C-23-MA), 61.4 (C-23-SB), 56.1 (OMe-SB), 54.2, 53.5 (C-18), 48.1 (C-17), 47.8 (C-9), 45.9 (C-1), 43.0, 42.5 (C-4), 39.9, 39.8 (CH_2_-11-AUDA), 38.8, 37.4, 34.1 (CH_2_-11-AUDA), 29.8–29.1 (8× CH_2_), 28.8, 27.1, 24.8, 23.8, 23.3 (C-27), 21.2 (C-30), 21.1 (Ac), 20.9 (Ac), 20.8 (Ac), 18.6 (C-25), 18.2 (C-26), 17.2 (C-29), 15.3 (C-24).

#### 4.1.3. General acetylation of conjugated madecassic acid derivatives

A stirred solution of ester 8, or 9, or 10 (20 mg) in pyridine (1 mL) was treated with acetic anhydride (0.1 mL) and stirred for 18 h at room temperature. Afterward, the reaction mixture was concentrated under reduced pressure and ethyl acetate (40 mL) was added. The solution was washed brine solution (2 × 10 mL), and dried over Na_2_SO_4_. Removal of solvent and purification over a SiO_2_ column (*n*-hexane/EtOAc; 2/1) yielded product 11, 12 and 13.

##### 4.1.3.1. (5,7,20,23-*O*-Tetraacetyl)silybin-3-yl(*N*-(2α,3β,23-triacetyloxy-6β-hydroxyurs-12-en-28-oyl))aminoacetate (11)

Yield 74% (16 mg), white amorphous powder, *R*_f_ = 0.22 (*n*-hexane/EtOAc; 2/1), ESI-MS *m*/*z*: 1321.3 [M + H]^+^. ^1^H NMR (500 MHz, CDCl_3_) *δ* 7.09–7.06 (m, 2H), 7.04–6.80 (m, 5H), 6.78 (d, 1H, *J* = 2.0 Hz, H-6-SB), 6.60 (d, 1H, *J* = 2.0 Hz, H-8-SB), 5.64 (dd, 1H, *J* = 12.5 Hz, H-3-SB), 5.42 (brs, 1H, H-12-MA), 5.38 (dd, 1H, *J* = 12.5, 3.5 Hz, H-2-SB), 5.23 (td, 1H, *J* = 11.0, 4.5 Hz, H-2-MA), 5.01 (dd, 1H, *J* = 10.5, 3.0 Hz, H-2-SB), 4.96 (d, 1H, *J* = 10.5 Hz, H-3-MA), 4.36 and 3.99 (dd, each 1H, *J* = 13.5, 9.0 Hz, H-23-SB), 4.29 (brs, 1H, H-6), 4.09 and 3.98 (dd, 1H, *J* = 19.0, 5.5 Hz, CH_2_-Gly), 3.97 (1H, overlap, H-23-MA), 3.87/3.86 (s, 3H, OMe-SB), 3.72 (d, 1H, *J* = 12.0 Hz, H-23-MA), 2.35 (s, 3H, AcO-SB), 2.32 (s, 3H, AcO-SB), 2.30 (s, 3H, AcO-SB), 2.14–2.11 (m, 1H, overlap, H-11), 2.06 (s, 3H, Ac), 2.05 (s, 3H, Ac), 2.03 (s, 3H, Ac), 1.96 (s, 3H, AcO-SB), 1.70–1.65 (m, 9H, H-11), 1.48 (s, 3H, H-25), 1.38–1.29 (m, 6H), 1.26 (s, 3H, H-24), 1.05 (s, 3H, H-27), 1.02 (s, 3H, H-26), 0.92 (s, 3H, H-30), 0.87 (d, 3H, *J* = 6.5 Hz, H-29). ^13^C NMR (125 MHz, CDCl_3_) *δ* 184.4 (C4-SB), 177.9 (C-28), 170.8 (C-23-OAc), 170.4 (2× OAc), 170.3 (OAc), 169.1 (COO-3-SB), 168.8 (CO-Gly), 167.8 (2× OAc), 162.5, 156.6, 151.7, 151.5, 144.1, 143.7, 140.6, 134.4 (C-13), 130.8, 128.8, 128.1, 126.3 (C-12), 123.2, 120.7, 119.9, 117.6, 116.4, 111.3, 109.1, 80.8 (C-2-SB), 76.4 (C-10-SB), 75.7 (C-11-SB), 74.9 (C-3-MA), 74.5 (C-3-SB), 69.9 (C-2-MA), 67.6 (C-6-MA), 65.3 (C-23-MA), 62.6 (C-23-SB), 56.1 (OMe-SB), 53.6 (C-18-MA), 48.1 (C-17-MA), 47.9 (C-9-MA), 47.8, 45.9 (C-1-MA), 42.8 (C4-MA), 41.1 (CH_2_-Glycine), 39.7, 38.9, 38.8, 37.3, 36.9, 30.9, 28.9, 27.7, 24.8, 23.8, 23.4 (C-27), 21.2 (C-30), 21.7 (Ac), 20.9 (2× Ac), 20.8 (Ac), 20.7 (2× Ac), 20.6 (2× Ac), 18.6 (C-25), 18.0 (C-26), 17.1 (C-29), 15.3 (C-24).

##### 4.1.3.2. (5,7,20,23-*O*-Tetraacetyl)silybin-3-yl(*N*-(2α,3β,23-triacetyloxy-6β-hydroxyurs-12-en-28-oyl))-3-aminopropanoate (12)

Yield 61% (13 mg), white amorphous powder, *R*_f_ = 0.25 (*n*-hexane/EtOAc; 2/1), ESI-MS *m*/*z*: 1335.1 [M + H]^+^. ^1^H NMR (500 MHz, CDCl_3_) *δ* 7.14–7.09 (m, 2H), 7.05–6.80 (m, 4H, overlap), 6.80 (d, 1H, *J* = 2.0 Hz), 6.60 (d, 1H, *J* = 2.0 Hz), 6.32 (t, 1H, NH), 5.74/5.73 (d, 1H, *J* = 12.5 Hz, H-3-SB), 5.40 (dd, 1H, *J* = 12.5, 3.5 Hz, H-2-SB), 5.36/5.31 (brs, 1H, H-12-MA), 5.21 (td, 1H, *J* = 11.0, 4.5 Hz, H-2-MA), 5.08–5.00 (m, 1H, overlap, H-10-SB), 4.98 (d, 1H, *J* = 10.5 Hz, H-3-MA), 4.36 and 4.03 (dt, each 1H, *J* = 13.5, 9.0 Hz, H-23-SB), 4.29 (1H, overlap, H-6-MA), 4.27 (1H, overlap), 3.92 (dd, 1H, *J* = 12.0, 6.0 Hz, H-23-MA), 3.87/3.86 (s, 3H, OMe-SB), 3.70 (d, 1H, *J* = 12.0 Hz, H-23-MA), 3.59–3.53 (m, 2H, β-CH_2_-Ala), 2.55–2.51 (m, 2H, α-CH_2_- Ala), 2.35 (s, 3H, Ac), 2.32 (s, 3H, Ac), 2.30 (s, 3H, Ac), 2.10 (s, 3H, Ac), 2.04 (s, 3H, Ac), 2.03 (s, 3H, Ac), 2.0 (s, 3H, Ac), 1.97 (s, 3H, Ac), 1.69–1.63 (m, 9H, H-11-MA), 1.45 (s, 3H, H-25-MA), 1.27 (s, 3H, H-24-MA), 1.04 (s, 3H, H-27-MA), 1.03 (s, 3H, H-26-MA), 0.94 (s, 3H, H-30-MA), 0.86 (s, 3H, H-29-MA). ^13^C NMR (125 MHz, CDCl_3_) *δ* 185.9 (C4-SB), 174.5 (C-28), 171.5 (COO-3-SB), 171.2, 170.9 (OAc), 170.4 (OAc), 170.1 (OAc), 168.9, 168.8 (OAc), 167.8 (OAc), 162.5, 156.8, 151.7, 151.4, 144.1, 143.9, 140.6, 134.3 (C-13-MA), 130.8, 128.3, 126.0 (C-12-MA), 123.3, 121.2, 119.8, 117.5/117.4, 116.5/116.4, 111.4/111.3, 109.1, 81.0/80.8 (C-2-SB), 78.3 (C-10-MA), 76.4 (C-3-MA), 75.7 (C-11-MA), 74.9 (C-3-MA), 73.6 (C-3-SB), 69.9 (C-2-MA), 67.7 (C-6-MA), 65.3 (C-23-MA), 62.7/62.6 (C-23-SB), 56.1 (OMe-SB), 53.4 (C-18-MA), 48.1 (C-17-MA), 47.8 (C-9-MA), 47.7, 45.9 (C-1-MA), 42.8 (C-4-MA), 39.7, 38.9, 38.7, 37.3, 35.3 (β-CH_2_- Ala), 34.3 (α-CH_2_- Ala), 30.9, 29.7, 27.7, 24.6, 23.3 (C-27), 21.2 (C-30), 21.2 (Ac), 21.1 (2× Ac), 20.9 (2× Ac), 20.8(2× Ac), 20.7 (Ac), 18.6 (C-25-MA), 18.4 (C-26-MA), 17.1 (C-29-MA), 15.3/15.2 (C-24-MA).

##### 4.1.3.3. (5,7,20,23-*O*-Tetraacetyl)silybin-3-yl(*N*-(2α,3β,23-triacetyloxy-6β-hydroxyurs-12-en-28-oyl))-11-amino-undecanoate (13)

Yield 68% (15 mg), white amorphous powder, *R*_f_ = 0.30 (*n*-hexane/EtOAc; 2/1), ESI-MS *m*/*z*: 1446.8 [M + H]^+^. ^1^H NMR (600 MHz, CDCl_3_) *δ* 7.12 (brs, 1H), 7.11–7.03 (m, 1H), 7.02–6.90 (m, 4H), 6.79–6.74 (m, 1H), 6.59–6.54 (m, 1H), 5.84 (brs, 1H, NH), 5.70/5.68 (td, 1H, *J* = 15 Hz, H-3-SB), 5.38–5.34 (m, 2H, overlap, H-2-SB, H-12-MA), 5.23 (td, 1H, *J* = 13.2, 5.4 Hz, H-2), 5.02 (dd, 1H, *J* = 12.6, 1.8 Hz, H-3-MA), 4.96–4.95 (m, 1H, H-2-SB), 4.37 and 4.00 (dd, each 1H, *J* = 16.2, 10.8 Hz, H-23-SB), 4.36–3.32 (1H, overlap, H-6-MA), 3.94 (d, 1H, *J* = 14.4 Hz, H-23-MA), 3.87/3.86 (s, 3H, OMe-SB), 3.72 (d, 1H, *J* = 14.4 Hz, H-23), 3.27 (quin, 1H, *J* = 6.6 Hz, CH_2_-11-AUDA), 3.06–3.01 (m, 1H, CH_2_-11-AUDA), 2.58 (t, 1H, *J* = 7.8 Hz, CH_2_-11-AUDA), 2.37 (s, 3H, Ac), 2.34 (s, 3H, Ac), 2.27 (s, 3H, Ac), 2.10–2.07 (m, 1H, overlap, H-11), 2.06–2.03 (13H, overlap, 4× Ac), 1.94 (s, 3H, OAc), 1.76–1.64 (m, 9H, H-11-MA), 1.49 (s, 3H, H-25-MA), 1.29–1.21 (m, 28H), 1.08 (s, 3H, H-27-MA), 1.05 (s, 3H, H-26-MA), 0.95 (s, 3H, H-30-MA), 0.88 (d, 3H, *J* = 6.5 Hz, H-29-MA). ^13^C NMR (150 MHz, CDCl_3_) *δ* 185.4 (C4-SB), 177.7 (C-28), 171.9 (COO-3-SB), 171.6, 170.8 (OAc), 170.4 (2× OAc), 169.1 (OAc), 168.8 (OAc), 167.8 (2× OAc), 162.6, 156.4, 151.7, 151.7/151.4, 143.9, 143.6, 140.6, 139.2, 134.3 (C-13), 130.9, 128.8, 125.3 (C-12), 123.3, 121.3, 119.8, 117.3, 116.5, 111.3, 111.0, 108.9, 81.1/81.0 (C-2-SB), 78.3 (C-10-SB), 76.4, 75.6 (C-11-MA), 74.9 (C-3-MA), 73.2 (C-3-SB), 69.9 (C-2-MA), 67.6 (C-6-MA), 65.3 (C-23-MA), 62.7 (C-23-SB), 56.0 (OMe-SB), 54.0, 53.4 (C-18-MA), 48.1 (C-17-MA), 47.8 (C-9), 47.7, 45.9 (C-1-MA), 42.4 (C-4), 40.8, 39.8, 39.5 (CH_2_-11-AUDA), 38.8, 37.3, 34.0 (CH_2_-11-AUDA), 30.9, 29.7–29.1 (8× CH_2_-11-AUDA), 28.9, 27.8, 24.9, 23.8, 23.4 (C-27-MA), 21.2 (C-30), 21.7 (Ac), 21.1 (2× Ac), 20.9 (2× Ac), 20.8 (AcO), 20.7 (Ac), 20.6 (Ac), 18.6 (C-25-MA), 18.3 (C-26-MA), 17.2 (C-29-MA), 15.3 (C-24-MA).

#### 4.1.4. Synthesis of 2α,3β,23-triacetyloxyursa-5,12-dien-28-oic acid (14)

2α,3β,23-Triacetyloxy-6β-hydroxyurs-12-en-28-oic acid 3 (500 mg, 0.79 mmol) in pyridine (4 mL) cooled to 0 °C was added slowly thionyl chloride (0.14 mL, 2 mmol) and stirred for 1 h. The reaction mixture was concentrated to dryness and the residue was added cold water. The solid was collected and purified by a column chromatography (DCM/MeOH; 20/1) on silica gel to obtain 14 as white foam (280 mg, 47%). *R*_f_ = 0.30 (DCM/MeOH; 20/1), ESI-MS *m*/*z*: 612.4 [M]^+^. ^1^H NMR (500 MHz, CDCl_3_) *δ* 5.58–5.54 (m, 1H, H-6), 5.37–5.33 (m, 1H, H-12), 5.32 (td, 1H, *J* = 11.5, 4.0 Hz, H-2), 5.14 (d, 1H, *J* = 10.5 Hz, H-3), 4.25 and 3.68 (d, each 1H, *J* = 12.0 Hz, H-23), 2.37 (dd, 1H, *J* = 19.0, 6.5 Hz, H-7), 2.22 (d, 1H, *J* = 11.5 Hz, H-18), 2.08 (s, 3H, Ac), 2.03 (s, 3H, Ac), 1.98 (s, 3H, Ac), 1.88–1.84 (m, 4H), 1.76–1.65 (m, 5H), 1.64–1.60 (m, 1H, H-7b), 1.55–1.50 (m, 1H, H-1), 1.35–1.28 (m, 5H), 1.2 (s, 3H, H-25), 1.13 (s, 3H, H-24), 0.96 (s, 3H, H-27), 0.93 (s, 3H, H-30), 0.88 (s, 3H, H-26), 0.84 (d, 3H, *J* = 6.5 Hz, H-29); ^13^C NMR (125 MHz, CDCl_3_) *δ* 178.2 (C-28), 171.1 (OAc), 170.5 (OAc), 170.3 (OAc), 144.1 (C-5), 139.0 (C-13), 126.6 (C-12), 122.7 (C-6), 73.7 (C-3), 69.1 (C-2), 64.9 (C-23), 53.2 (C-18), 48.4, 46.2 (C-9), 45.1, 43.5, 42.4 (C-1), 38.8, 38.5, 38.3 (C-8, C-10), 36.5, 32.3 (C-7), 30.6, 27.3, 25.6, 23.9, (C-27), 22.9 (C-25), 22.3 (C-29), 21.9 (C-26), 21.3 (Ac), 21.1 (Ac), 20.8 (Ac), 17.1 (C-24).

#### 4.1.5. General synthesis of dehydrated-madecassic acid conjugated with silybin

A stirred solution of 2α,3β,23-triacetyloxyursa-5,12-dien-28-oic acid 14 (100 mg, 0.163 mmol) in dry DCM (5 mL) was treated with oxalyl chloride (0.07 mL, 0.8 mmol). After stirring for 14 h at room temperature, the solvent was removed under reduced pressure to dryness and the resulting residue was redissolved in dry DCM (5 mL) followed by addition of amino acid (glycine; or glycine methyl ester or; or β-alanine; or 11-aminoundecanoic acid; or methyl 11-aminoundecanoic acid methyl ester; 1.1 equiv.) in the presence of TEA (0.07 mL, 0.5 mmol). The reaction mixture was stirred for 18 h, and concentrated to dryness which was subsequently exposed to silybin (1.2 equiv.), DCC (1.5 equiv.) and DMAP (1 equiv.) in dry THF at 0 °C. The reaction mixture was stirred at room temperature for 48 h, then an amount of oxalic acid (0.5 equiv.) was added slowly to quenched the excess amount of DCC. The reaction was stirred for 15 min and cooled to −5–0 °C for 1 h. The precipitated solid was removed by filtration. Removal of solvent and purification over a silica gel chromatography column, eluting with DCM/acetone (4/1) yielded products 15 and 16.

##### 4.1.5.1. Silybin-3-yl(*N*-(2α,3β,23-triacetyloxyursa-5,12-dien-28-oyl))aminoacetate (15)

Yield 23% (40 mg), white amorphous powder, *R*_f_ = 0.20 (DCM/acetone (4/1), ESI-MS *m*/*z*: 1135.2 [M + H]^+^. ^1^H NMR (500 MHz, CDCl_3_) *δ* 11.34 (s, 1H, OH), 7.11–7.06 (m, 2H), 6.95–6.91 (m, 4H), 6.57 (brs, 1H, NH), 6.06 (brs, 1H, H-8-SB), 5.99 (brs, 1H, H-6-SB), 5.71–6.98 (m, 1H, H-3-SB), 5.56–5.51 (m, 1H, H-6-MA), 5.48–5.43 (m, 1H, H-12-MA), 5.31–5.25 (m, 1H), 5.16–5.12 (m, 1H, H-2-SB), 4.93 (d, 1H, *J* = 10.5 Hz, H-3-MA), 4.22 and 3.69 (d, each 1H, *J* = 12.0 Hz, H-23-MA), 4.10–4.07 and 3.96–3.93 (m, 1H, overlap, CH_2_-Gly), 3.96/3.95 (3H, OMe-SB), 3.79 (1H, overlap, H-23-SB), 3.52 (1H, overlap, H-23-SB), 2.33 (dd, 1H, *J* = 19.0, 6.5 Hz, H-7), 2.18 (d, 1H, *J* = 11.5 Hz, H-18), 2.08 (s, 3H, Ac), 2.06 (s, 3H, Ac), 1.98 (s, 3H, Ac), 1.85–1.80 (m, 4H), 1.76–1.60 (m, 7H), 1.35–1.28 (m, 5H), 1.21 (s, 3H, H-25), 1.15 (s, 3H, H-24), 0.98 (s, 3H, H-27), 0.92 (s, 3H, H-30), 0.89 (s, 3H, H-26), 0.84 (3H, overlap, H-29). ^13^C NMR (125 MHz, CDCl_3_) *δ* 190.2 (C-4-SB), 178.8 (C-28), 171.1 (OAc), 170.8 (OAc), 170.4 (OAc), 168.8 (COO-3-SB), 166.9, 164.3, 162.3, 147.0, 146.5, 144.3 (C-5-MA), 140.1 (C-13-MA), 137.2, 130.9, 128.8, 127.7, 127.1 (C-12-MA), 123.7, 122.6 (C-6-MA), 120.8, 120.5, 117.3, 116.2, 114.8, 109.7, 97.6 (C-6-SB), 96.2 (C-8-SB), 82.9 (C-2-SB), 80.5 (C-10-SB), 78.3 (C-11-SB), 76.3 (C-3-SB), 73.5 (C-3-MA), 69.1 (C-2-MA), 65.0 (C-23-MA), 61.6 (C-23-SB), 56.1 (OMe-SB), 54.7 (C-18-MA), 48.1, 46.6 (C-9-MA), 45.1, 43.5, 42.3 (C-1-MA), 41.4 (CH_2_-Gly), 38.9, 38.8, 38.2 (C-8, C-10-MA), 36.6, 32.3 (C-7-MA), 30.9, 27.0, 23.8 (C-27), 22.9 (C-25), 22.8 (C-29), 21.8 (C-26), 21.1 (Ac), 21.0 (Ac), 20.8 (Ac), 17.3 (C-24-MA), 16.8.

##### 4.1.5.2. Silybin-3-yl(*N*-(2α,3β,23-triacetyloxyursa-5,12-dien-28-oyl))-3-aminopropanoate (16)

Yield 21% (38 mg), white amorphous powder, *R*_f_ = 0.15 (DCM/acetone (4/1), ESI-MS *m*/*z*: 1149.2 [M + H]^+^. ^1^H NMR (500 MHz, CDCl_3_) *δ* 11.42 (s, 1H, OH), 7.09–7.05 (m, 2Hm), 6.97–6.91 (m, 4H), 6.08 (brs, 1H, H-6-SB), 6.03 (brs, 1H, H-8-SB), 5.71 (dt, 1H, *J* = 12.5 Hz, H-3-SB), 5.57–5.52 (m, 1H, H-6-MA), 5.41–5.37 (m, 1H, H-12-MA), 5.29–5.24 (m, 1H, H-2-MA), 5.13 (d, 1H, *J* = 10.5 Hz, H-3-MA), 4.91 (d, 1H, *J* = 6.0 Hz, H-2-SB), 4.23 and 3.68 (d, each 1H, *J* = 12.0 Hz, H-23-MA), 4.09–4.04 (m, 1H, H-10-SB), 3.91 (s, 3H, OMe-SB), 3.84–3.80 and 3.56–3.51 (m, each 1H, H-23-SB), 3.59–3.53 (m, 1H, β-CH_2_-Ala), 2.53–2.48 (m, 1H, α-CH_2_-Ala), 2.36–2.31 (m, 1H, H-7), 2.20–2.15 (m, 1H, H-18), 2.08 (s, 3H, Ac), 2.04 (s, 3H, Ac), 1.99 (s, 3H, Ac), 1.79–1.73 (m, 4H), 1.67–1.60 (m, 5H), 1.35–1.28 (m, 6H), 1.18 (3H, overlap, H-25), 1.12 (s, 3H, H-24), 0.96 (3H, overlap, H-27), 0.92 (s, 3H, H-30), 0.89 (s, 3H, H-26), 0.86 (3H, overlap, H-29). ^13^C NMR (125 MHz, CDCl_3_) *δ* 184.5 (C-4-SB), 178.7 (C-28), 171.3 (OAc), 170.9 (OAc), 170.4 (OAc), 167.2 (COO-3-SB), 164.3, 162.5, 147.0, 146.5, 144.3 (C-5-MA), 144.2, 143.7, 139.9 (C-13-MA), 137.2, 130.9, 128.8, 128.5, 127.7, 126.6 (C-12-MA), 123.1, 122.7 (C-6-MA), 120.8, 120.5, 117.2, 116.4, 114.8, 109.7, 101.4, 97.6 (C-6-SB), 96.3 (C-8-SB), 80.5 (C-2-SB), 78.4 (C-10-SB), 76.4 (C-3-SB), 75.9 (C-11-SB), 73.6 (C-3-MA), 69.4 (C-2-MA), 68.2, 65.0 (C-23-MA), 61.6 (C-23-SB), 56.1 (OMe-SB), 53.8 (C-18), 48.1, 46.7 (C-9-MA), 45.1, 43.5, 42.3 (C-1-MA), 38.9, 38.8, 38.5 (C-8, C-10-MA), 36.9, 35.3 (β-CH_2_-Ala), 33.9 (α-CH_2_- Ala), 32.5 (C-7), 30.9, 27.0, 23.8, 22.9 (C-25-MA), 22.8 (C-29), 21.8 (C-26-MA), 20.9 (Ac), 20.8 (Ac), 20.6 (Ac), 17.3 (C-24), 14.0.

#### 4.1.6. General acetylation of conjugated compounds 15 and 16

A stirred solution of 15 or 16 (15 mg) in pyridine (1 mL) was treated with acetic anhydride (0.1 mL) and stirred for 14 h at room temperature. The reaction mixture was concentrated under reduced pressure and ethyl acetate (20 mL) was added. The solution was washed brine solution (2×10 mL), and dried over Na_2_SO_4_. Removal of solvent and purification over a SiO_2_ column chromatography (*n*-hexane/EtOAc; 2/1) yielded product 17 and 18.

##### 4.1.6.1. (5,7,20,23-*O*-Tetraacetyl)silybin-3-yl(*N*-(2α,3β,23-triacetyloxyursa-5,12-dien-28-oyl))aminoacetate (17)

Yield 61% (10 mg), white amorphous powder, *R*_f_ = 0.25 (*n*-hexane/EtOAc; 2/1), ESI-MS *m*/*z*: 1303.4 [M + H]^+^. ^1^H NMR (600 MHz, CDCl_3_) *δ* 7.11–7.08 (m, 2H), 7.02–6.96 (m, 4H), 6.79 (d, 1H, *J* = 1.2 Hz, H-8-SB), 6.59 (d, 1H, *J* = 1.2 Hz, H-6-SB), 6.46 (brs, 1H, NH), 5.66 (td, 1H, *J* = 12.6 Hz, H-3-SB), 5.58–5.52 (m, 1H, H-6-MA), 5.50–5.46 (m, 1H, H-12-MA), 5.41–5.36 (m, 1H, H-2-SB), 5.30–5.25 (m, 1H, H-2-MA), 5.13 (d, 1H, *J* = 11.4 Hz, H-3-MA), 4.96 (d, 1H, *J* = 8.4 Hz, H-11-SB), 4.37 (dt, 1H, *J* = 10.8, 2.4 Hz, CH_2_-23-SB), 4.29–4.24 (m, 1H, H-10-SB), 4.22–4.20 (m, 1H, H-23-MA), 4.14–4.09 (m, 1H, CH_2_-Gly), 3.99 (dd, 1H, *J* = 16.2, 10.8 Hz, H-23-SB), 3.95–3.90 (m, 1H, CH_2_-Gly), 3.86 (s, 3H, OMe-SB), 3.72 (d, 1H, *J* = 12.0 Hz, H-23-MA), 2.37 (s, 3H, Ac), 2.32 (s, 3H, Ac), 2.29 (s, 3H, Ac), 2.08 (s, 3H, Ac), 2.06 (s, 3H, Ac), 2.04 (s, 3H, Ac), 1.98 (s, 3H, Ac), 1.78–1.63 (m, 11H), 1.35–1.28 (m, 5H), 1.22 (s, 3H, H-25-MA), 1.12 (s, 3H, H-24-MA), 0.98 (s, 3H, H-27-MA), 0.94 (s, 3H, H-30), 0.88 (s, 3H, H-26-MA), 0.85 (3H, overlap, H-29-MA). ^13^C NMR (150 MHz, CDCl_3_) *δ* 184.5 (C-4-SB), 178.0 (C-28), 171.0 (OAc), 170.8 (OAc), 170.4 (OAc), 170.3, 169.1, 169.0 (COO-3-SB), 168.7, 167.8, 162.3, 156.6, 151.7, 151.5, 144.2 (C-5-MA), 143.8, 143.6, 140.6, 140.2 (C-13-MA), 135.1, 134.3, 132.5, 130.9, 128.8, 127.9, 126.9 (C-12-MA), 123.7, 123.3, 122.6 (C-6-MA), 120.9, 120.7, 119.9, 117.6, 116.4, 111.4, 111.2, 110.4, 109.1, 80.8 (C-2-SB), 77.8 (C-10-SB), 76.4 (C-11-SB), 75.7 (C-3-MA), 73.5 (C-3-SB), 70.1, 69.1 (C-2-MA), 68.2, 64.9 (C-23-MA), 62.6 (C-23-SB), 56.1 (OMe-SB), 53.9 (C-18-MA), 48.3, 46.0 (C-9-MA), 45.1, 43.7, 42.4 (C-1-MA), 41.0 (CH_2_-Gly), 38.9, 38.8, 38.8 (C-8, C-10-MA), 38.4, 36.6, 32.3 (C-7-MA), 30.8, 30.6, 30.4, 28.9, 27.6, 23.8, 22.9 (C-25-MA), 22.8 (C-29), 22.4, 21.2 (Ac), 21.1 (OAc), 20.9 (Ac), 20.8 (2× Ac), 20.7 (Ac), 20.6 (Ac), 17.3 (C-24-MA), 16.8.

##### 4.1.6.2. (5,7,20,23-*O*-Tetraacetyl)silybin-3-yl(*N*-(2α,3β,23-triacetyloxyursa-5,12-dien-28-oyl))-3-amino-propanoate (18)

Yield 54% (9 mg), white amorphous powder, *R*_f_ = 0.18 (*n*-hexane/EtOAc; 2/1), ESI-MS *m*/*z*: 1317.3 [M + H]^+^. ^1^H NMR (600 MHz, CDCl_3_) *δ* 7.12–7.08 (m, 2H), 7.00–6.94 (m, 4H), 6.80 (s, 1H, H-8-SB), 6.41 (s, 1H, H-6-SB), 5.74 (dd, 1H, *J* = 12.6 Hz, H-3-SB), 5.57–5.55 (m, 1H, H-6-MA), 5.41–5.38 (m, 1H, H-12-MA), 5.34–5.32 (m, 2H), 5.15–5.11 (m, 1H, H-3-MA), 4.93 (d, 1H, *J* = 12.6 Hz, H-2-SB), 4.36 and 4.00 (d, each 1H, *J* = 7.5 Hz, H-23-SB), 4.26–4.23 and 3.75–3.71 (m, each 1H, overlap, H-23-MA), 4.22 (1H, overlap, H-10-SB), 3.86 (s, 3H, OMe-SB), 3.45–3.42 and 3.36–3.31 (m, each 1H, β-CH_2_-Ala), 2.47 (2H, overlap, α-CH_2_-Ala), 2.37 (s, 3H, Ac), 2.32 (s, 3H, Ac), 2.29 (s, 3H, Ac), 2.08 (s, 3H, Ac), 2.03 (s, 3H, Ac), 2.0 (s, 3H, Ac), 1.97 (s, 3H, Ac), 1.95–1.90 (m, 5H), 1.76–1.61 (m, 15H), 1.25 (3H, overlap, H-25-MA), 1.12 (s, 3H, H-24-MA), 0.96 (3H, overlap, H-27-MA), 0.92 (s, 3H, H-30-MA), 0.89 (3H, overlap, H-26-MA), 0.88 (3H, overlap, H-29-MA). ^13^C NMR (150 MHz, CDCl_3_) *δ* 184.5 (C-4-SB), 178.6 (C-28), 171.1 (OAc), 170.4 (2× OAc), 168.7, 167.8 (COO-3-SB), 162.6, 157.1/156.6, 151.7/151.4, 143.9, 143.5 (C-5-MA), 140.6 (C-13-MA), 134.3, 132.5, 130.9, 128.8, 126.5 (C-12-MA), 123.6, 123.3, 122.7 (C-6-MA), 119.9, 111.2, 109.1, 81.2 (C-2-SB), 78.5 (C-10-SB), 76.5 (C-11-SB), 75.6 (C-3-MA), 73.6 (C-3-SB), 69.1 (C-2-MA), 64.9 (C-23-MA), 62.6 (C-23-SB), 56.1 (OMe-SB), 49.3, 47.9 (C-9-MA), 46.5, 45.1, 43.7, 42.4 (C-1-MA), 40.9, 38.8, 38.5, 38.2 (C-8, C-10-MA), 37.1, 36.6 (β-CH_2_-Ala), 34.9 (α-CH_2_-Ala), 32.3 (C-7), 30.9, 30.4, 29.4, 29.1, 28.9, 23.8, 23.5, 22.9 (C-25-MA), 22.7 (C-29-MA), 22.3 (C-26-MA), 21.2 (OAc), 21.1 (Ac), 20.9 (2× Ac), 20.8 (2× Ac), 20.7 (OAc), 17.3 (C-24), 14.0.

### 4.2. Growth inhibition assay

Liver ancer cell lines HepG2, Hep3B, Huh7 and Huh7R were grown in DMEM containing 10% FBS and 1% antibiotic (*anti*–*anti*, Gibco, ThermoFisher Scienctific) in a humidified atmosphere at 37 °C and 5% CO_2_. In order to test the cytotoxic effect of compounds on different cell lines, cells were detached from the adherent culture surface by trypsin–EDTA (0.05%) and then seeded into 96 wells-plate at the density of 3 × 10^4^ cell per mL and treated with an eight titration of each compound. Ellipticine was used as a reference (positive) control. After 96 hours of incubation, all cell culture media was removed from wells followed by addition of 100 μL MTT solution (0.5 mg mL^−1^ solute in fresh medium) per well and incubation at 37 °C for 4 hours. The MTT solution was then removed and DMSO (200 μL) to each well to solubilise the formazan product and mix each sample again using a pipette before determining the absorbance at 570 nm. GI_50_ values (compound concentration that reduces the MTT assay value by 50% *versus* untreated control) were calculated using GraphPad Prism 5.0 software.

### 4.3. Cell cycle determination

To evaluate cell cycle effects, HepG2 cells were seeded in T25 flasks at a density of 1 × 10^5^ cell per mL at 37 °C in 5% CO_2_ and treated with compound at concentrations of 1 × IC_50_ and 3 × IC_50_ from the result of the cytotoxic assay. After 24 hours of treatment, cells were gently harvested with trypsin and then washed with PBS (pH 7,4). In the next step, 70% ethanol was slowly added to the cells after which they were kept in a fridge (4 °C) for at least 2 h to fix the cells. After fixation the cells were pelleted by centrifugation and the ethanol completely removed resuspension with RNase A (1 mg mL^−1^) and incubation at 37 °C for 15 min, followed by PI staining for 45 min. Finally, at least 10 000 cells were analysed using a NovoCyte flow cytometer system with NovoExpress software (ACEA Bioscience Inc.) to generate cell cycle profiles.

### 4.4. Caspase 3 inducible assay

A Caspase-3 Colorimetric Assay Kit (Biovision Inc.) was used for establishing the caspase 3 activity of tested samples. Treated cells were lysed in lysis buffer for 10 minutes in ice and centrifuged at 10 000 × *g* for 1 minute to remove residual cellular debris (cell pellet). After determination of the protein concentration of each sample (Bradford assay), 80 μg of each sample in 50 μL assay buffer was added to 50 μL DTT (10 mM) and 5 μL of DEVD-pNA (200 μM) in each well of a 96-well plate in triplicate. The plate was incubated at 37 °C for 1 hour. The absorbance was read at 405 nm using a microplate reader (BioTek, ELx800).

### 4.5. Detection of apoptotic inducible activities by flow cytometry

The eBioessence™ Annexin V Apoptosis Detection Kit was used to measure the percentage of apoptotic cells after treatment with each compound for 24 hours according to the manufacturer's instructions. Briefly, after harvesting by trypsin–EDTA, cells were washed with cold PBS to completely remove trace medium. Cell pellets were resuspended in binding buffer and then stained with annexin V-FITC for 15 minutes at room temperature (protected from light) before being washed with the binding buffer to remove the unstained dye, resuspension in 190 μL of binding buffer and addition of 10 μL of PI solution (20 μg mL^−1^) in a binding buffer. Analysis was performed using a NovoCyte flow cytometer system and NovoExpress software (ACEA Bioscience Inc.) to identify the proportion of apoptotic cells.

### 4.6. Statistical analysis

Results were analysed using Excel and GraphPad Prism 5.0 software and reported as mean ± standard error (SE). GraphPad Prism 5 software using unpaired *t*-test and one-way analysis of variance was used for data analysis. A value of *P* < 0.05 was considered to indicate statistical significance.

## Data availability

Processed data for this paper is included in the main manuscript. Raw data for this paper (molecular characterisation, assay readout) is available from the authors upon request.

## Conflicts of interest

There is no conflict of interest to declare.

## Supplementary Material

MD-OLF-D4MD00170B-s001
