# Supplementary material for: Synthesis and cytotoxic activity of madecassic acid–silybin conjugate compounds in liver cancer cells
Source: RSC Med Chem. 2024 Aug 2;15(10):3418–32. doi: 10.1039/d4md00170b (PMC11343037; doi:10.1039/d4md00170b)
Supplement: MD-015-D4MD00170B-s001 [file MD-015-D4MD00170B-s001.pdf]

Supplementary Information (SI) for RSC Medicinal Chemistry.
This journal is © The Royal Society of Chemistry 2024

SUPPORTING INFORMATION

SYNTHESIS AND CYTOTOXIC ACTIVITY OF MADECASSIC ACID – SILYBIN CONJUGATE COMPOUNDS IN LIVER CANCER CELLS

Tran Van Chien [a,b], Tran Thi Phuong Thao [a,b], Nguyen The Anh [a], Tran Van Loc [a], Pham Thi Ninh [a], Nguyen Thi Luu [a], Nguyen Thi Dung [a], Michelle D. Garrett [c], Nguyen Thi Nga [d], Do Thi Thao [d], Christopher J. Serpell [e,*], Tran Van Sung [a,*]

[a] Institute of Chemistry, Vietnam Academy of Science and Technology, 18 Hoang Quoc Viet Road, Cau Giay, Hanoi, Vietnam

[b] Graduate University of Science and Technology, Vietnam Academy of Science and Technology, 18 Hoang Quoc Viet Road, Cau Giay, Hanoi, Vietnam

[c] School of Biosciences, Stacey Building, University of Kent, Canterbury, Kent CT2 7NJ, UK

[d] Institute of Biotechnology, Vietnam Academy of Science and Technology, 18 Hoang Quoc Viet Road, Cau Giay, Hanoi, Vietnam

[e] Department of Pharmaceutical and Biological Chemistry, School of Pharmacy, University College London, 29-39 Brunswick Square, London, WC1N 1AX, UK

Table of Contents

Figure S1. 1H and 13C NMR ($CDCl_3$, 500 MHz) spectra of compound **3**....1
Figure S2. ESI-MS spectrum of compound **3**....2
Figure S3. 1H and 13C NMR ($CDCl_3$, 500 MHz) spectra of compound **4**....3
Figure S4. ESI-MS spectrum of compound **4**....3
Figure S5. 1H and 13C NMR ($CDCl_3$, 500 MHz) spectra of compound **5**....4
Figure S6. ESI-MS spectrum of compound **5**....5
Figure S7. 1H and 13C NMR ($CDCl_3$, 600 MHz) spectra of compound **6**....6
Figure S8. ESI-MS spectrum of compound **6**....6
Figure S9. 1H and 13C NMR ($CDCl_3$, 600 MHz) spectra of compound **7**....7
Figure S10. ESI-MS spectrum of compound **7**....8
Figure S11. 1H and 13C NMR (CD_3OD, 600 MHz) spectra of compound **8**....9
Figure S12. ESI-MS spectrum of compound **8**....9
Figure S13. HPLC analysis of compound **8**....10
Figure S14. 1H NMR (CD_3OD, 600 MHz) spectrum of compound **9**....10
Figure S15. ESI-MS spectrum of compound **9**....11
Figure S16. HPLC analysis of compound **9**....11
Figure S17. 1H and 13C NMR ($CDCl_3$, 500 MHz) spectra of compound **10**....12
Figure S18. ESI-MS spectrum of compound **10**....13
Figure S19. HPLC analysis of compound **10**....13
Figure S20. 1H and 13C NMR ($CDCl_3$, 500 MHz) spectra of compound **11**....14
Figure S21. ESI-MS spectrum of compound **11**....15
Figure S22. 1H and 13C NMR ($CDCl_3$, 500 MHz) spectra of compound **12**....16
Figure S23. ESI-MS spectrum of compound **12**....16
Figure S24. 1H and 13C NMR ($CDCl_3$, 500 MHz) spectra of compound **13**....17
Figure S25. ESI-MS spectrum of compound **13**....18
Figure S26. 1H and 13C NMR ($CDCl_3$, 500 MHz) spectra of compound **14**....19
Figure S27. ESI-MS spectrum of compound **14**....19
Figure S28. 1H and 13C NMR ($CDCl_3$, 500 MHz) spectra of compound **15**....20
Figure S29. ESI-MS spectrum of compound **15**....21
Figure S30. HPLC analysis of compound **15**....21
Figure S31. 1H and 13C NMR ($CDCl_3$, 500 MHz) spectra of compound **16**....22
Figure S32. ESI-MS spectrum of compound **16**....23
Figure S33. HPLC analysis of compound **16**....23
Figure S 34. 1H and 13C NMR ($CDCl_3$, 500 MHz) spectra of compound **17**....24
Figure S35. ESI-MS spectrum of compound **17**....25
Figure S36. 1H and 13C NMR ($CDCl_3$, 500 MHz) spectra of compound **18**....26
Figure S37. ESI-MS spectrum of compound **18**....26

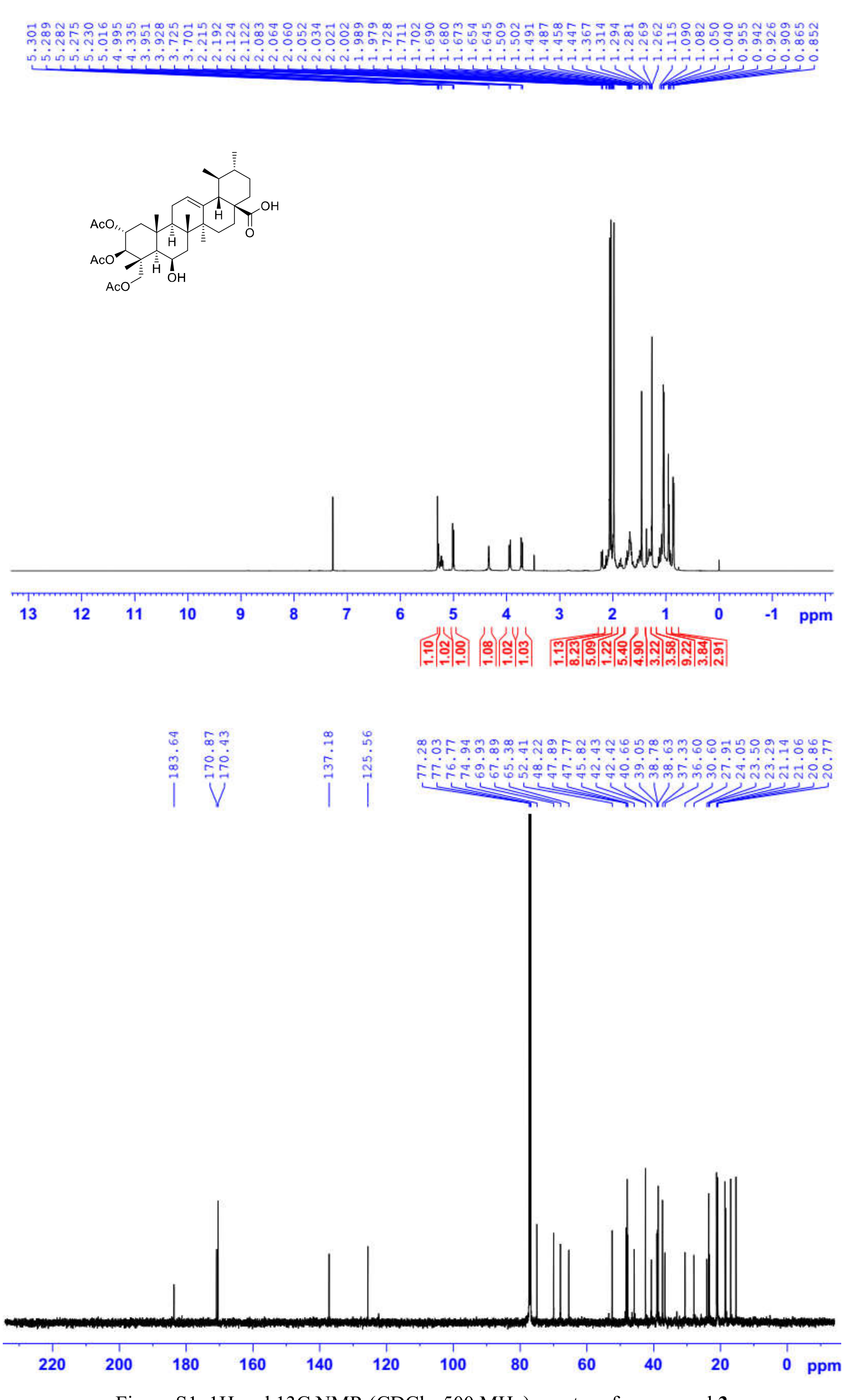

Figure S1. 1H and 13C NMR ($CDCl_3$, 500 MHz) spectra of compound **3**

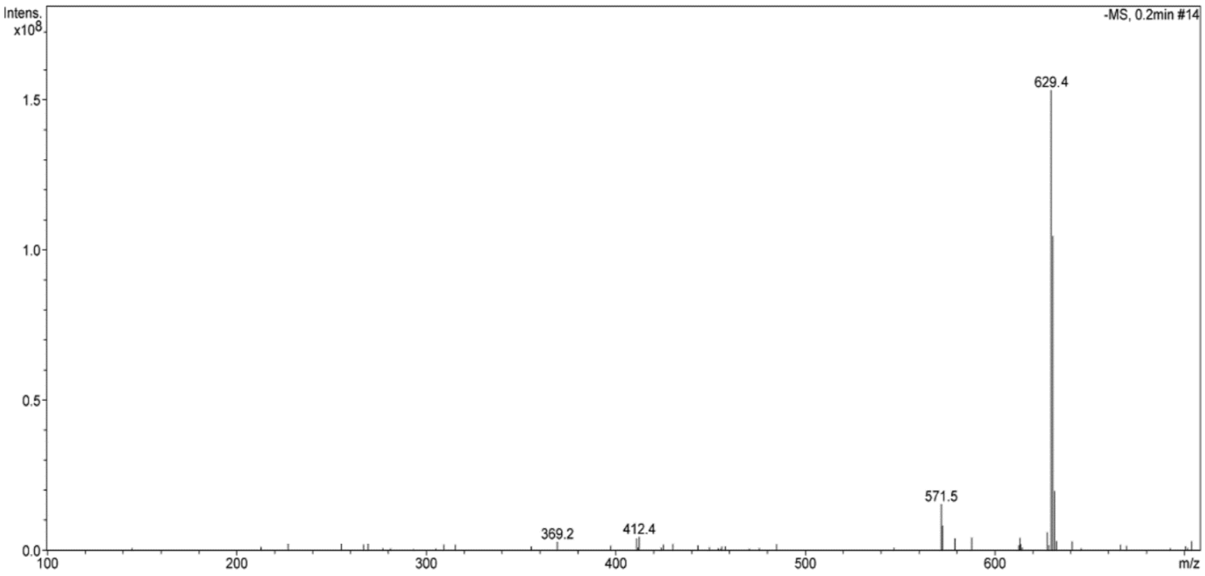

Figure S2. ESI-MS spectrum of compound **3**

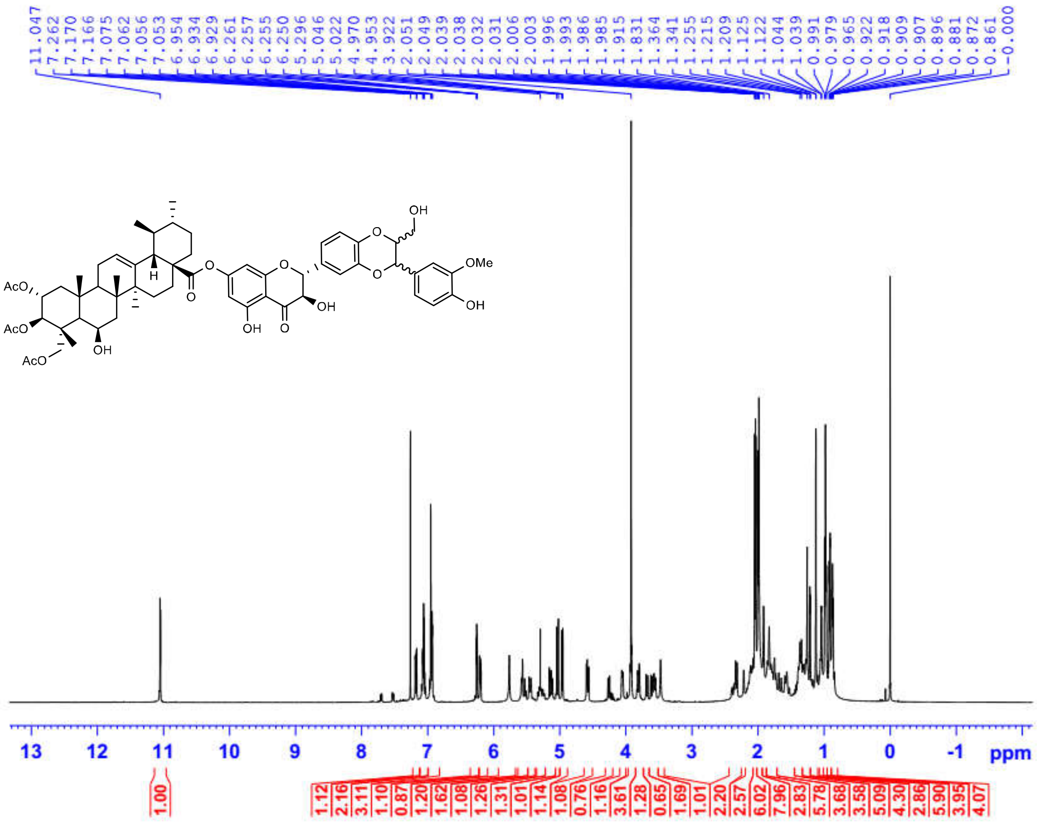

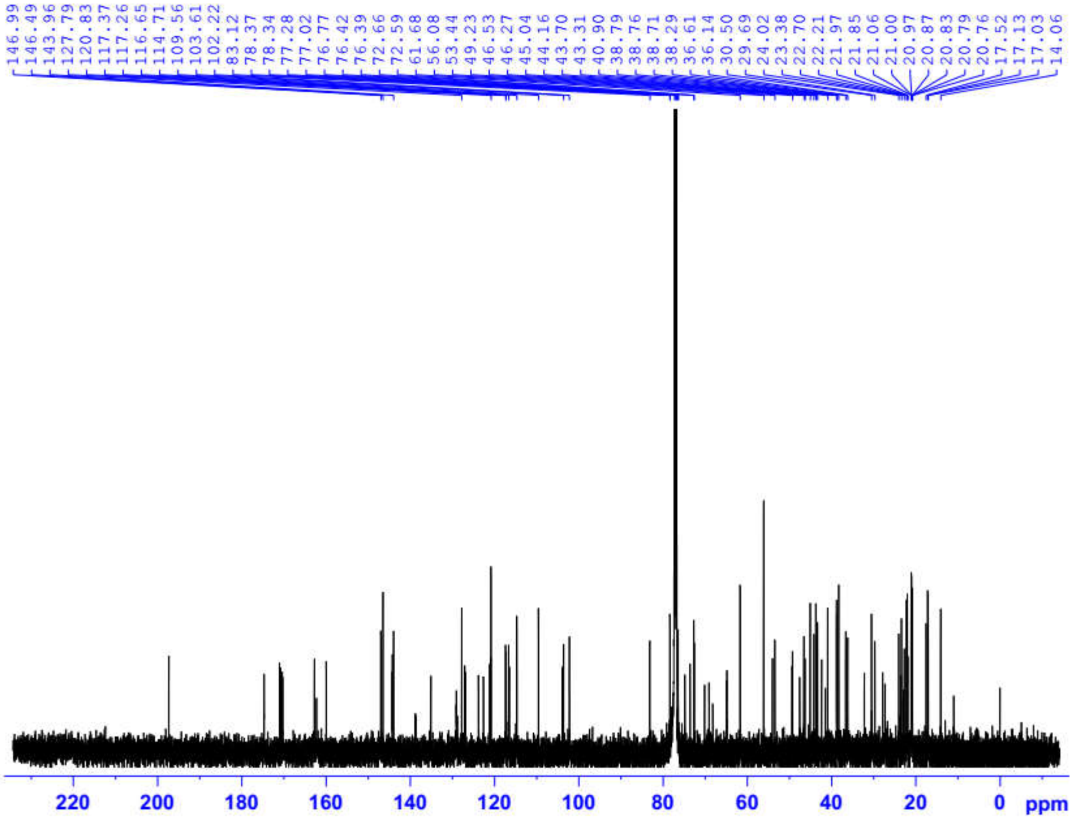

Figure S3. 1H and 13C NMR ($CDCl_3$, 500 MHz) spectra of compound **4**

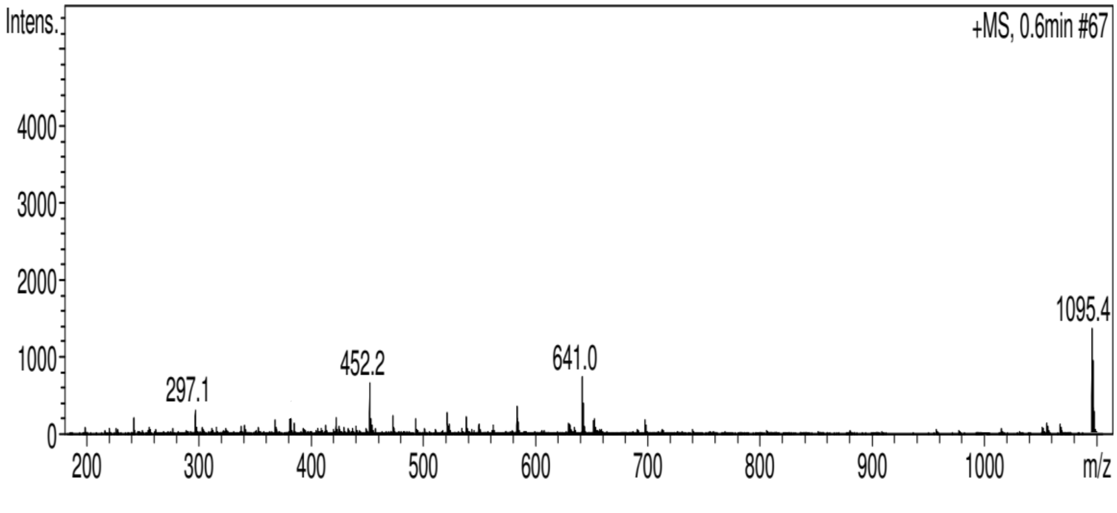

Figure S4. ESI-MS spectrum of compound **4**

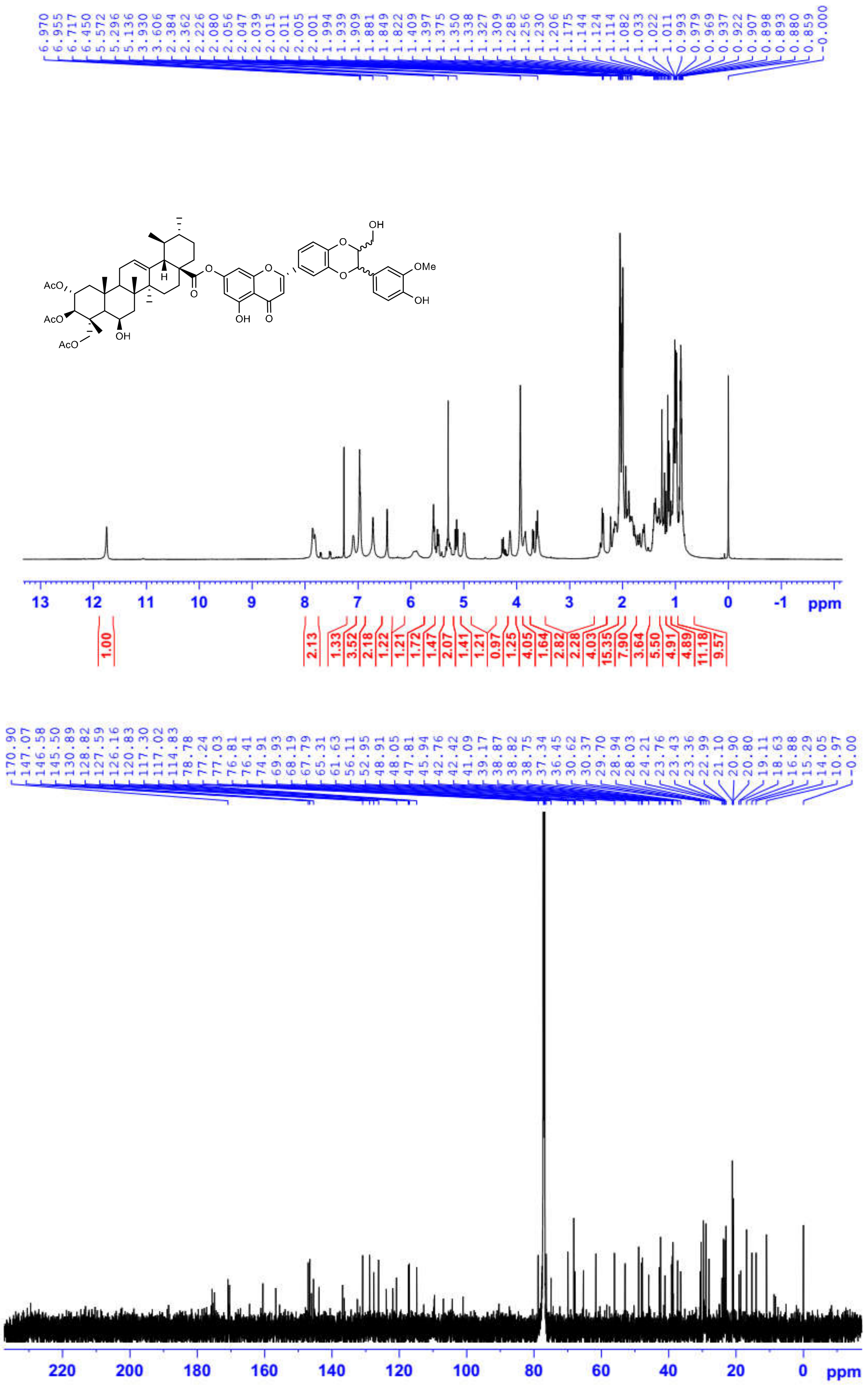

Figure S5. 1H and 13C NMR ($CDCl_3$, 500 MHz) spectra of compound **5**

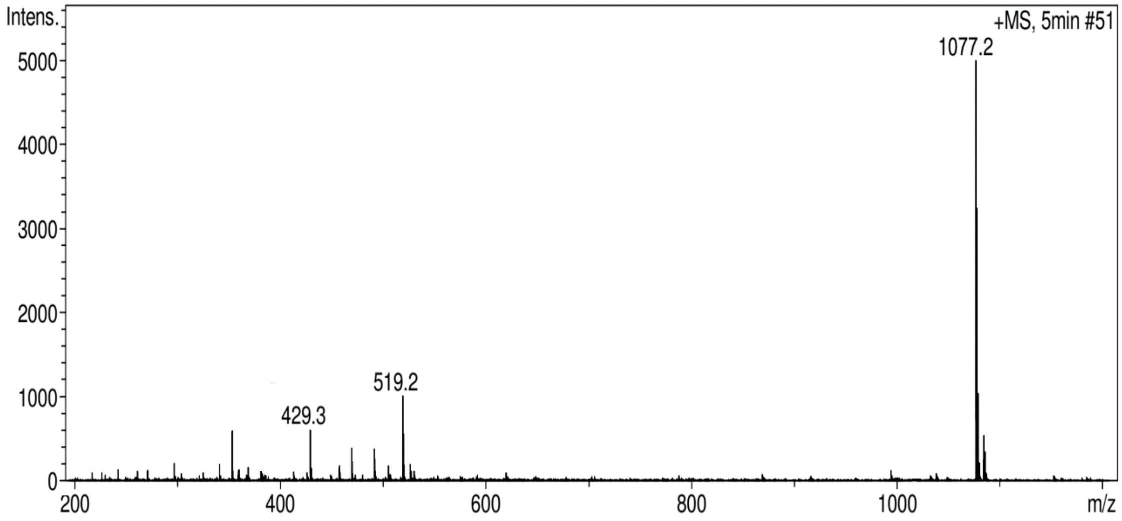

Figure S6. ESI-MS spectrum of compound **5**

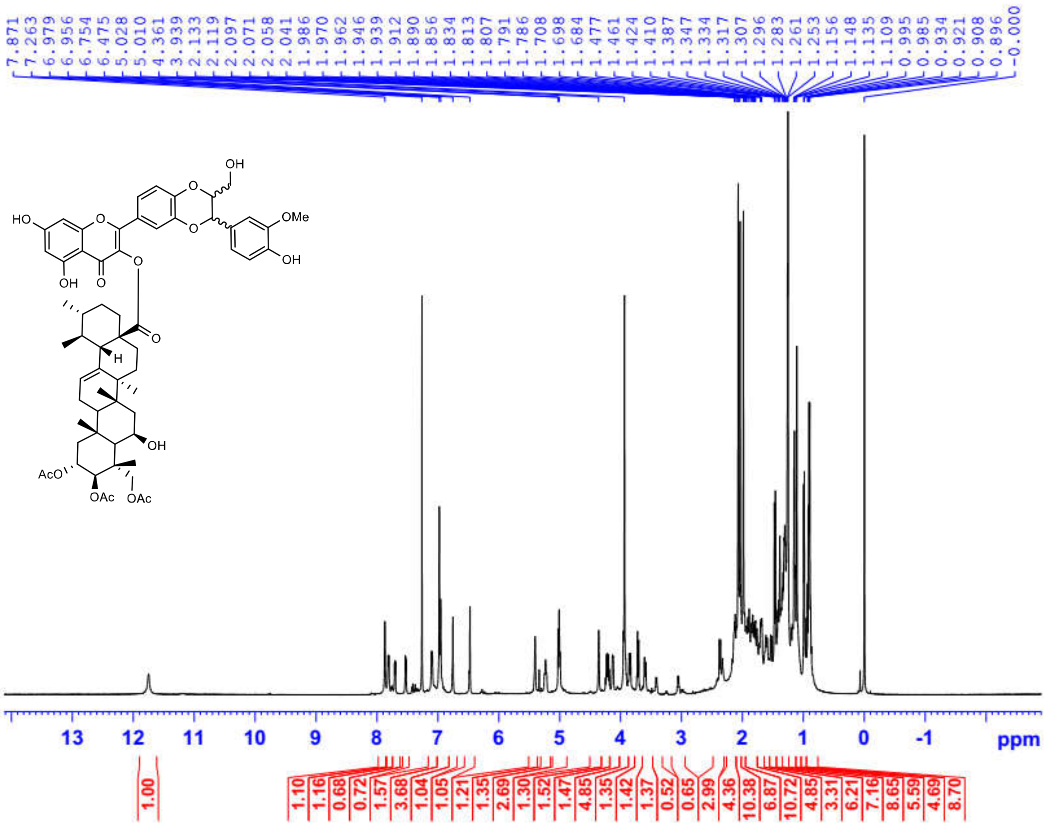

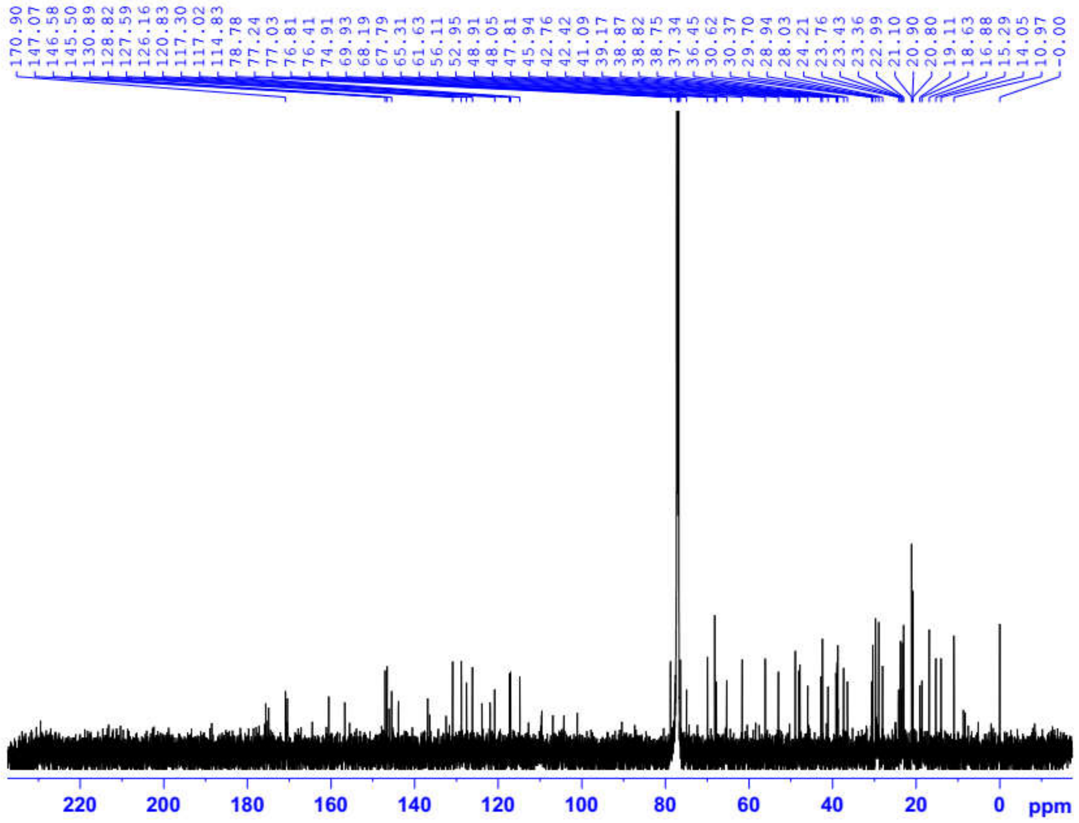

Figure S7. 1H and 13C NMR ($CDCl_3$, 600 MHz) spectra of compound **6**

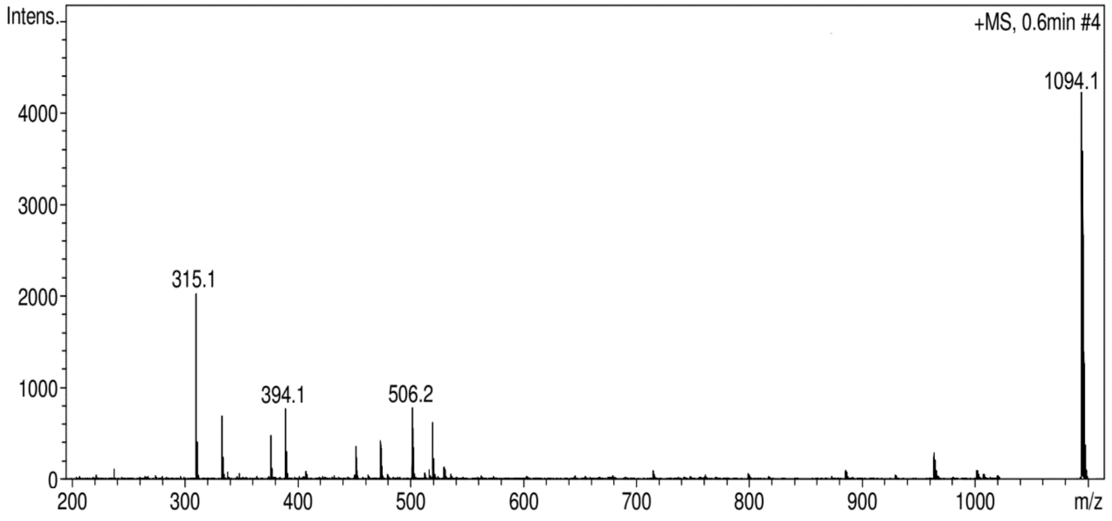

Figure S8. ESI-MS spectrum of compound **6**

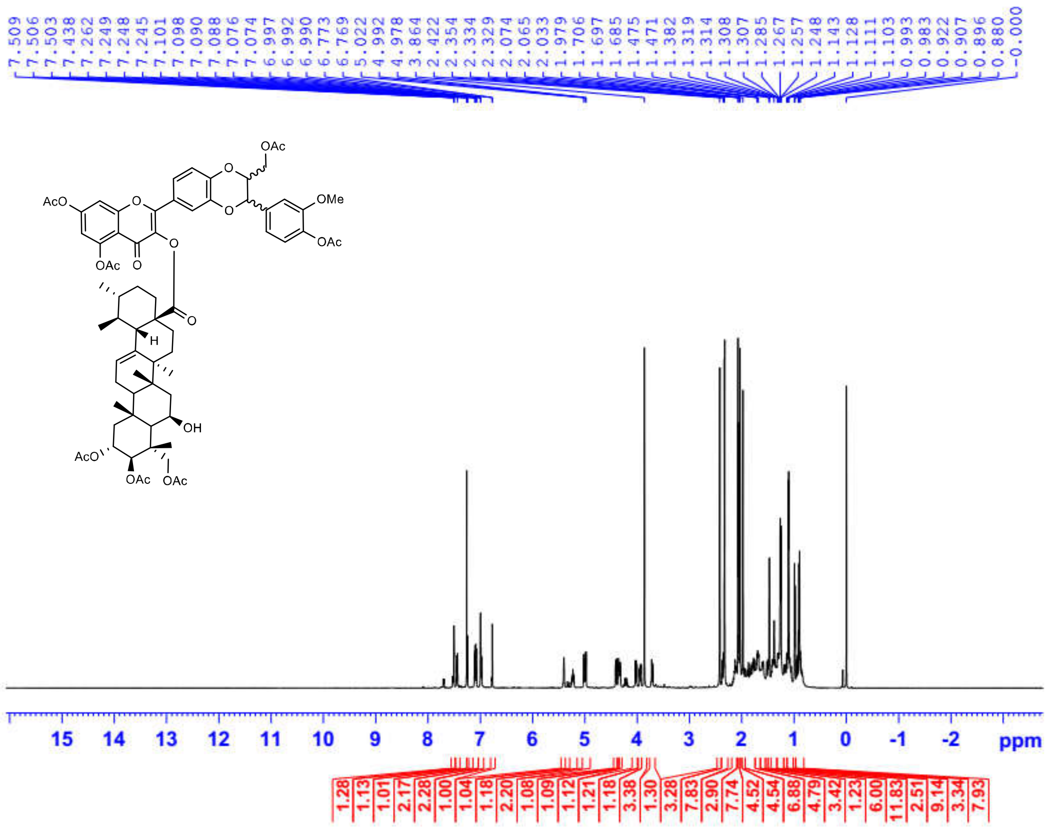

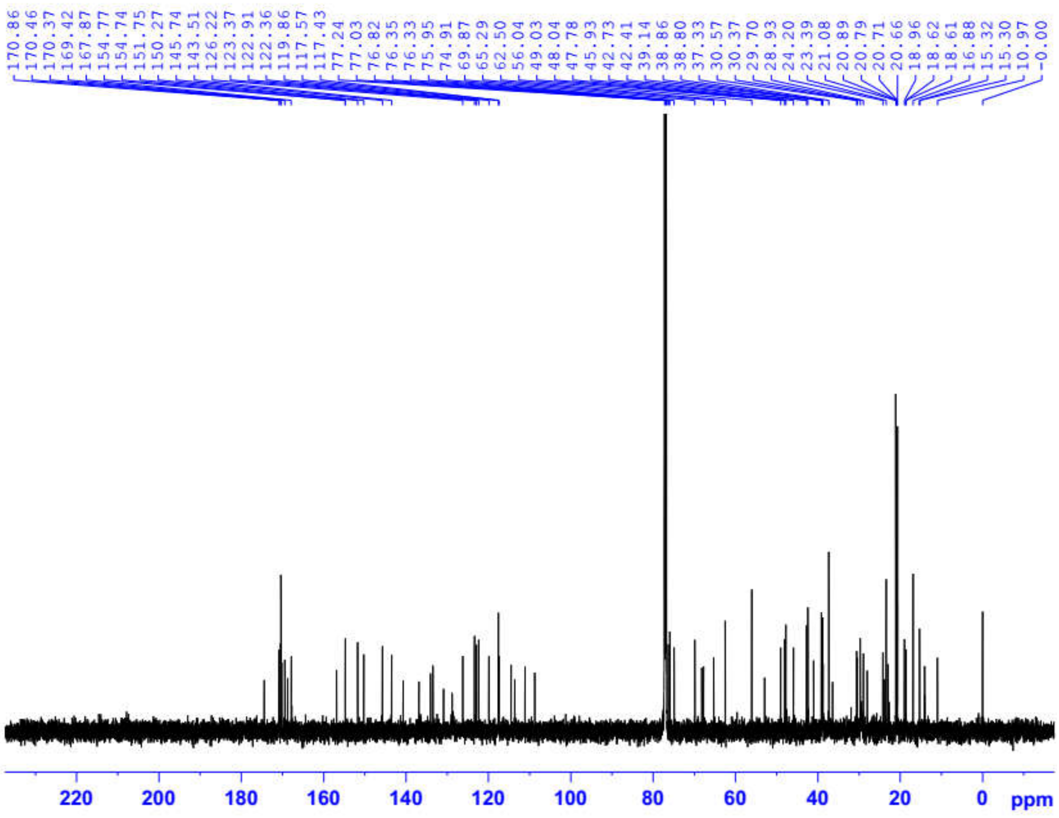

Figure S9. 1H and 13C NMR ($CDCl_3$, 600 MHz) spectra of compound **7**

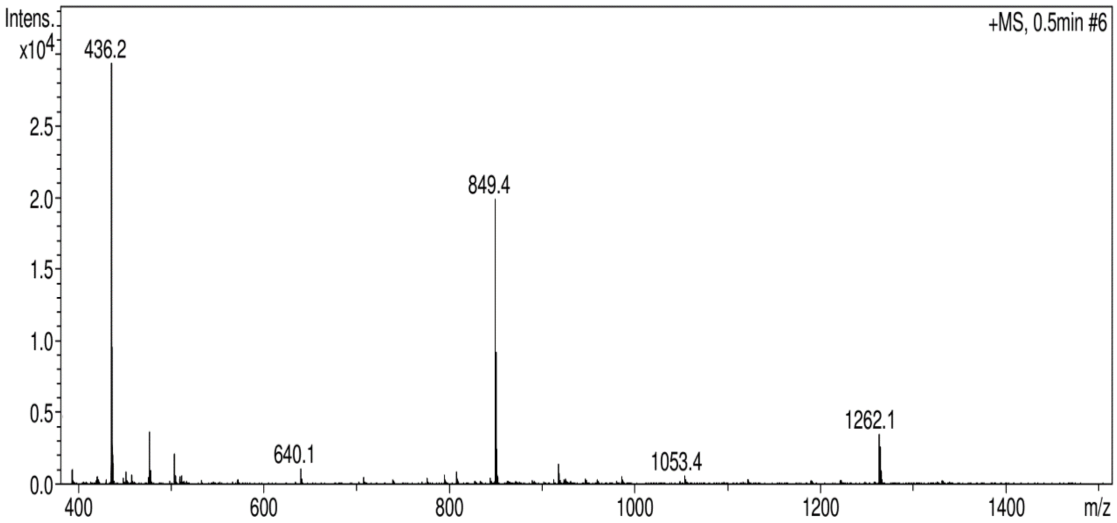

Figure S10. ESI-MS spectrum of compound **7**

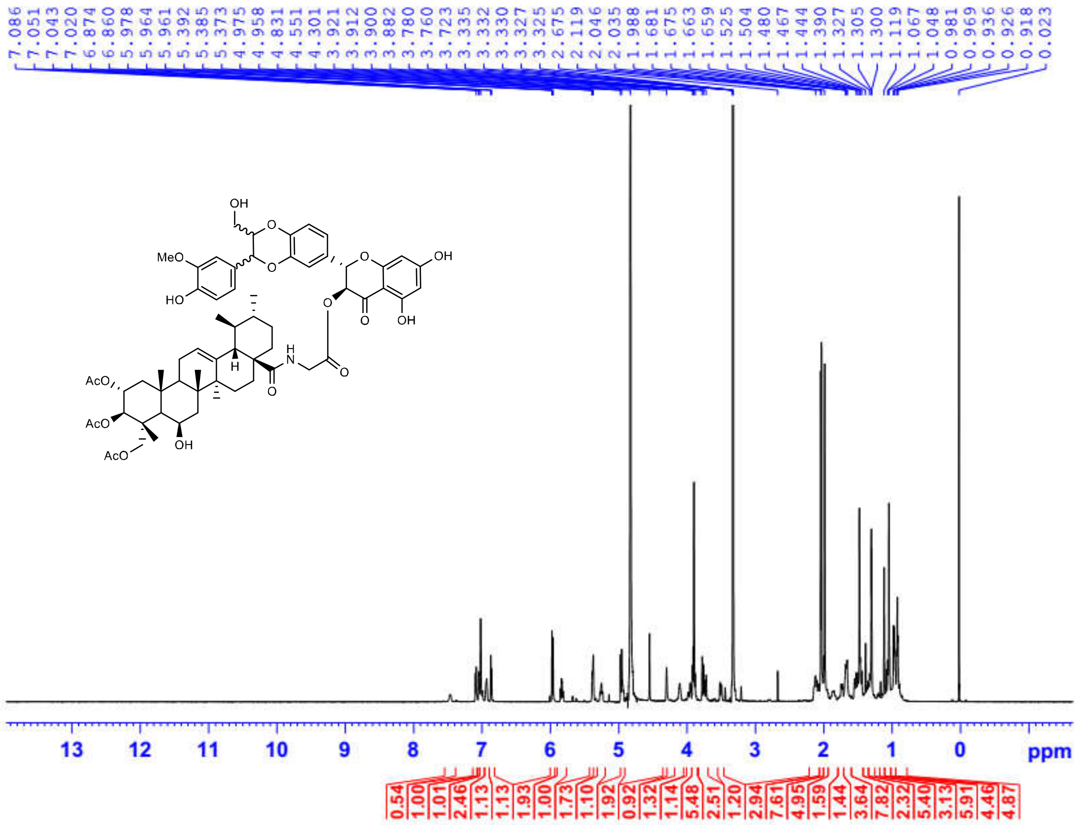

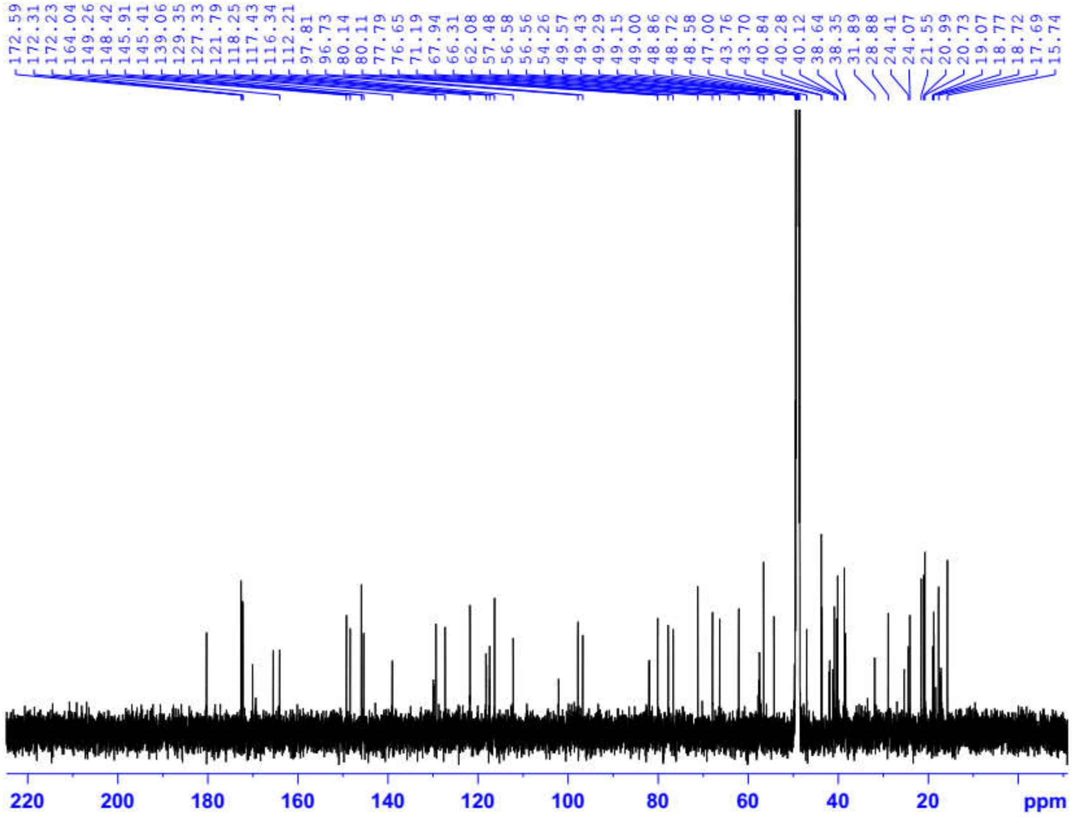

Figure S11. 1H and 13C NMR (CD_3OD, 600 MHz) spectra of compound **8**

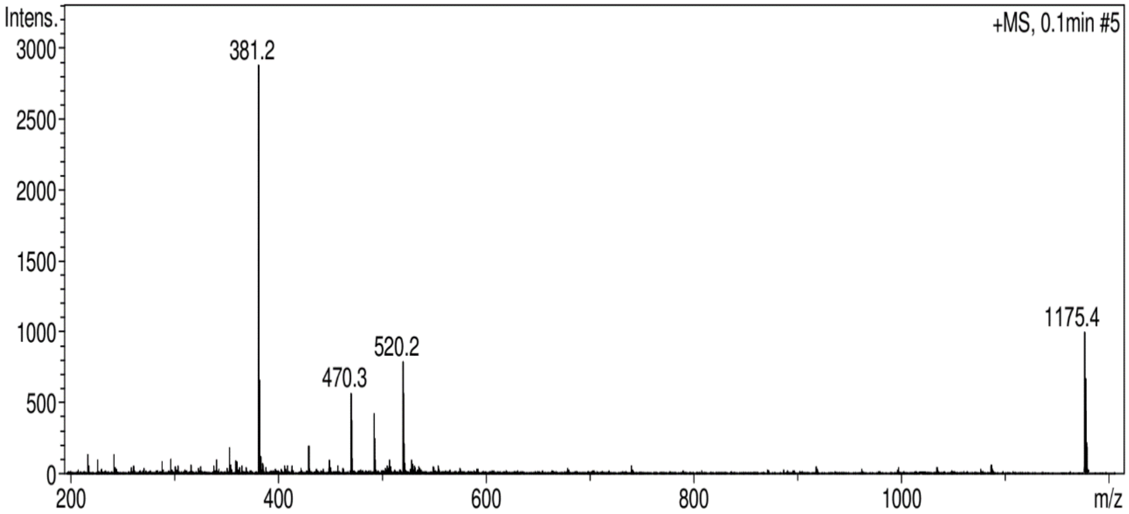

Figure S12. ESI-MS spectrum of compound **8**

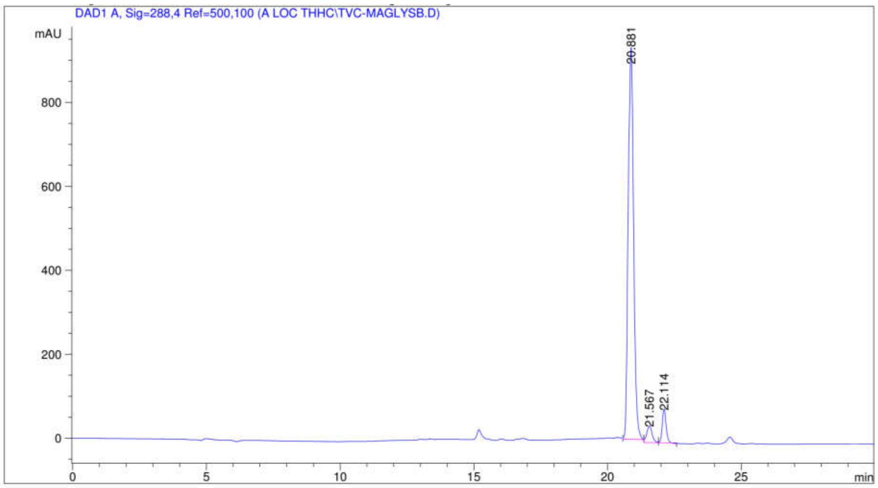


```
===================================================================
                       Area Percent Report
===================================================================

Sorted By             :      Signal
Multiplier            :      1.0000
Dilution              :      1.0000
Use Multiplier & Dilution Factor with ISTDs


Signal 1: DAD1 A, Sig=288,4 Ref=500,100
```

Peak #	RetTime [min]	Type	Width [min]	Area [mAU*s]	Height [mAU]	Area %
1	20.881	VV	0.2330	1.46603e4	943.20337	95.1972
2	21.567	VV	0.2272	343.86725	41.41860	2.2359
3	22.114	VB	0.1622	394.74591	82.19529	2.5668
Totals :				1.53789e4	1066.81726	

Figure S13. HPLC analysis of compound **8**

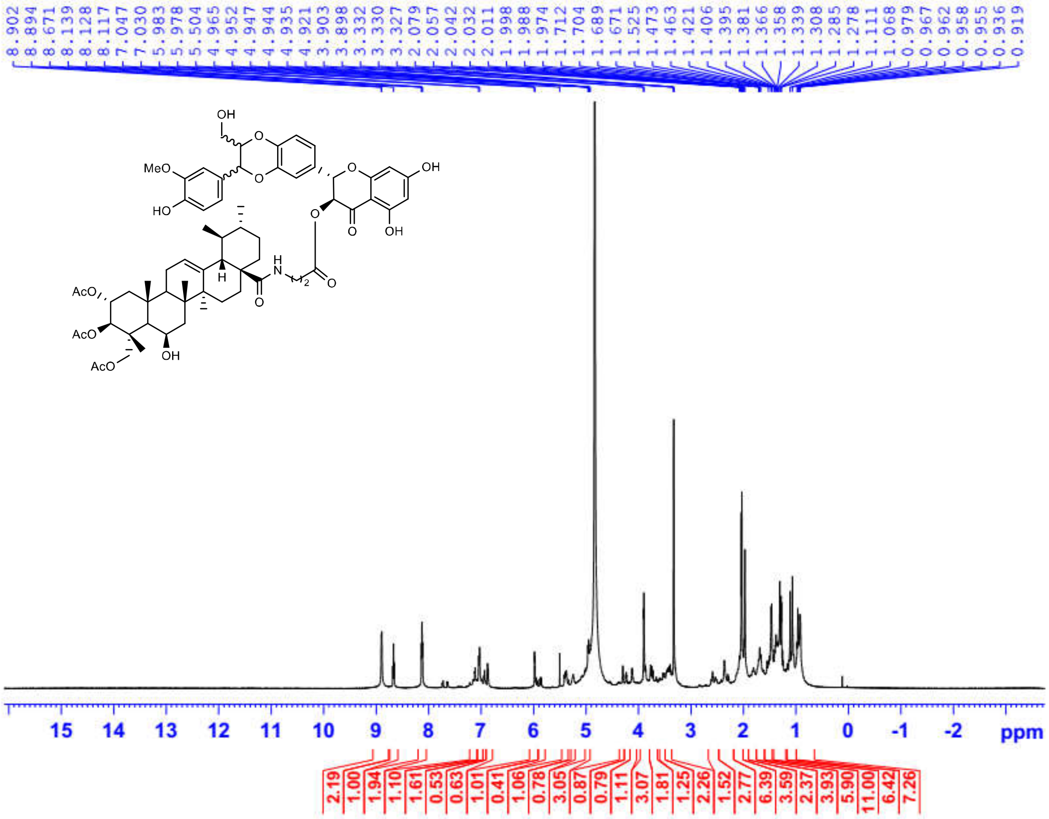

Figure S14. 1H NMR (CD_3OD, 600 MHz) spectrum of compound **9**

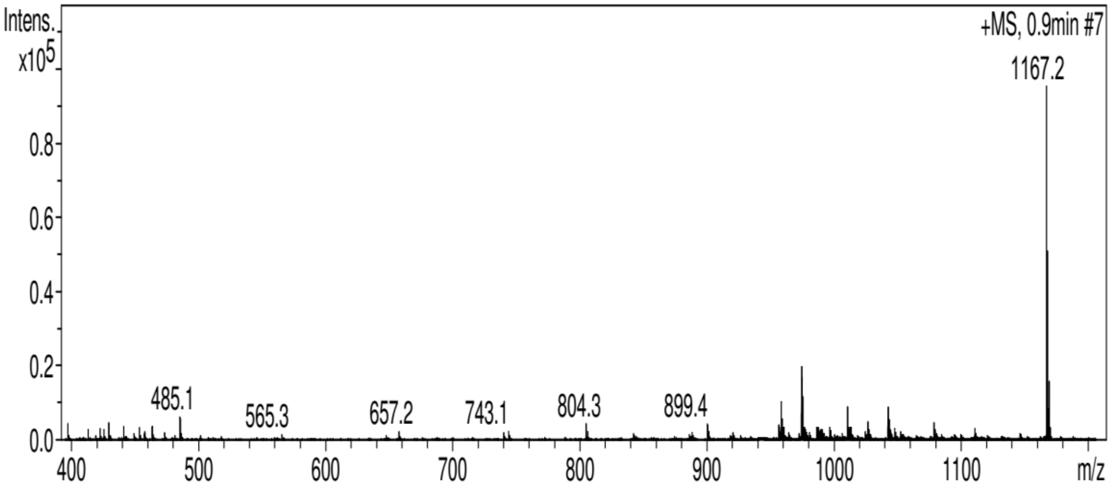

Figure S15. ESI-MS spectrum of compound **9**

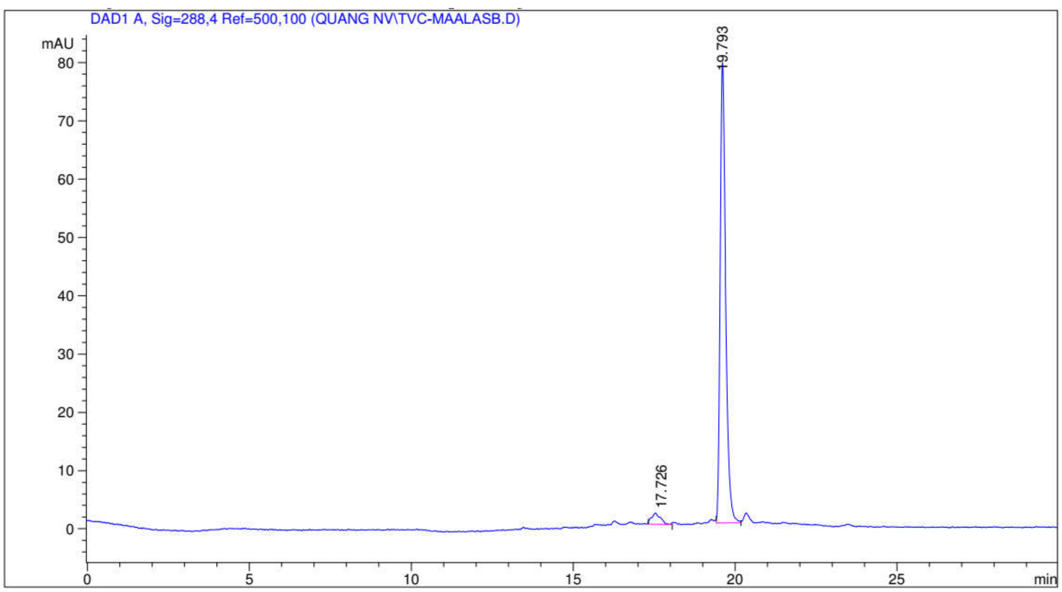


```
=====================================================================
                         Area Percent Report
=====================================================================

Sorted By             :      Signal
Multiplier            :      1.0000
Dilution              :      1.0000
Use Multiplier & Dilution Factor with ISTDs


Signal 1: DAD1 A, Sig=288,4 Ref=500,100

Peak RetTime Type  Width     Area      Height     Area
  #   [min]        [min]   [mAU*s]     [mAU]        %
----|-------|----|-------|----------|----------|--------|
   1  17.726 BV    0.2523   37.34469    1.92501   5.8241
   2  19.793 VB    0.1797  939.22766   79.58216  94.1759

Totals :                   976.57235   81.50717
```

Figure S16. HPLC analysis of compound **9**

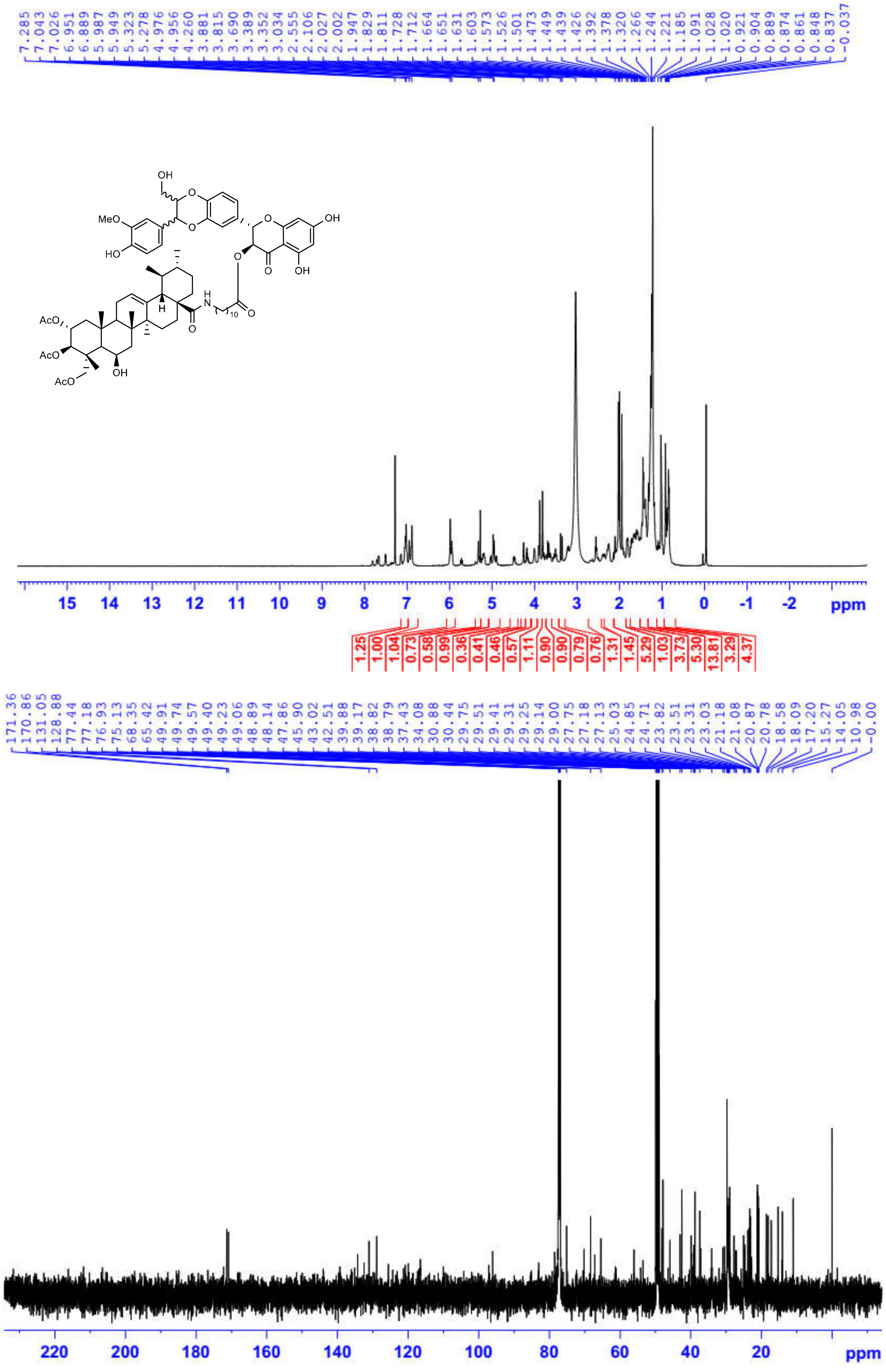

Figure S17. 1H and 13C NMR ($CDCl_3$, 500 MHz) spectra of compound **10**

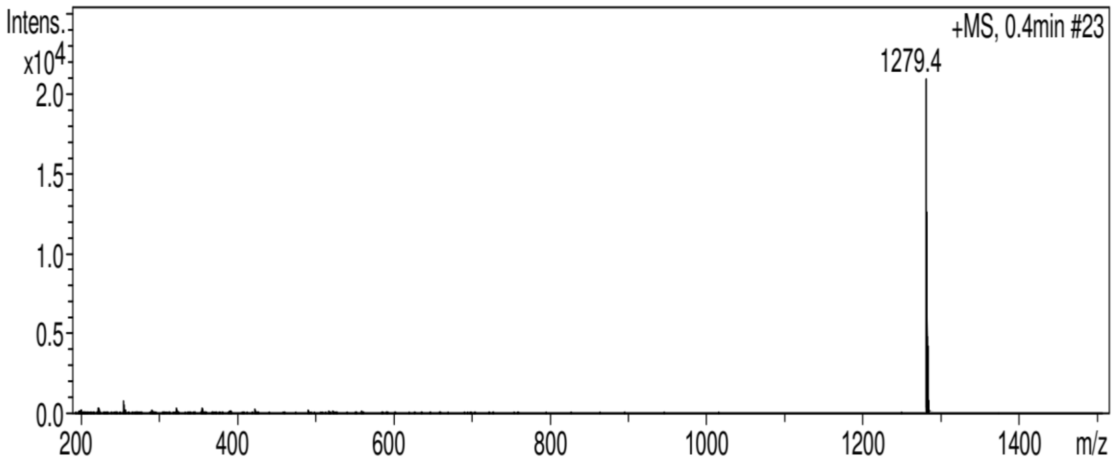

Figure S18. ESI-MS spectrum of compound **10**

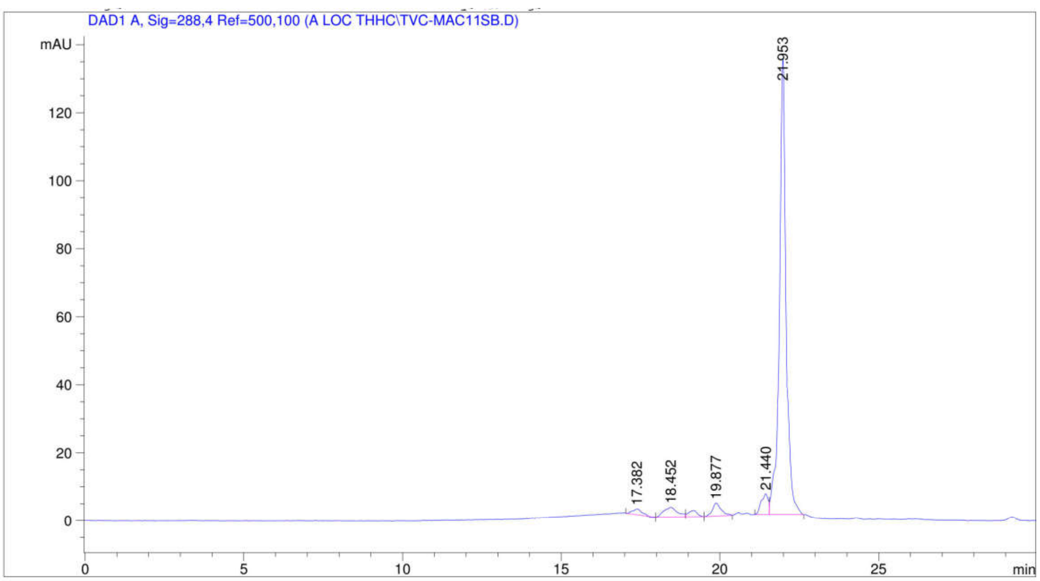

===
Area Percent Report
===

Sorted By : Signal
Multiplier : 1.0000
Dilution : 1.0000
Use Multiplier & Dilution Factor with ISTDs

Signal 1: DAD1 A, Sig=288,4 Ref=500,100

Peak #	RetTime [min]	Type	Width [min]	Area [mAU*s]	Height [mAU]	Area %
1	17.382	BB	0.2435	23.43523	1.68535	0.6321
2	18.452	BV	0.3712	58.01112	2.87418	1.5647
3	19.877	VV	0.2672	64.86910	3.87856	1.7497
4	21.440	BV	0.2092	74.20120	6.13362	2.0015
5	21.953	VV	0.2035	3486.84570	134.15177	94.0519

Totals : 3707.36235 148.72348

Figure S19. HPLC analysis of compound **10**

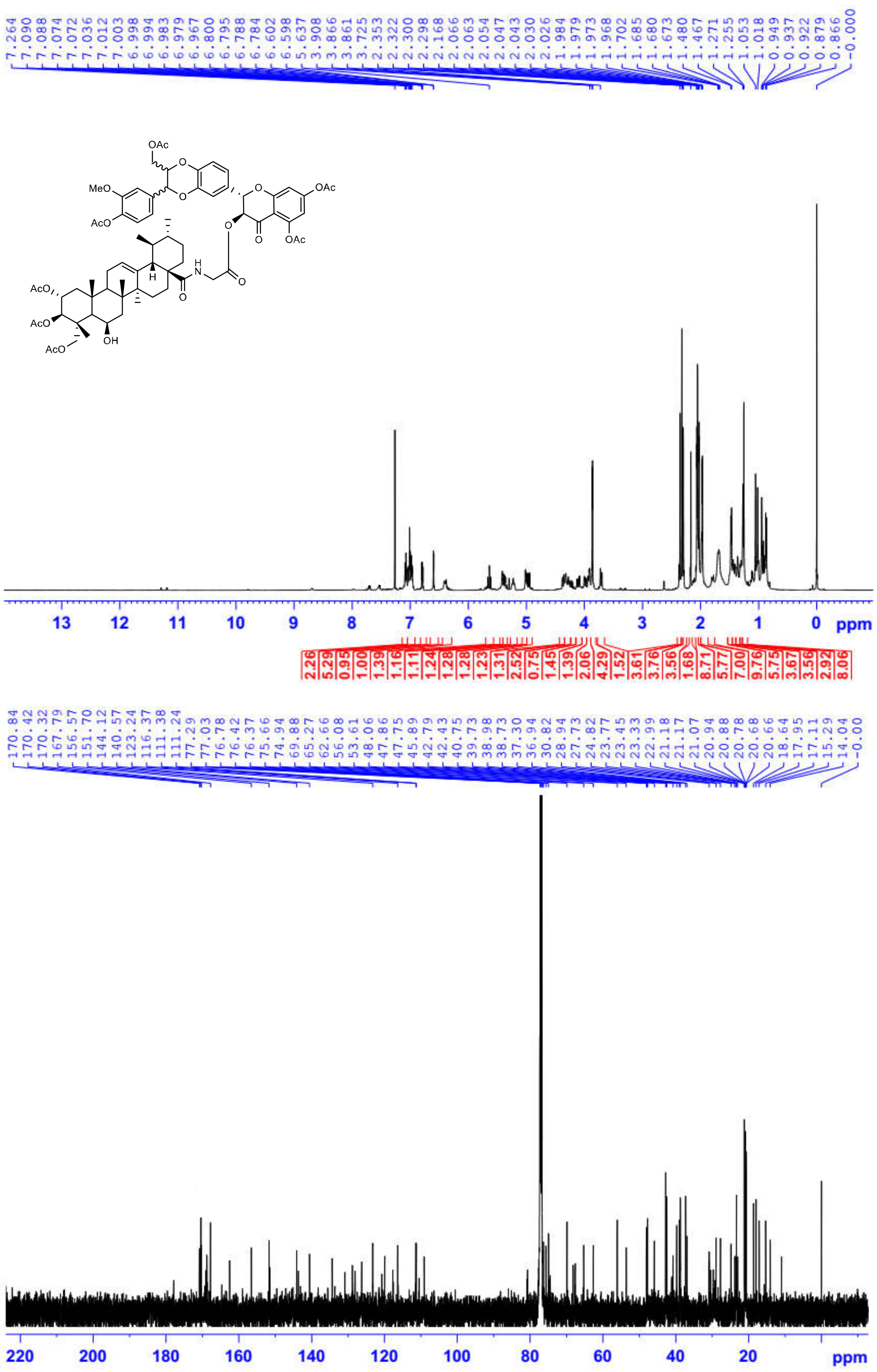

Figure S20. 1H and 13C NMR ($CDCl_3$, 500 MHz) spectra of compound **11**

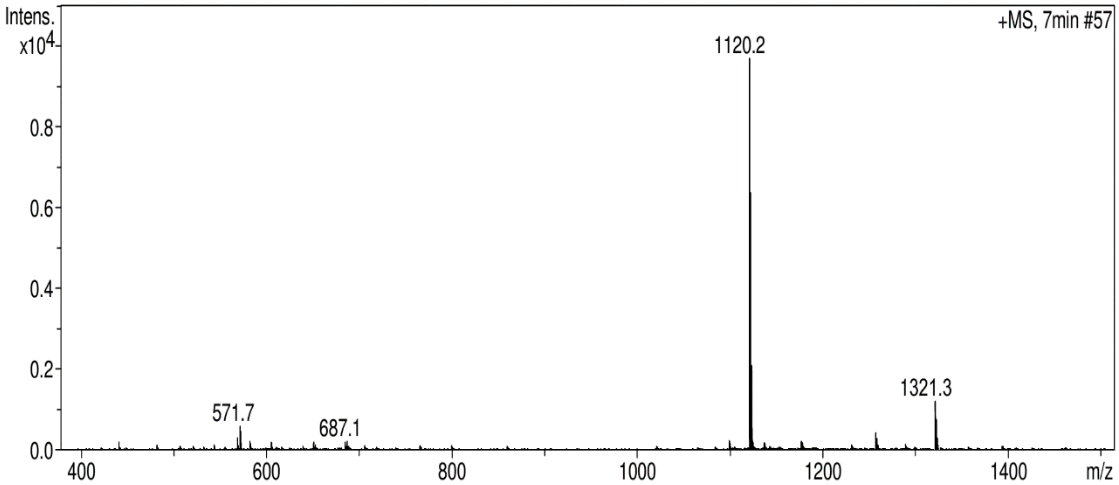

Figure S21. ESI-MS spectrum of compound **11**

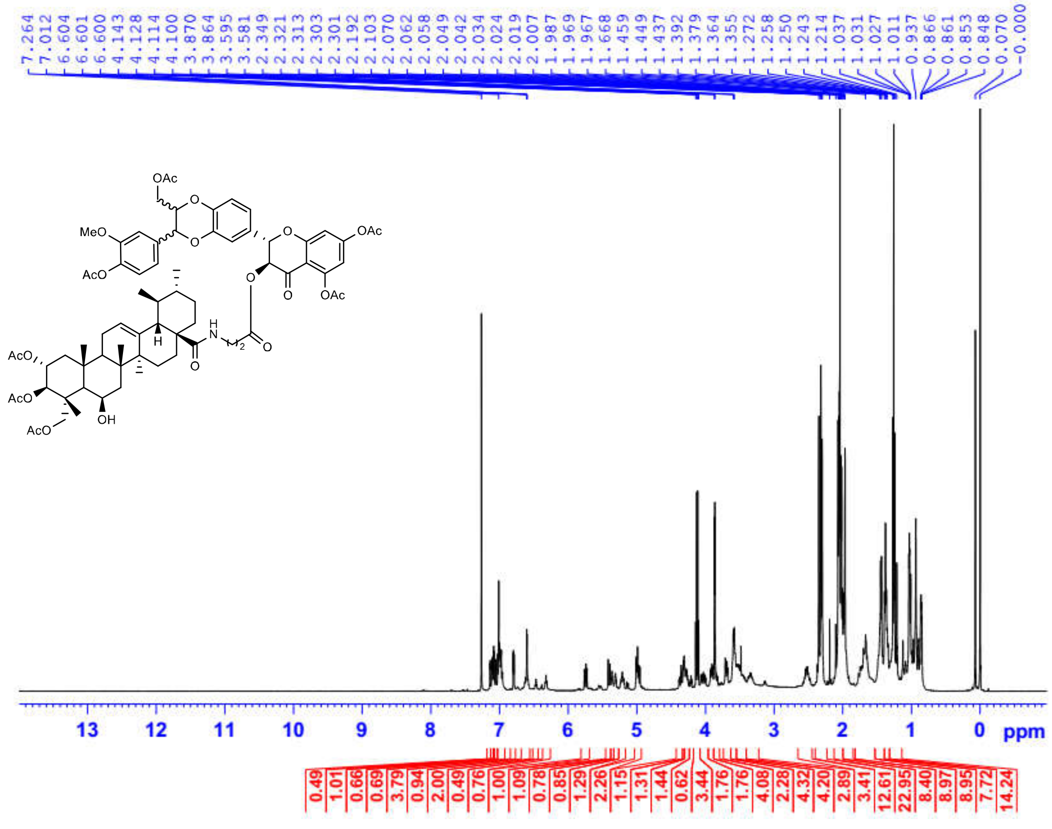

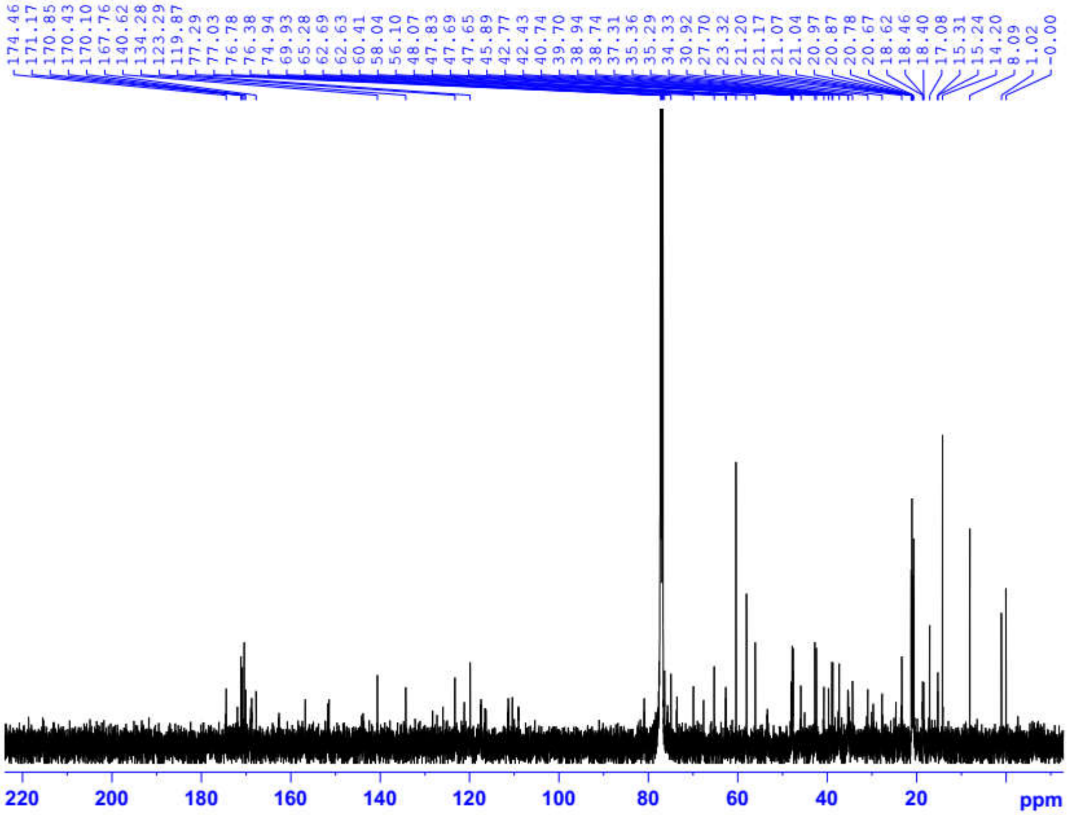

Figure S22. 1H and 13C NMR ($CDCl_3$, 500 MHz) spectra of compound **12**

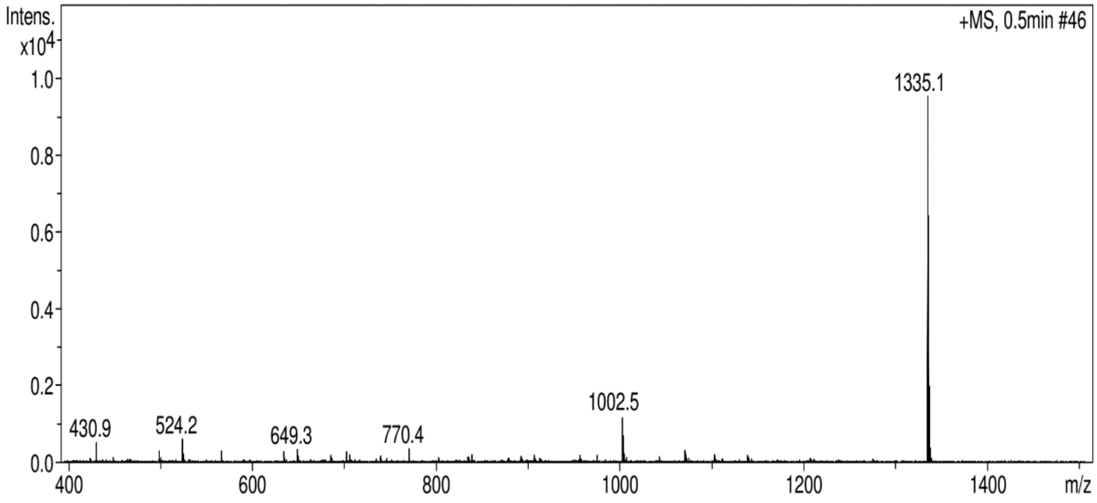

Figure S23. ESI-MS spectrum of compound **12**

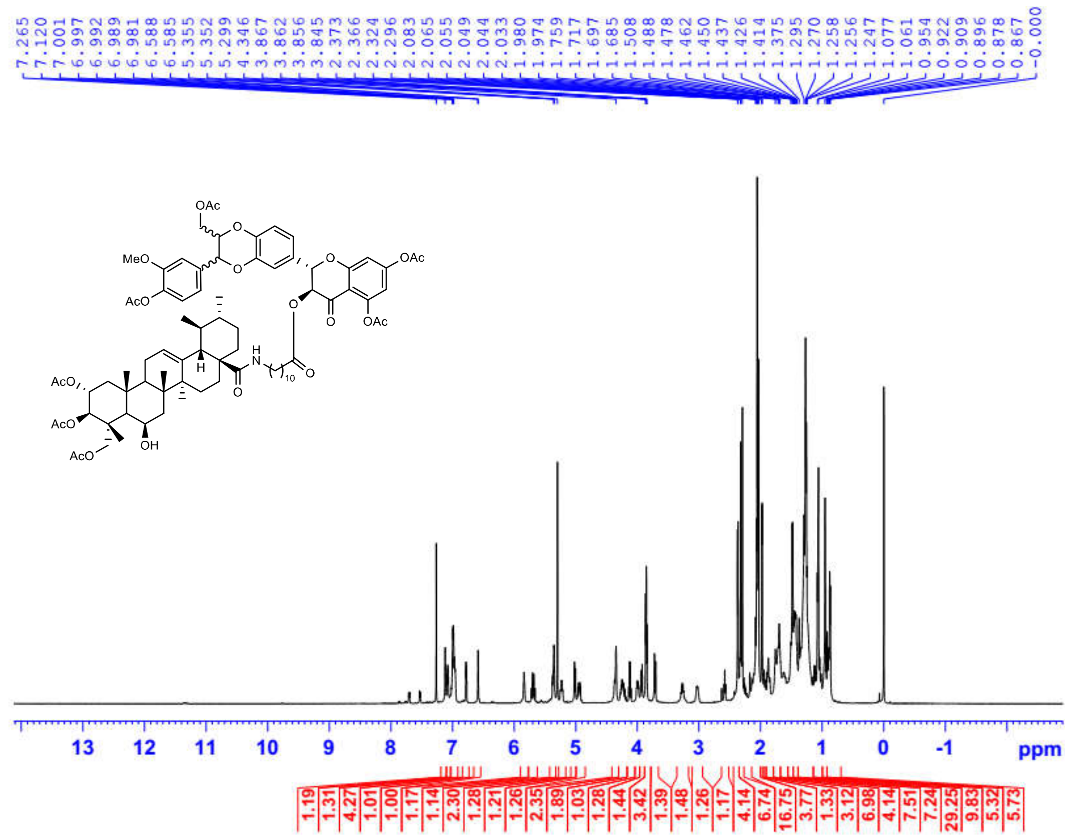

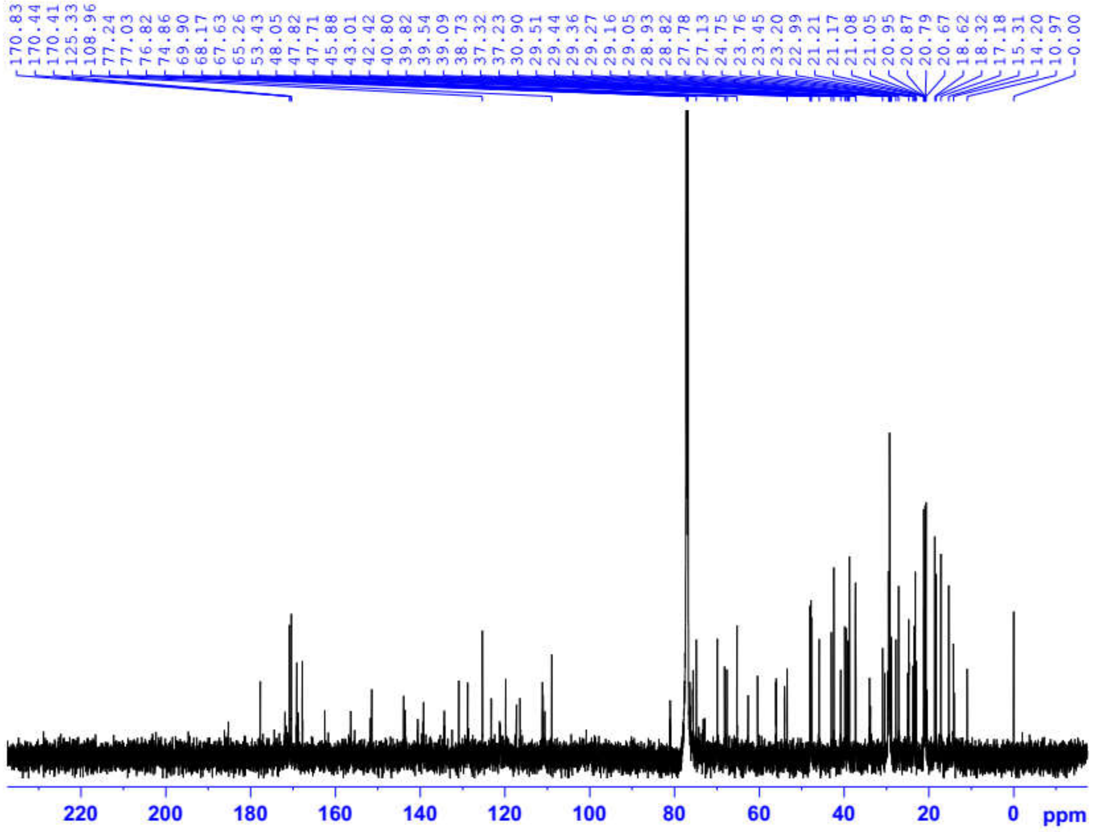

Figure S24. 1H and 13C NMR ($CDCl_3$, 500 MHz) spectra of compound **13**

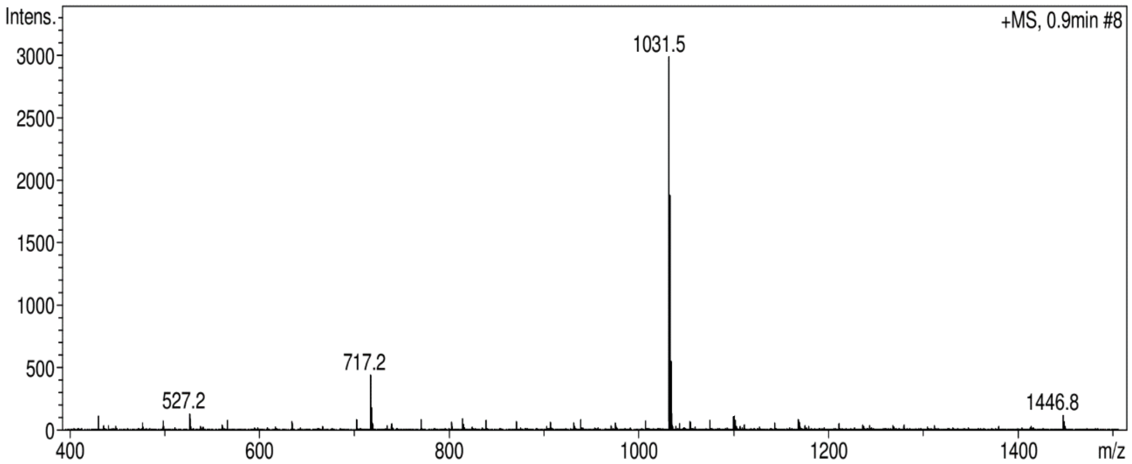

Figure S25. ESI-MS spectrum of compound **13**

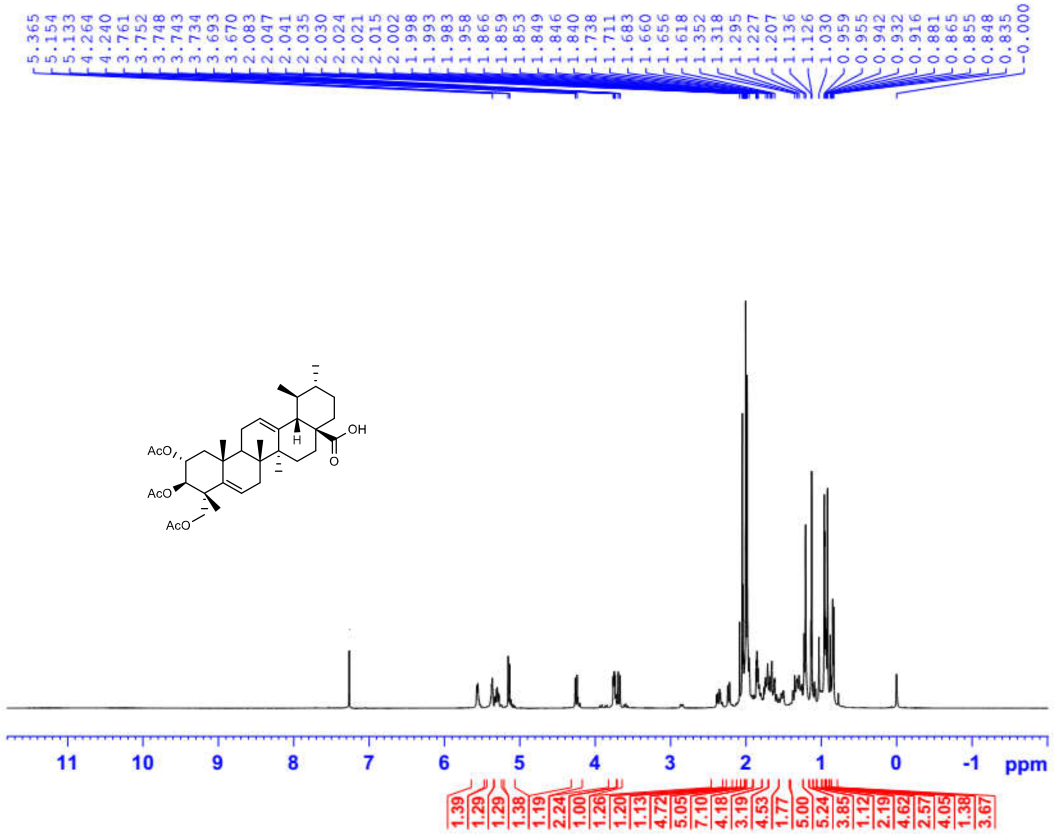

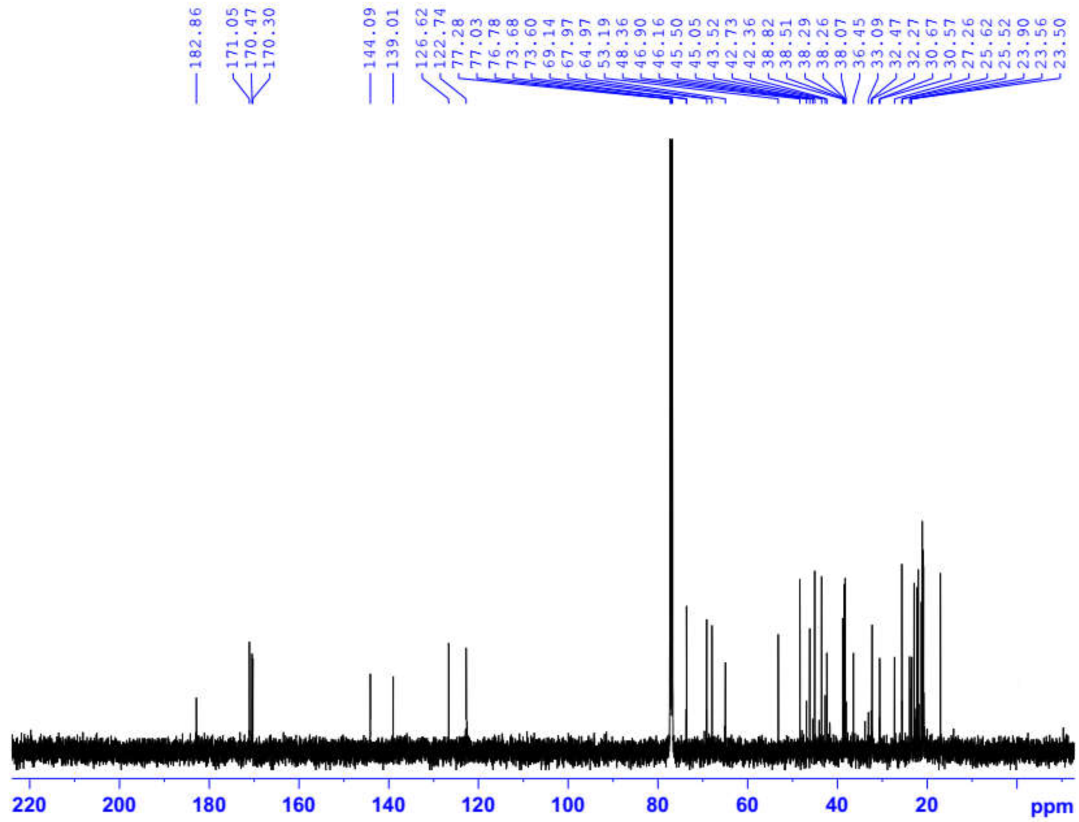

Figure S26. 1H and 13C NMR ($CDCl_3$, 500 MHz) spectra of compound **14**

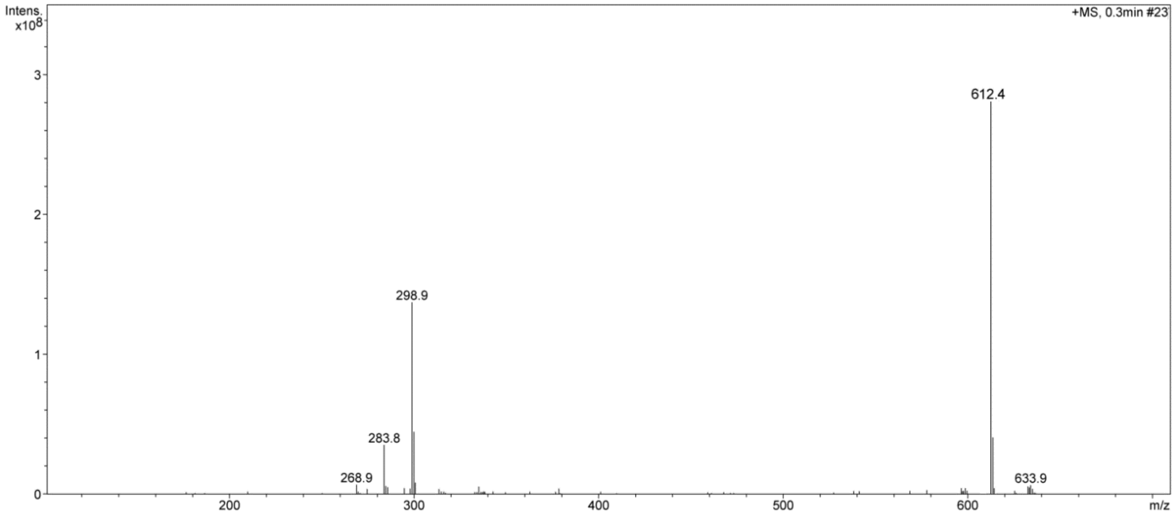

Figure S27. ESI-MS spectrum of compound **14**

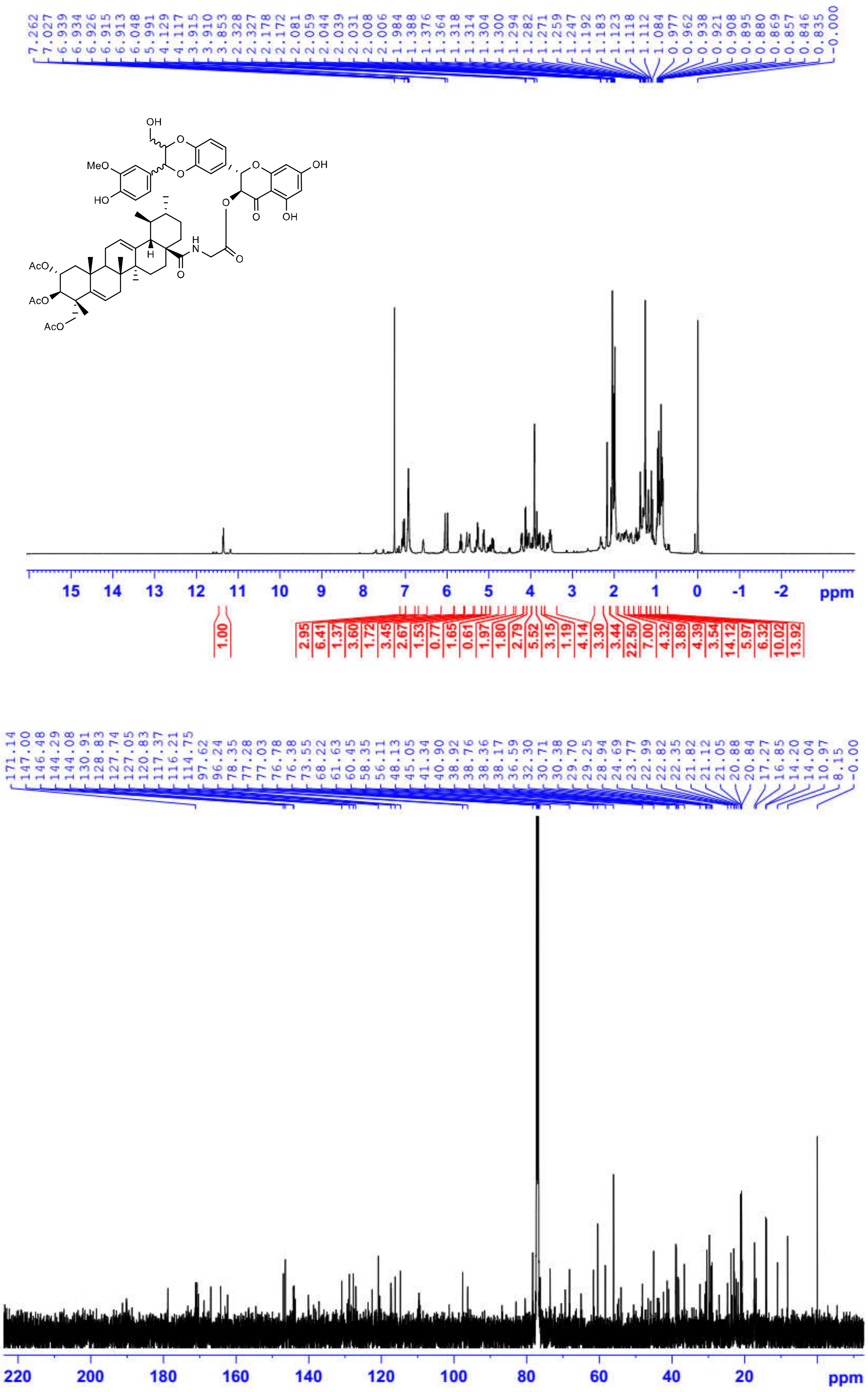

Figure S28. 1H and 13C NMR ($CDCl_3$, 500 MHz) spectra of compound **15**

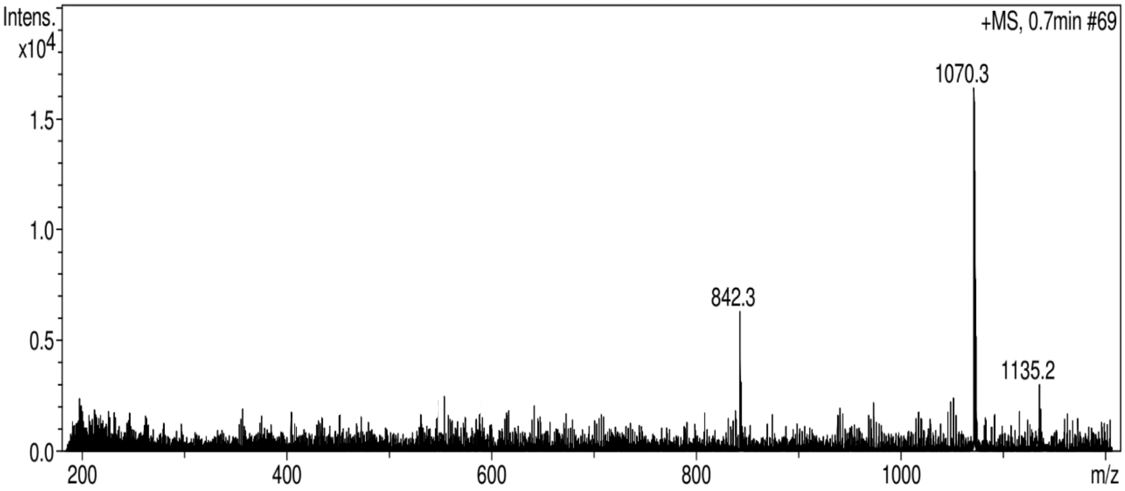

Figure S29. ESI-MS spectrum of compound **15**

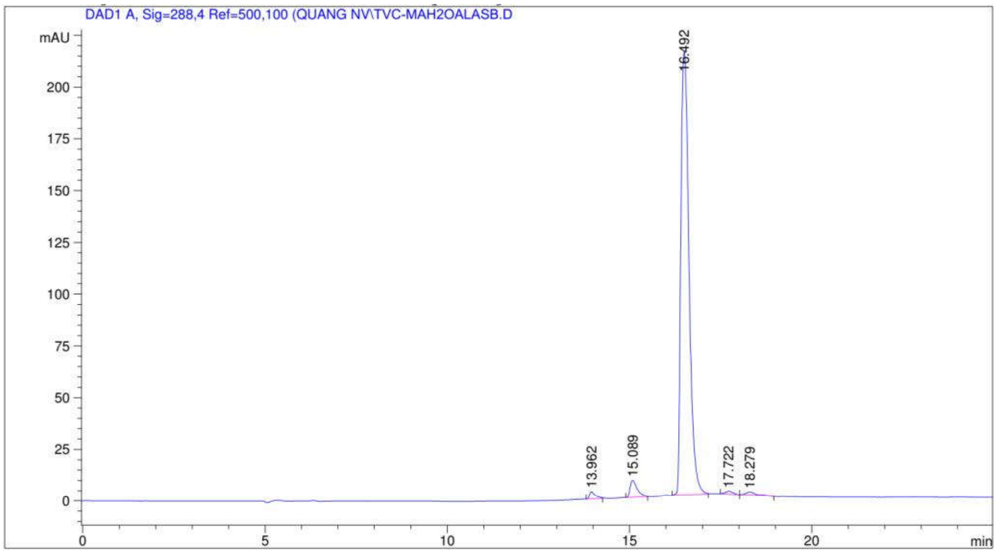

Area Percent Report

Sorted By : Signal
Multiplier : 1.0000
Dilution : 1.0000
Use Multiplier & Dilution Factor with ISTDs

Signal 1: DAD1 A, Sig=288,4 Ref=500,100

Peak #	RetTime [min]	Type	Width [min]	Area [mAU*s]	Height [mAU]	Area %
1	13.962	BB	0.1643	40.25041	4.57196	1.1436
2	15.089	BB	0.2027	110.67388	7.26386	3.1445
3	16.492	BB	0.2468	3316.17288	213.93143	94.2195
4	17.722	BV	0.2370	23.55582	1.36626	0.6693
5	18.279	VB	0.2751	28.97230	1.56911	0.8232
Totals :				3519.62529	228.70263	

Figure S30. HPLC analysis of compound **15**

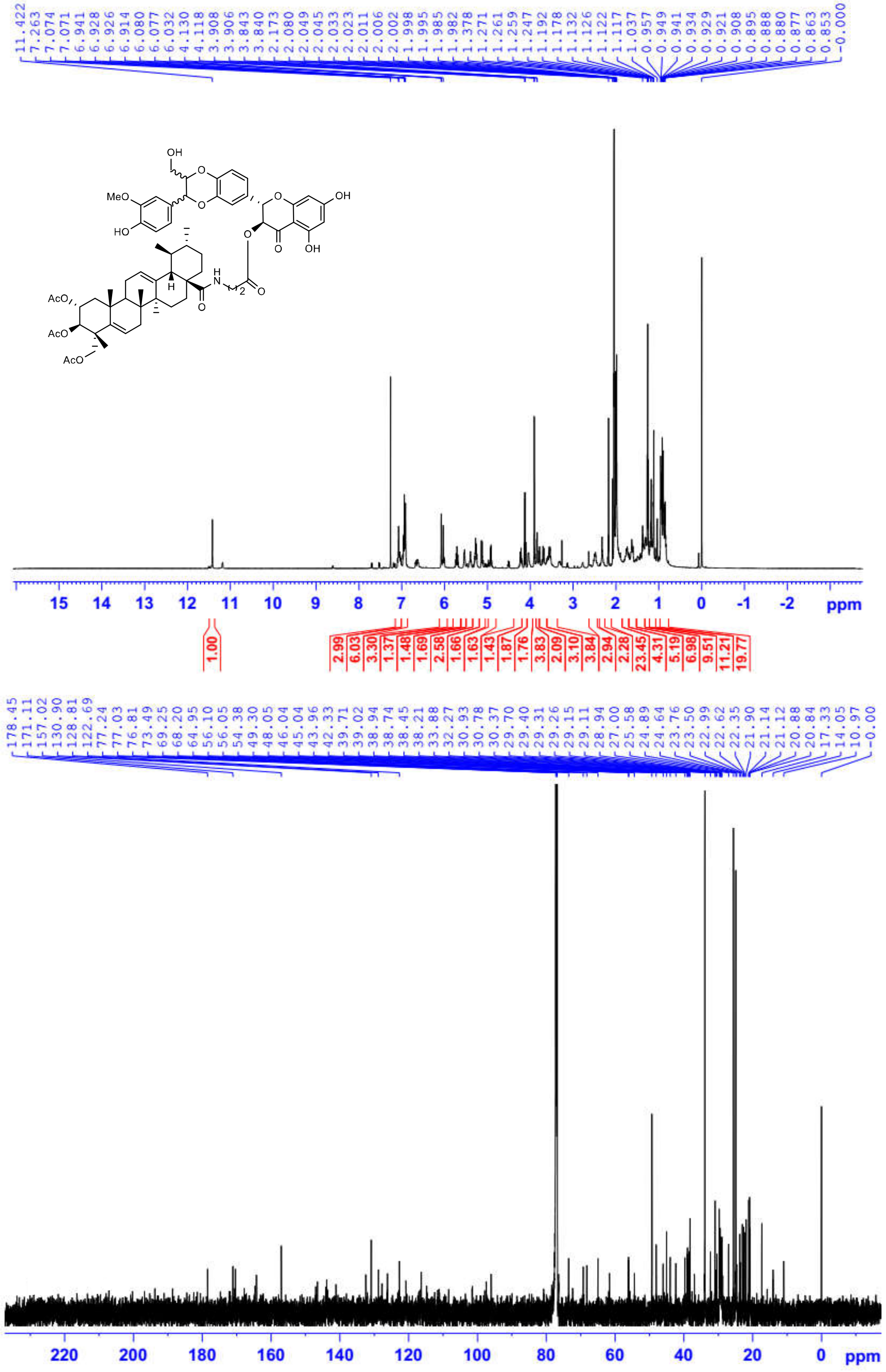

Figure S31. 1H and 13C NMR ($CDCl_3$, 500 MHz) spectra of compound **16**

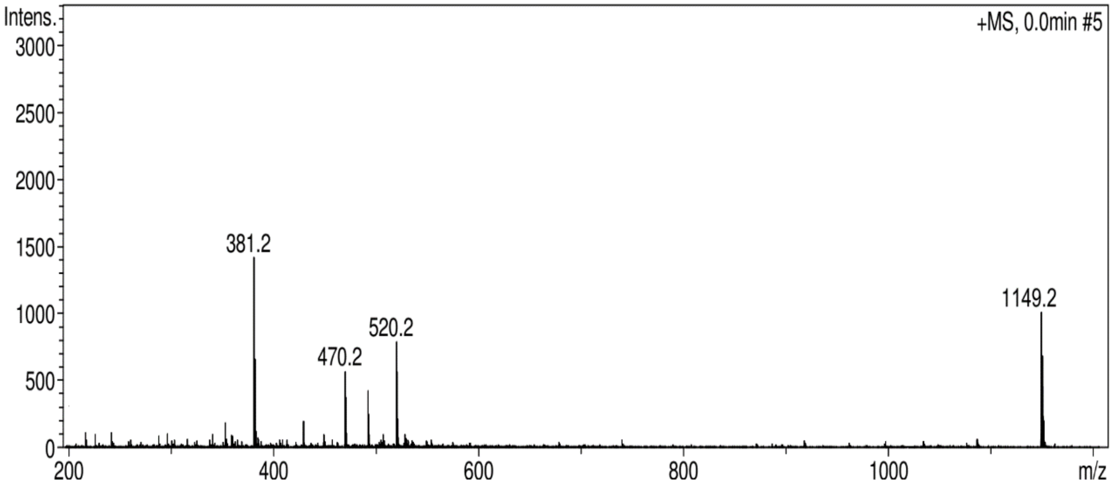

Figure S32. ESI-MS spectrum of compound **16**

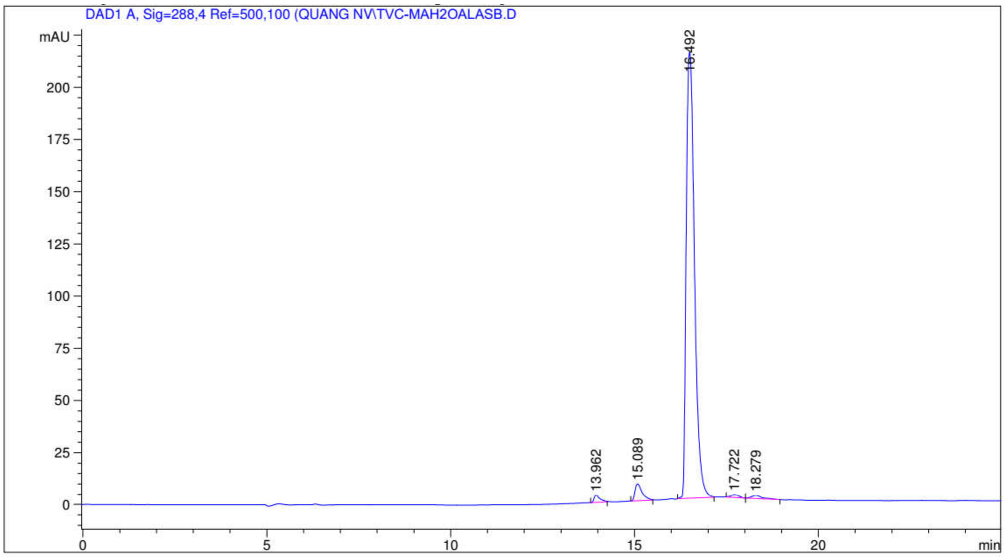

===

Area Percent Report

===

Sorted By : Signal
Multiplier : 1.0000
Dilution : 1.0000
Use Multiplier & Dilution Factor with ISTDs

Signal 1: DAD1 A, Sig=288,4 Ref=500,100

Peak #	RetTime [min]	Type	Width [min]	Area [mAU*s]	Height [mAU]	Area %
1	13.962	BB	0.1643	40.25041	4.57196	1.1436
2	15.089	BB	0.2027	110.67388	7.26386	3.1445
3	16.492	BB	0.2468	3316.17288	213.93143	94.2195
4	17.722	BV	0.2370	23.55582	1.36626	0.6693
5	18.279	VB	0.2751	28.97230	1.56911	0.8232

Totals : 3519.62529 228.70263

Figure S33. HPLC analysis of compound **16**

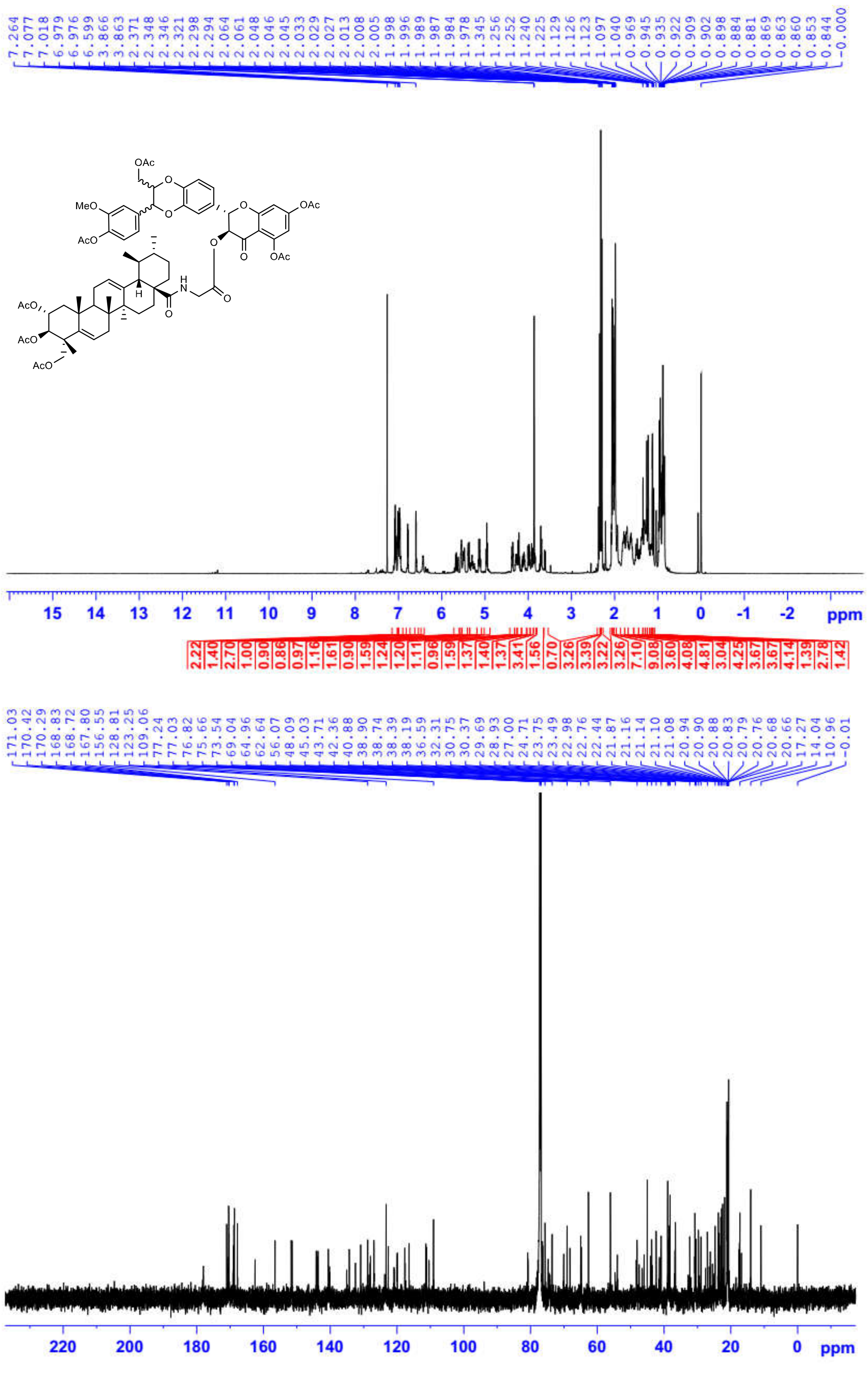

Figure S 34. 1H and 13C NMR ($CDCl_3$, 500 MHz) spectra of compound **17**

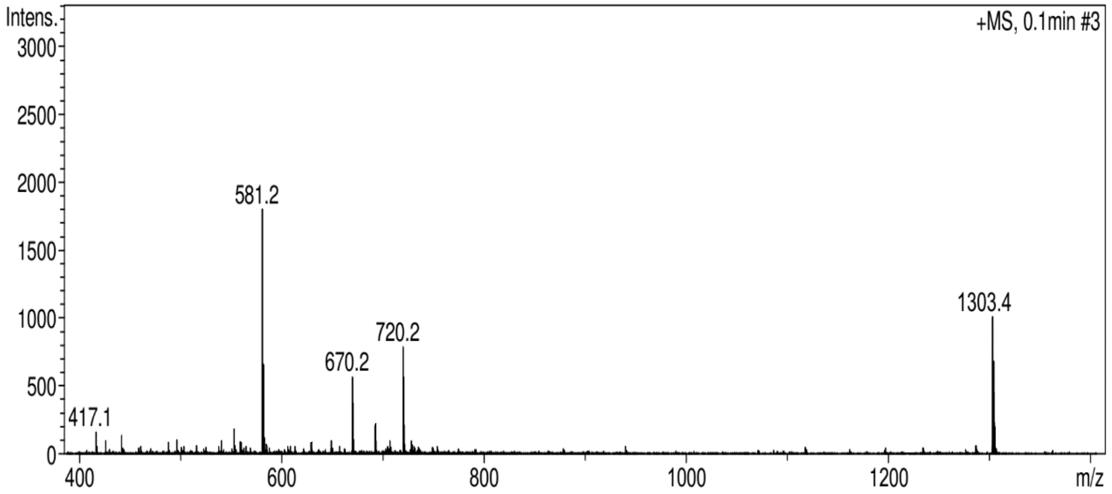

Figure S35. ESI-MS spectrum of compound **17**

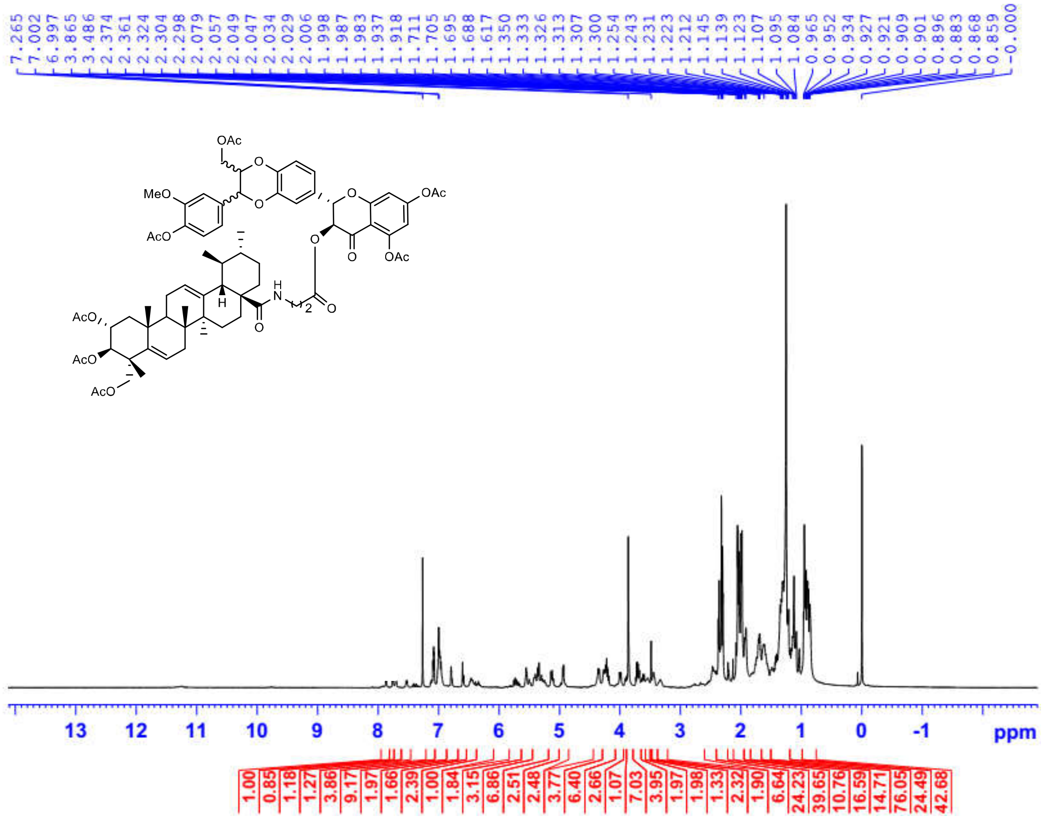

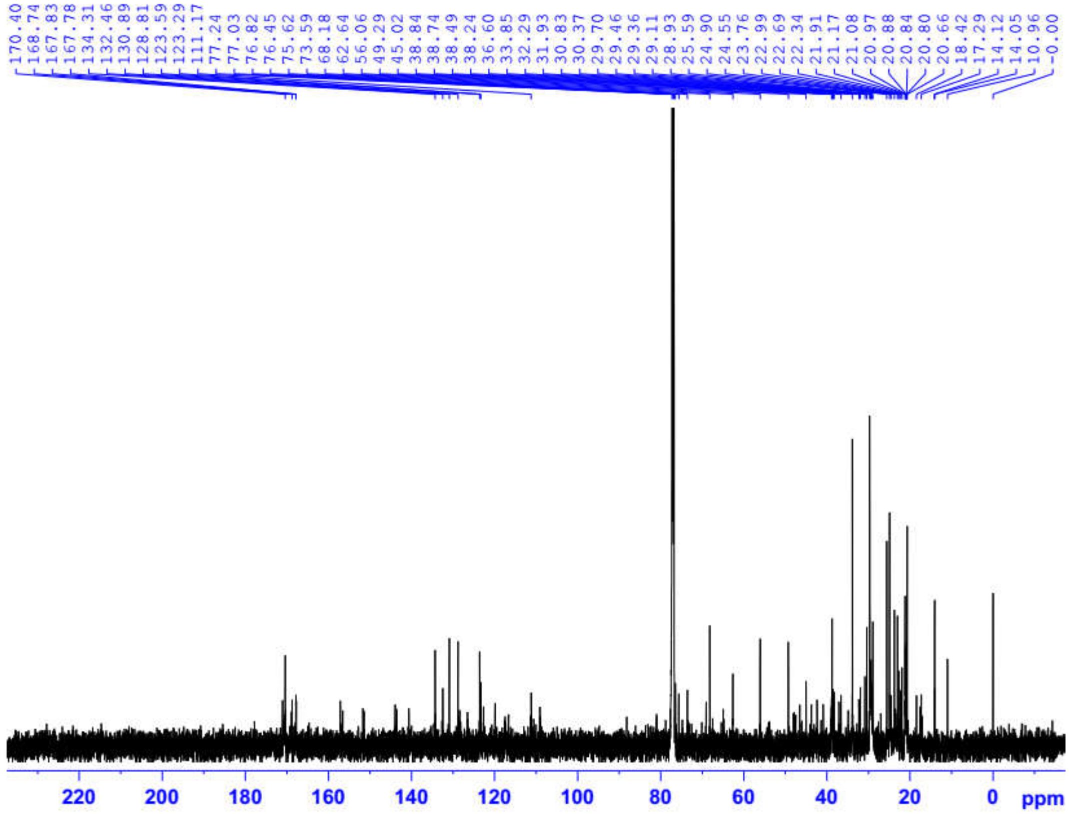

Figure S36. 1H and 13C NMR ($CDCl_3$, 500 MHz) spectra of compound **18**

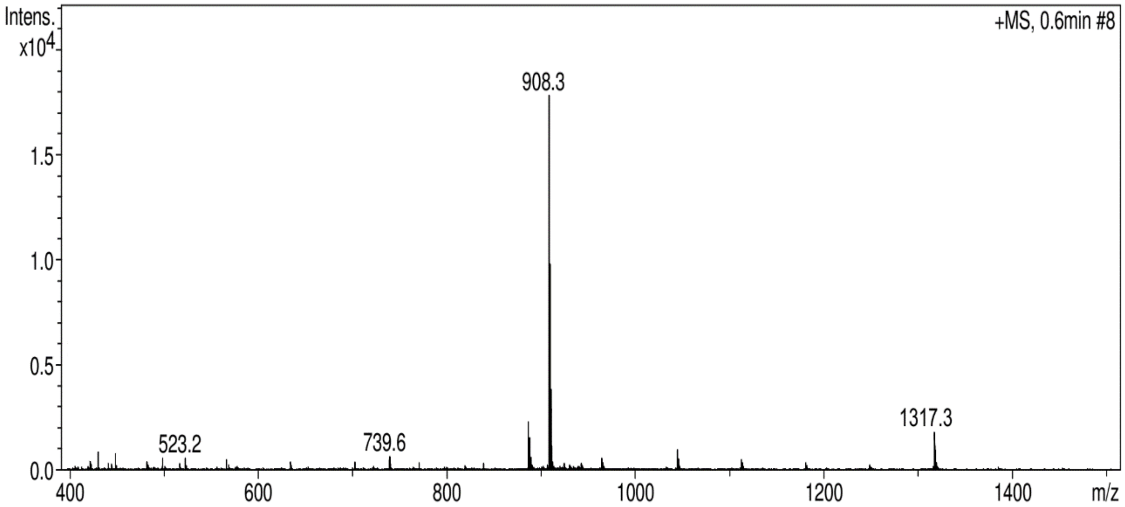

Figure S37. ESI-MS spectrum of compound **18**
